# Decoding the anti-inflammatory properties of sesquiterpene coumarins from the active fractions of *Ferula assa-foetida* through integrated experimental and in silico analyses

**DOI:** 10.1038/s41598-025-26010-3

**Published:** 2025-11-26

**Authors:** Mubarak A. Alamri, Gamal A. Soliman, Mohammed A. Alamri, Rehab F. Abdel-Rahman, Ahmed W. Alhalabi, Raid S. Almahmoud, Ibrahim M. Alsagheir, Abdulrahman G. Alharbi, Maged S. Abdel-Kader

**Affiliations:** 1 Department of Pharmaceutical Chemistry, College of Pharmacy, Prince Sattam Bin Abdulaziz University, 11942 Al-Kharj, Saudi Arabia; 2 Department of Pharmacology and Toxicology, College of Pharmacy, Prince Sattam Bin Abdulaziz University, 11942 Al-Kharj, Saudi Arabia; 3 Department of Pharmacology, National Research Centre, Giza, 12622 Egypt; 4 College of Pharmacy, Prince Sattam Bin Abdulaziz University, 11942 Al-Kharj, Saudi Arabia; 5 Maternity and Children’s Hospital, Ministry of Health, 11942 Al-Kharj, Saudi Arabia; 6 Department of Pharmacognosy, College of Pharmacy, Prince Sattam Bin Abdulaziz University, 11942 Al-Kharj, Saudi Arabia

**Keywords:** Asafoetida, Sesquiterpene coumarins, Anti-inflammatory, Network pharmacology, Molecular docking, Molecular dynamic

## Abstract

*Ferula assa-foetida* known as Asafoetida has a long-established history in folk medicine as a therapeutically useful drug in many disorders. The current paper explored the in vivo and in vitro anti-inflammatory activities of the plant. Liquid ethanol crude extract was subjected to liquid-to-liquid extraction, and the fractions so obtained were evaluated against the carrageenan-induced acute paw edema model in rats, to determine the anti-inflammatory effects. The fractions that gave the greatest activity-ethyl acetate and hexane- afforded four sesquiterpene coumarins: auraptene (**1**), umbelliprenin (**2**), galbanic acid (**3**), and kamolonol (**4**). The overall structure was determined through detailed spectroscopic investigation, ^1^H NMR, ^13^C NMR, DPET135, COSY, HSQC, HMBC, HRESIMS, and other references to the literature. Evaluation of the in vitro activity of the LPS stimulated RAW 264.7 macrophages showed that the compound **4** (ethyl acetate fraction) exhibited maximum inhibitory action against the generation of nitric oxide (NO), followed by the compounds **1** and **2** of the hexane fraction. Network Pharmacology, Molecular Docking and Molecular dynamic simulation analysis were used to identify the target genes and explain the potential differences between the active compounds. As far as the authors know, there are no earlier reports that mention the anti-inflammatory action of kamolonol (**4**).

**Supplementary Information:**

The online version contains supplementary material available at 10.1038/s41598-025-26010-3.

## Introduction

Inflammation is the first line of defense of the body in response to infection or tissue damage resulting in organ structural stability, normal physiology and homeostasis within the injured tissue^1^. Over the past few years, it has become one of the most prominent objects of biomedical research in the world and this is mostly because it is clearly linked to a vast diversity of human illnesses^2^. The commonly used medical treatments of inflammatory disorders include glucocorticoids and nonsteroidal anti-inflammatory drugs (NSAIDs). These medications are linked with some adverse effects such as high blood pressure, stomach pain and ulcers, fragile bones, kidney failure, Cushing’s syndrome, diabetes, and vulnerability to infections^3^. There is therefore urgent need to establish safer and more effective methods of handling inflammatory pathology.

The process of inflammation is a complicated list of physiological events whereby a network of cellular events and a coordinated sequence of molecular events orchestrate immune activation and maintain homeostasis in tissues. The core of this process involves the cyclooxygenase (COX) pathway, in which COX-1 regulates and controls normal physiologic activity, at the same time as COX-2 is preferentially induced during an inflammatory event, to promote the constructive formation of prostaglandins, and hence exaggerate the inflammatory response^4,5^. The (NF-κB) signaling pathway is major transcriptional regulator of pro-inflammatory effectors including tumor necrosis factor-alpha TNF-α ,interleukin-6 (IL-6) in agreement with this system. NF-κB activation begins with proteolytic removal of anti- inflammatory proteins (IκB), subsequently permitting the process of translocation of the complex to the nucleus to facilitate transmission of inflammation-linked genes^6,7^. Moreover, a balance between pro-inflammatory cytokines, such as IL-1b, IL-6 and TNF-α, and anti-inflammatory molecules including IL-10 is important to regulate the attraction of immune cells and control excessive inflammatory processes^8^. Such complexly interlinked web of molecular networks forms the ideal target of therapeutic interventions in inflammatory disorders. Traditional medicine has great repositories of bioactive secondary metabolites present in herbs that can be used to draw out pharmacologically useful agents. Therefore, plant-based preparations still serve as a major source in the discovery of new drugs. Genus *Ferula* L. (with over 170 species formally described) is the third largest in its family. Most representatives of this genus are well represented in ethnomedicine, and several of them have proved to be significant sources of biologically active materials^9^.

Asafoetida is the oleo-gum-resin secreted by the roots of *Ferula assa-foetida*, traditionally used to treat a wide range of conditions including whooping cough, bronchitis, asthma, influenza, stomachache, flatulence, ulcers, intestinal parasites, spasms, digestive weakness, and epilepsy^10^.

Multiple biological activities were reported for essential oils and extracts of the genus, including anticancer, antioxidant, anticonvulsant, antinociceptive, antidepressant, aphicidal, antiprotozoal, anthelmintic, antibacterial, anti-inflammatory, antihypertensive, antiulcer, and antispasmodic activities. In addition, asafoetida expressed many biological activities including antiviral, antifungal, molluscicidal, antidiabetic, antiobesity, hepatoprotective, neuroprotective, memory enhancing, and digestive aid^10–12^. The extract containing ethanol was rich in the anti-inflammatory action on the tumor necrosis factor-alpha (TNF-α–) stimulated human umbilical vein endothelial cells (HUVECs)^3^.

Phytochemically, *Ferula* species are rich in the bioactive sesquiterpene coumarins derivatives^13–17^. Although sesquiterpene coumarins are present in 12 plant genera, they are mainly reported from *Ferula* species^13^. Many members of the sesquiterpene coumarin family were reported to have anti-inflammatory activity^13–17^. Two sesquiterpenes with anti-inflammatory activity were reported from *F. hermonis*^14^. Sesquiterpene coumarins were reported from the aerial parts of *F. sinkiangensis* expressed anti-inflammatory activities in lipopolysaccharide-stimulated RAW 264.7 macrophages^15^. Anti-neuroinflammatory sesquiterpene coumarins were identified from the resin of *F. sinkiangensis* and the whole plants of *F. bungeana*^16,17^.

The current study was targeted at investigating the anti-inflammatory properties of assafoetida, both in vivo, in vitro, in silico and identifying the active metabolites responsible for this effect via biologically guided phytochemical study. We also aim to identify the potential molecular targets and mechanisms of action of its isolated compounds.

## Results

### Characterization of the isolated compounds

*Auraptene* (**1**): pale yellow solid, UV λ_max_ MeOH: 226, 290, 322 nm, ^1^H and ^13^C NMR: Tables 1, 2, Figures S1–S10, High Resolution Electrospray Ionization Mass Spectrometry (HRESIMS) *m/z* [M-H]^+^ 297.1483 (calcd for C_19_H_22_O_3_-H, 297.1500), [M + H]^+^ 299.1640 (calcd for C_19_H_22_O_3_ + H, 299.1647) (Figures S11, S12).

**Table 1:** ^1^H-NMR data (δ ppm, *J* in parentheses in Hz) of compounds 1–4 in C_6_D_6_.

	1	2	3	3*	4*	4**
3	5.91 (d, 9.5)	5.91 (d, 9.5)	5.88 (d, 9.5)	6.21 (d, 9.5)	6.25 (d, 9.5)	6.35 (d, 9.5)
4	6.78 (d, 9.5)	6.95 overl	6.63 (d, 9.5)	7.85 (d, 9.5)	7.88 (d, 9.5)	7.72 (d, 9.5)
5	6.73 (d, 8.4)	6.95 overl	6.65 (d, 8.6)	7.49 (d, 8.6)	7.55(d, 8.5)	7.48 (d, 8.2)
6	6.60 (dd, 2.4, 8.4)	6.95 overl	6.56 (dd, 2.3, 8.6)	6.89 (dd, 1.8, 8.6)	6.96 overl	7.01 overl
8	6.62(d, 2.4)	6.95 overl	6.22(d, 1.8)	6.22(d, 1.8)	6.95 overl	7.02 overl
1′	4.21 (d, 6.5)	4.15 (d, 6.3)	1.12 overl	1.85 m	1.90 (dd, 4.6, 13.0), 1.99 m	1.82 m
2′	5.39 (t, 6.5)	5.40 (t, 6.5)	2.11 m, 2.17 m	2.12 m	2.35 (dd, 3.0, 12.5), 2.48 m	2.51 m
4′	1.95 m	1.94–2.16 m	–	–	2.64 m	2.63 m
5′	2.05 m	1.94–2.16 m	–	–	–	–
6′	5.09 (t, 6.9)	5.21 (t, 6.5)	1.70 m, 2.33 (bd, 18.6)	1.92 m 2.53 (bd, 13.5)	3.88 m	4.13 m
7′	–	–	0.97m, 1.30m,	1.28 m, 1.57 m	1.61 m, 1.99 m	1.82 m, 2.16 m
8′	1.62 s	1.94–2.16 m	1.46 m	1.28 m	1.99 m	1.96 m
9′	1.49 s	1.94–2.16 m	–	–	–	–
10′	1.48 s	5.17 (t, 6.5)	2.96 (dd, 4.5, 11.5)	3.03 (t, 8.0)	2.23 (bd, 9.7)	2.23 (bt, 7.3)
11′		–	3.42 (d, 8.3), 3.70 (d, 8.3)	3.75 (d, 8.5), 3.95 (d, 8.5)	3.88 m	3.85 (d, 9.0), 3.86 (d, 9.0)
12′		1.44 s	1.04 s	1.17 s	1.14 s	1.09 s
13′		1.55 s	1.45 s	1.46 s	1.12 overl	1.58 (d, 6.6)
14′		1.67 s	1.49 s	1.62 s	0.83 s	1.08 s
15′		1.55 s	0.69 (d, 6.7)	0.95 (d, 9.0)	1.12 overl	1.11 (d, 7.2)

*Data were measured in CD_3_OD. **Data were measured in Pyridine d_5_.

**Table 2:** ^13^C-NMR data (δ ppm) of compounds **1**–**4** in C_6_D_6_.

	1	2	3	3*	4*	4**
2	160.1	159.8	160.1	162.0	161.9	162.0
3	112.9	113.0	111.9	111.8	112.0	111.8
4	142.6	142.2	142.3	144.4	144.4	144.4
5	128.5	128.3	128.3	129.0	129.1	129.0
6	112.8	112.9	112.5	112.9	112.9	112.9
7	162.0	161.9	162.5	163.0	162.5	163.0
8	101.0	101.0	100.8	100.6	100.9	100.6
9	156.1	156.3	156.2	155.7	155.8	155.7
10	112.2	112.1	112.2	112.4	112.7	112.4
1′	65.1	65.0	22.0	21.8	23.1	23.4
2′	119.0	119.0	31.7	31.7	40.8	41.6
3′	141.2	141.2	172.6	176.8	214.2	211.7
4′	39.4	39.4	129.7	130.0	57.8	58.4
5′	26.2	26.1	125.9	125.5	47.2	47.5
6′	123.9	124.5	24.3	24.2	72.0	71.8
7′	131.4	135.2	32.0	31.6	35.8	36.8
8′	17.4	39.8	34.5	34.7	36.3	36.3
9′	25.5	26.8	40.5	40.5	39.1	39.3
10′	16.2	123.7	42.4	42.4	43.3	43.7
11′		130.9			75.4	75.8
12′		16.2			18.8	19.6
13′		15.8			9.4	11.1
14′		25.5			8.2	9.5
15′		17.4			14.5	15.5

*Data were measured in CD_3_OD. **Data were measured in Pyridine d_5_.

*Umbelliprenin* (**2**): White powder, UV λ_max_ MeOH: 223, 288, 325 nm, ^1^H and ^13^C NMR Tables 1, 2, Figures S13–S22, HRESIMS *m/z* [M + 1]^+^ m/z 367.2263 (calcd for C_24_H_30_O_3_ + H, 367.2273), [M + Na]^+^ m/z 389.2081 (calcd for C_24_H_30_O_3_ + Na, 389.2093) (Figure S23).

*Galbanic acid* (**3**): White matrix, UV λ_max_ MeOH: 224, 289, 324 nm, ^1^H and ^13^C NMR Tables 1, 2, Figures S24–S44, HRESIMS *m/z* [M-1]^+^ m/z 397.2016 (calcd for C_24_H_30_O_5_-H, 397.2015), [M + 1]^+^ m/z 399.2162 (calcd for C_24_H_30_O_5_ + H, 399.2171), [M + Na]^+^ m/z 421.1981 (calcd for C_24_H_30_O_5_ + Na, 421.1991) (Figure S45, S46).

*Kamolonol* (**4**): White amorphous powder, UV λ_max_ MeOH: 225, 285, 326 nm, ^1^H and ^13^C NMR Tables 1, 2, Figures S47–S60, HRESIMS *m/z* [M + 1]^+^ m/z 399.2166 (calcd for C_24_H_30_O_5_ + H, 399.2171), [M + Na]^+^ m/z 421.1986 (calcd for C_24_H_30_O_5_ + Na, 421.1991) (Figure S61) (Fig. 1).

**Fig. 1:**
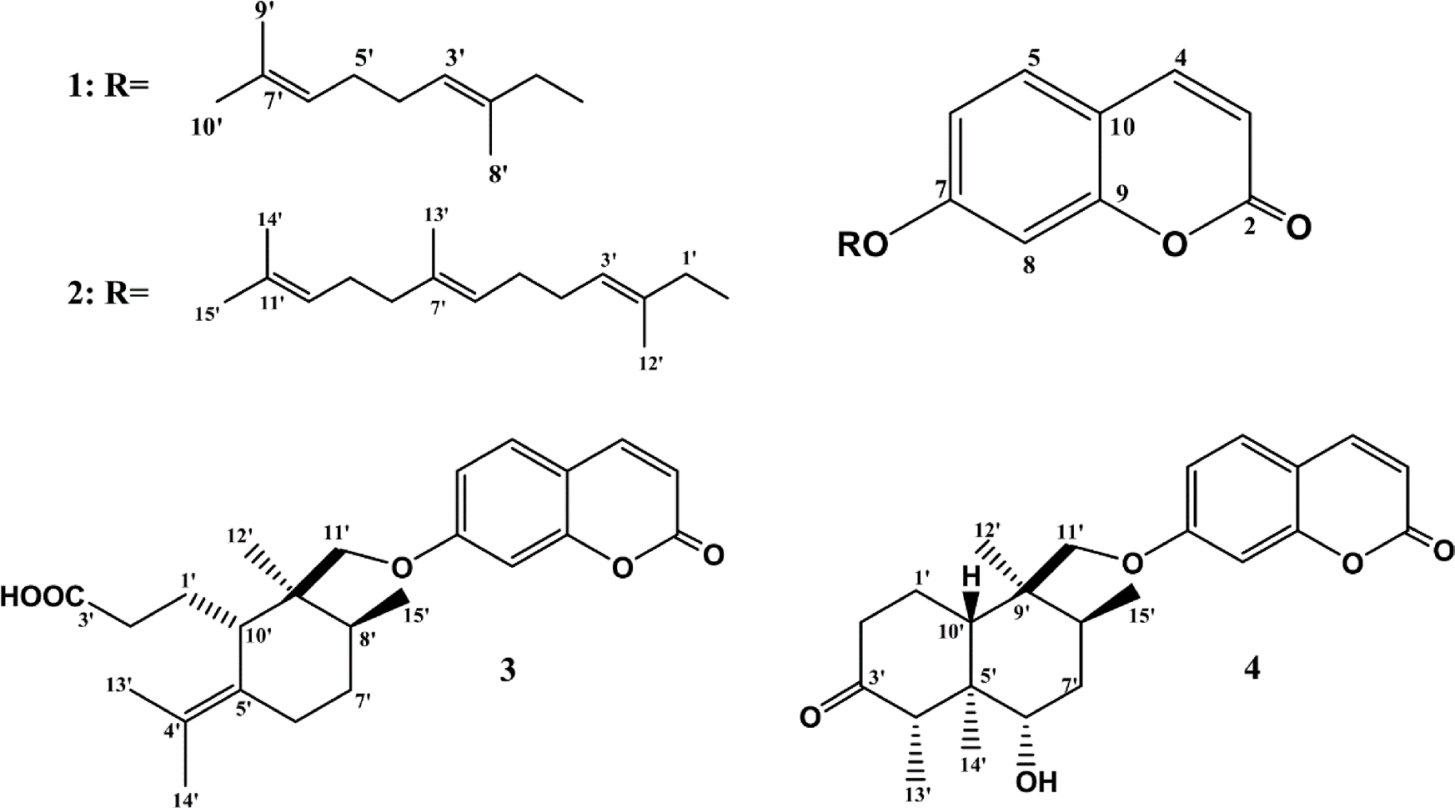
Structures of compounds **1**–**4**.

### *In vivo *anti-inflammatory testing

#### Carrageenan-induced acute paw edema model for extract and fractions

**Fig. 2:**
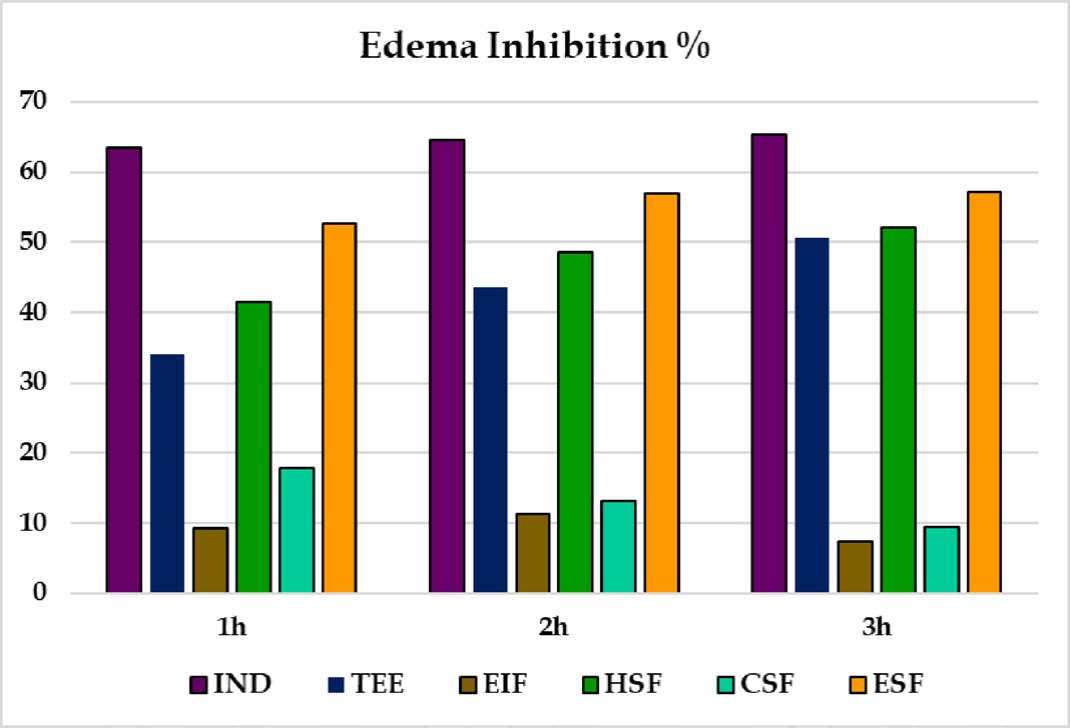
Effect of **TEE** and its fractions on the edema inhibition percentage of rat’s paw edema.

The anti-inflammatory *in vivo* testing of full and fractionated *Ferula assa-foetida* extracts is compared in Table 3 and shown in Fig. 2. In control group, after injection of carrageenan, there was a progressive paw swelling with an edema of 56.88%, 62.93% and 58.08% at the 1st, 2nd and 3rd hours following injection, respectively. Conversely, pretreatment with the total extract (**TEE**) resulted in a significant swelling reduction with edema ratios of 37.48%, 35.56%, and 28.71% ± 1.55%, at corresponding time points. It was similarly observed that the animals treated with the hexane fraction, (**HSF**) showed percentages reduction of 33.27, 32.35 and 27.79, while the ethyl acetate fraction (**ESF**) gave reduction percentage of 26.94, 27.11 and 24.90. Indomethacin (**IND**) resulted in 20.76%, 22.27%, and 20.16% edema rates as the reference drug. It is important to report that **ESF** showed almost the same anti-inflammatory profile as **IND**. Of all the samples, **ESF** 0.2g/kg administered showed the most significant inhibitory properties of carrageenan-induced paw edema, as evidenced by the inhibitory effects indicated by 52.63%, 56.63%, and 56.82% at 1, 2, and 3 h, respectively (Fig. 4). The chloroform fraction (**CSF)** and the ethanol-insoluble fraction  (**EIF)** made no apparent change in paw volume as compared with the untreated samples.

**Table 3:** Effect of **TEE** and its fractions on the edema rate percentage produced by carrageenan in rat’s paw.

Groups	Dose (mg/kg)	Edema rate (%)		
		1 h	2 h	3 h
CONT	00	56.88 ± 5.16#	62.93 ± 1.68#	58.08 ± 0.62#
IND	10	20.76 ± 1.13*	22.27 ± 0.96*	20.16 ± 0.93*
TEE	400	37.48 ± 3.66*#	35.56 ± 2.04*#	28.71 ± 1.55*#
EIF	400	51.58 ± 4.60#	55.75 ± 4.54#	53.73 ± 2.05#
HSF	200	33.27 ± 2.23*#	32.35 ± 2.03*#	27.79 ± 2.47*#
CSF	200	46.71 ± 1.58#	54.65 ± 2.11#	52.62 ± 1.07#
ESF	200	26.94 ± 1.08*	27.11 ± 0.80*	24.9 ± 0.69*

Values are presented as mean ± S.E of 6 animals for each group. *Statistically significant from control: *P* ≤ 0.05. #Statistically significant from IND: *P* ≤ 0.05.

### *In vitro* anti-inflammatory testing for pure compounds 1–4

#### Lipopolysaccharide-Induced Inflammation in RAW 264.7 Mouse Macrophages

**Fig. 3:**
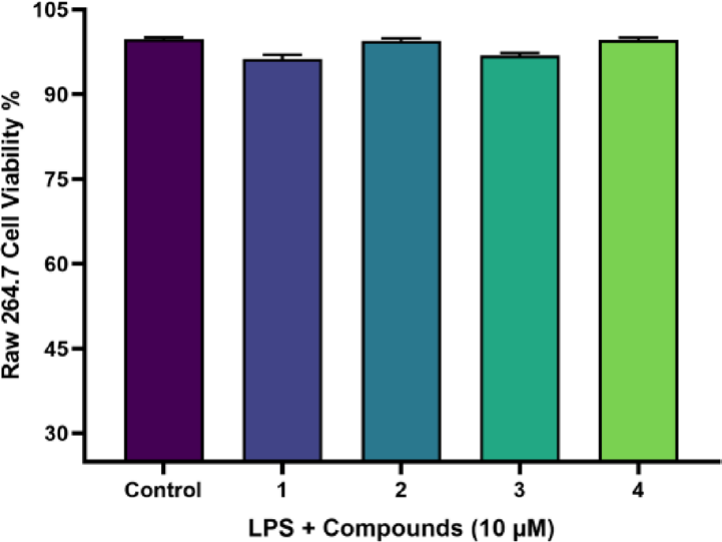
Cytotoxic assessment of compounds **1**–**4** (10 uM) in LPS-induced RAW 264.7 cells. The Cell viability was conducted using SRB assay. The results are presented as mean ± SD (n = 3). The statistical analysis revealed no significant differences (*P* ≤ 0.05) when compared to the LPS-induced cells.

The cytotoxicity of the isolated compounds on the production of nitric oxide (NO) inhibition as well as assessing their in vitro anti-inflammatory activities was determined via the sulforhodamine B (SRB)) assay on LPS-induced RAW 264.7 macrophages first. Concentration range in the test was 0.001 to 10 µM. The macrophage toxicity of the compounds tested was not significant at any of the doses tested with near full cell viability observed at the highest dose nearly 99% as shown in Fig. 3, compared with LPS-treated control. As a result, the following concentration ranges were chosen to perform the further inhibition experiments of nitric oxide.

### Nitric oxide inhibition test *in vitro*

**Fig. 4:**
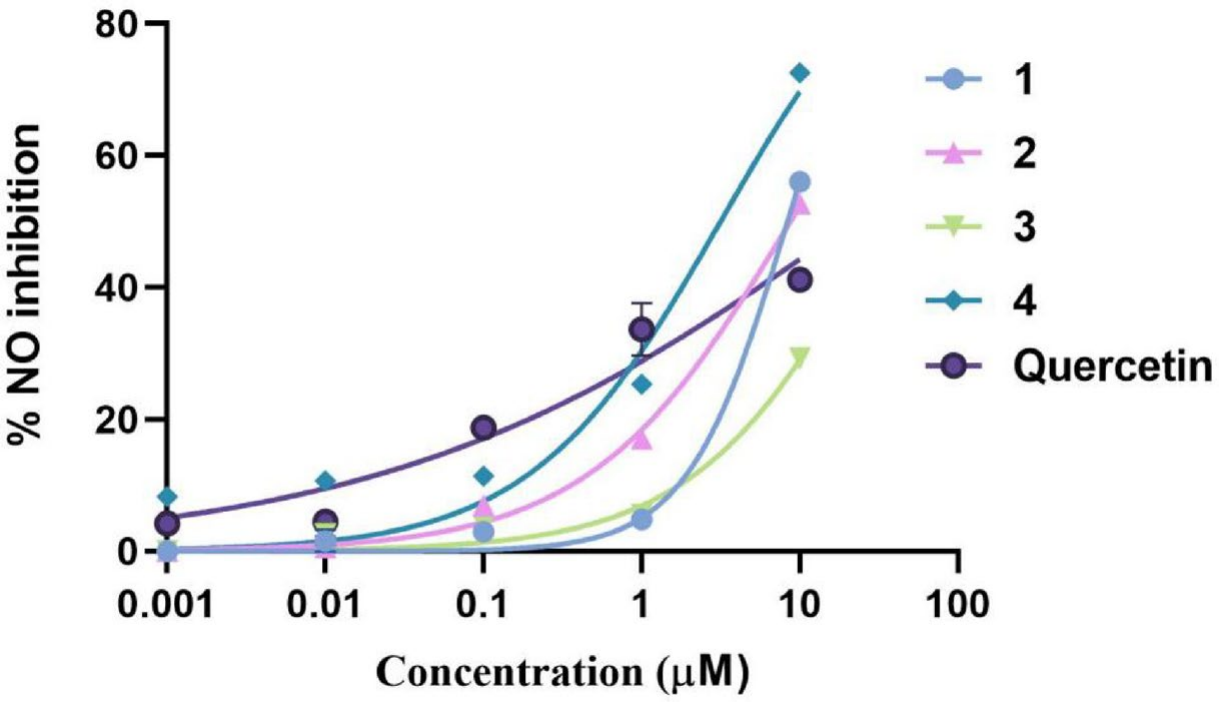
The inhibition rates of NO release from RAW 264.7 cells after treatment with **1**–**4** for 48 h.

As shown in Fig. 4, compound **4** was the most effective nitric oxide (NO) inhibitor, with an IC_50_ value of 3.165 ± 0.12 µM. Furthermore, compounds **1** and **2** also demonstrated potent NO inhibition activity, displaying nearly the same IC_50_ values of 8.388 ± 0.24 µM and 8.754 ± 0.17 µM, respectively. The three compounds **1**, **2** and **4** showed NO inhibition activity more than the positive control (quercetin with an IC_50_ value of 22.10 ± 0.11 µM). The least NO inhibitor in this series was compound **3** with an IC_50_ value of 33.59 ± 2.65 µM.

### Computational analysis of physicochemical and pharmacokinetic properties

Virtual analysis of physicochemical properties by the SwissADME database revealed that the most active compound **4** possesses the lowest lipid solubility and a high number of possible hydrogen-bonding (Table 4). While compounds **3** and **4** had similar number of hydrogen-bond acceptors and donors, their anti-inflammatory activity was dissimilar.Within the isolated compounds, only compound **4** was found to interact with the efflux pump P-glycoprotein (Pgp). Interactions with microsomal enzymes were variable with each compound having a different interaction profile than the other. Compound **2** interactions could not be determined by the SwissADME database.

**Table 4:** Relative anti-inflammatory activity (designated as NO inhibition), and computational analysis of physicochemical and pharmacokinetic properties.

Compound	NO inhibition (10 μM)	LogP	H-bond acceptors	H-bond donors	Pgp substrate	CYP1A2 inhibitor	CYP2C9 inhibitor	CYP2D6 inhibitor	CYP3A4 inhibitor
1	+ +	4.86	3	0	No	Yes	Yes	No	No
2	+ +	6.59	3	0	ND	ND	ND	ND	ND
3	+	5.43	5	1	No	No	Yes	No	Yes
4	+ + +	4.20	5	1	Yes	No	No	Yes	Yes

ND: could not be determined by SwissADME database.

The symbols (+ , +  + , +  + +) indicate different levels of activity: low, moderate, and high, respectively.

### Inflammation-related and compounds gene targets

A total of 16,356 genes were identified by the GeneCards database when using the search term “inflammation”. For this study, only the top 2,500 genes were considered to provide a stringent criterion for detection. The gene targets of compounds **1**–**4** were determined by the “SwissTargetPrediction” database. Collectively, compounds **1**–**4** had a total of 96 gene targets, some of which were exclusive to some compounds, while others were common (Table 5). Compound **3** had no predicted gene targets. Venn diagrams identified 39 gene targets overlapping between the top 2,500 inflammation genes, and the isolated compounds.

**Table 5:** The number of compounds’ gene targets predicted by the SwissTargetPredict database, and the number of target genes common with inflammation.

Compound	Gene targets	Overlapping with top 2500 inflammation-related genes
1	76	29
2	53	22
3	0	0
4	9	2
All	96	39

**Fig. 5:**
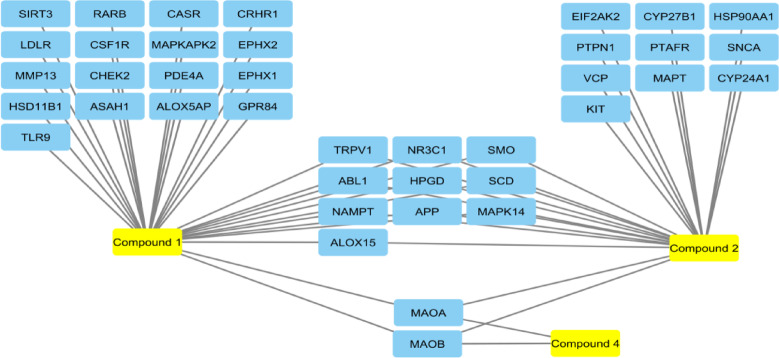
Network of the gene targets of compounds **1**, **2**, and **4** constructed by the Cytoscape software to highlight commonality of gene targets. Compound **3** had no predicted gene targets.

The Cytoscape software was utilized to construct a network highlighting the common and exclusive gene targets of each compound (Fig. 5). Interestingly, compounds **1**, **2**, and **4** had 2 common targets: monoamine oxidase-A (MAOA) and monoamine oxidase-B (MAOB).

### Protein–protein interactions

**Fig. 6:**
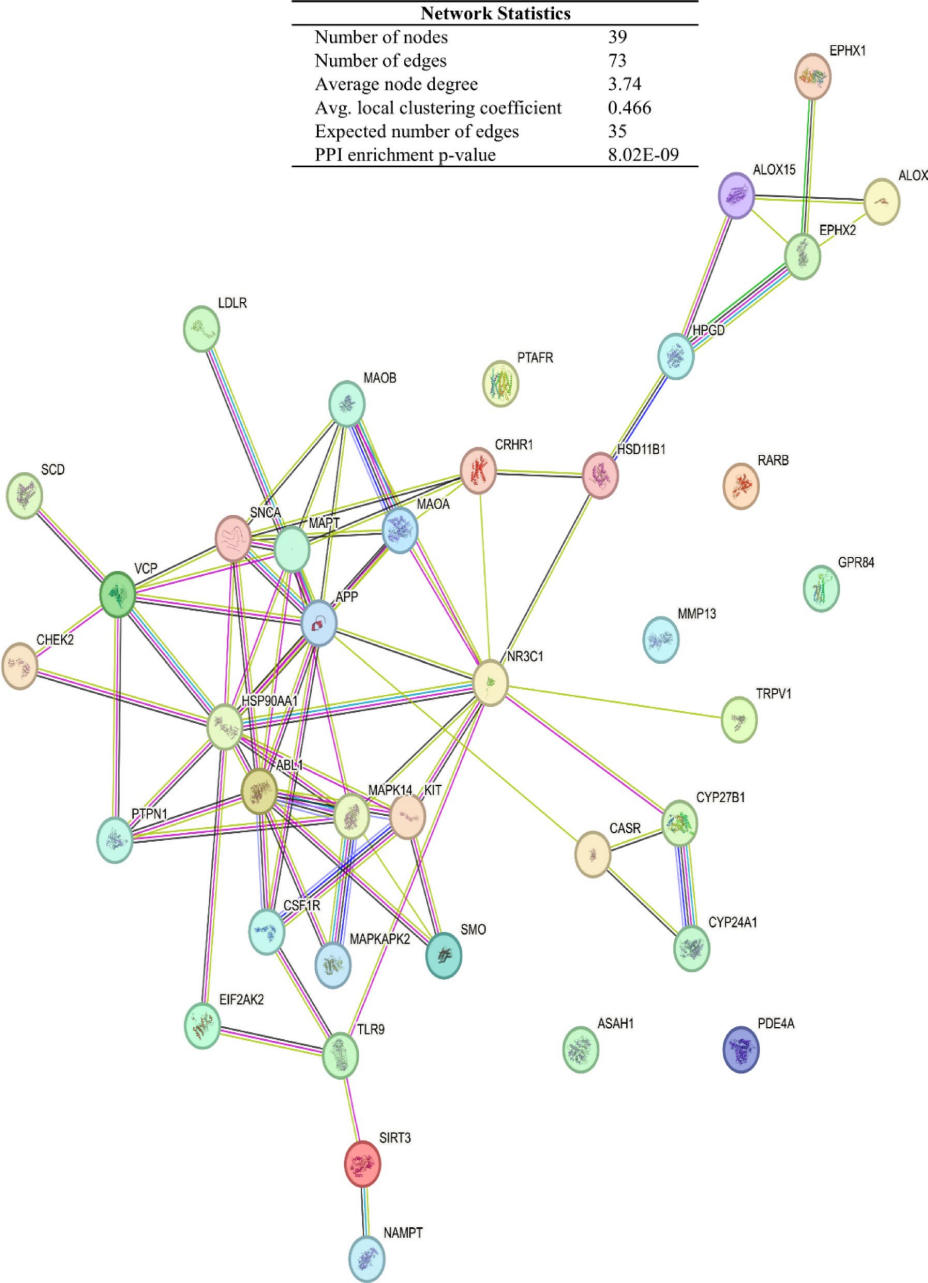
PPI network of the inflammation genes targeted by compounds **1**, **2**, and **4** constructed by the STRING database.

A network highlighting the protein–protein interactions (PPIs) of the 39 inflammation-related gene targets of compounds **1**, **2**, and **4** was constructed by the STRING database (Fig. 6). This aims to analyze known and predicted interactions between these genes, and has assigned an enrichment p-value of < 8.02e-9, where a small p-value indicates a significant interaction probability.

**Fig. 7:**
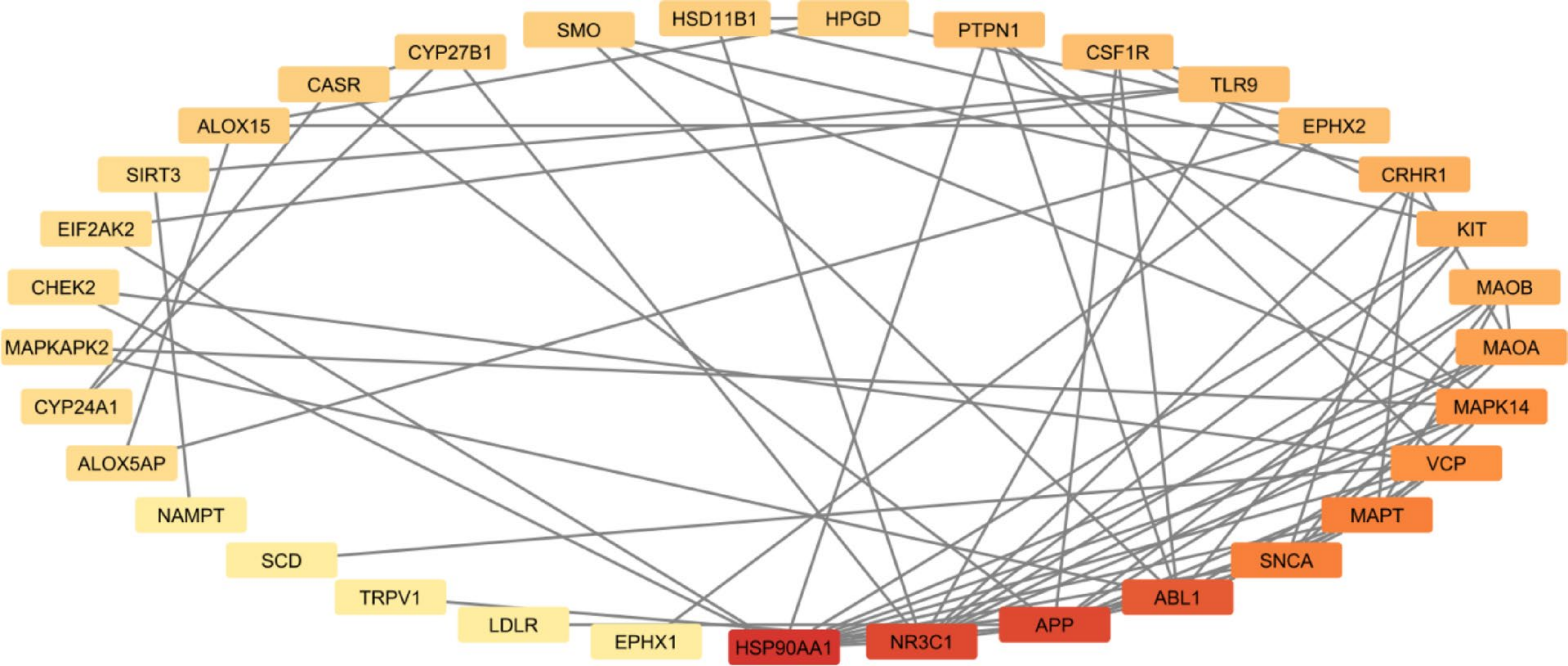
Cytoscape network highlighting the genes with the highest DC (red: highest, light yellow: lowest). Higher DC indicates a higher number of predicted and known PPIs.

The Cytoscape software was used to analyze and highlight the 39 gene targets identifying the core genes with the highest degree of centrality (DC) (Fig. 7).

The top 4 genes based on DC were found to be highly involved in immunogenic responses and inflammatory cytokines on several fronts (Table 6).

**Table 6:** Brief description of the genes with the highest DC.

Gene symbol	Description	DC
HSP90AA1	Heat shock protein 90 alpha family class A member 1. Chaperone protein that aids in folding other proteins. Found to promote inflammation and immune cell activation^18,19^	12
NR3C1	Nuclear receptor subfamily 3 group C member 1. Encodes glucocorticoid receptor, which is known to be involved in inflammatory responses and cellular proliferation^20,21^	11
APP	Amyloid beta precursor protein. Encodes the bases of amyloid plaques, which were found to have synergistic pro-inflammatory activity^22,23^	11
ABL1	ABL proto-oncogene 1. Encodes a protein kinase involved in cell division and adhesion. Also modulates innate immunity by regulating ubiquitination of other proteins^24^	10

### Enrichment analysis of the gene targets

**Fig. 8:**
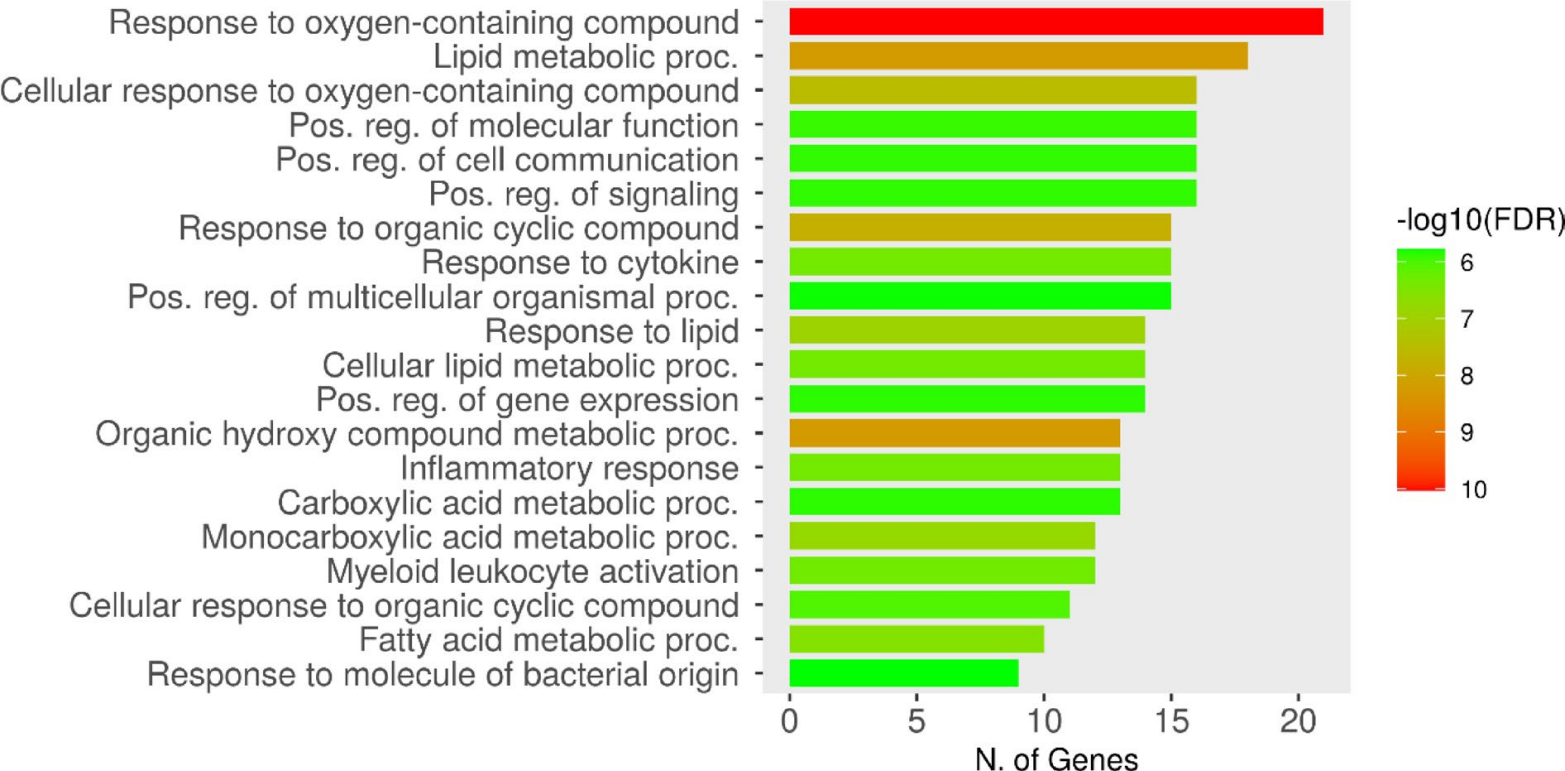
ShinyGO database analysis of the biological processes involving the inflammation-related gene targets of the isolated compounds **1**, **2**, and **4**. FDR: false discovery rate, smaller FDR indicates high statistical significance.

Analysis of the 39 inflammation genes identified previously was performed by the ShinyGO database. Analysis of the “biological process” revealed the targeted genes to be involved in several biological processes, including the cellular response to several molecular structural aspects such as oxygen-containing compounds, organic cyclic compounds, cytokines in general, lipids, organic hydroxy compounds, and carboxylic acids (Fig. 8).

### Molecular docking analysis of target proteins with compounds

For the molecular docking analysis, the common target genes MAOA and MAOB were selected after analyzing the Cytoscape software network due to these genes being common targets to compounds **1**, **2**, and **4**. The docking protocol was initially validated by redocking the co-crystalized ligands namely, Clorgyline and CS-0379511 against their target proteins, MAOA and MAOB, respectively. Both ligands adapted similar binding conformations to crystalized once with high binding energy scores, − 12.90 and − 12.60 kcal/Mol respectively (Table 7). For MAOA, compound **4** showed the highest binding energy score of − 12.00 kcal/mol, while compound **2** showed the highest score (-11.40 kcal/mol) with MAOB enzyme. Remarkably, compound **3** showed low energy scores against both proteins supporting the previous prediction of non-interaction with MAOA and MAOB.

**Table 7:** Structures, binding energy and interaction residues of the isolated compounds compared to known ligands of both MAOA and MAOB. Residues involved in hydrogen bond interactions with the compounds are bolded.

Ligand ID	Chemical structure	Binding energy (kcal/mol)		Interaction residues	
		MAOA	MAOB	MAOA	MAOB
**Clorgyline**		− 12.90	–	–	–
**CS-0379511**		–	− 12.50	–	–
**Compound 1**		− 9.70	− 10.30	Ile23, Phe352 Tyr407, **Thr435, Cys434**, Ala448	Phe103, His115, Trp119, Leu164, Leu167, Leu171, Cys172, Ile199, Phe168, Tyr220, Ile316
**Compound 2**		− 10.20	− 11.40	Ile23,Arg51, Tyr59, Ile180, Ile335, Phe352, Cys406, tyr407, Tyr444, Met445, Ala448	Phe103, Phe118, Try119, Phe168, Leu171, Cys172, Ile199, Try398, **Tyr435**
**Compound 3**		− 10.80	− 8.90	Tyr69, Ile180, Phe208, **Ser209**, Cys323, Ile335, Leu337, Phe352, Tyr407, **Met445**	Phe103, Pro104,His115, Leu167, Leu171,**Thr195,** Ile198, Ile199, Gln206, Try320 ,Ile316, **Try435**
**Compound 4**		− 12.00	− 9.10	Tyr69, Gly67, Ile180, Phe208, **Ser209**, Ile335, Leu337, Phe352, Tyr407, Tyr444	Pro104, His115, Ile164, Ile167, Leu171, Ile199, Gln206, Ile316, Try320, **Tyr435**

**Fig. 9:**
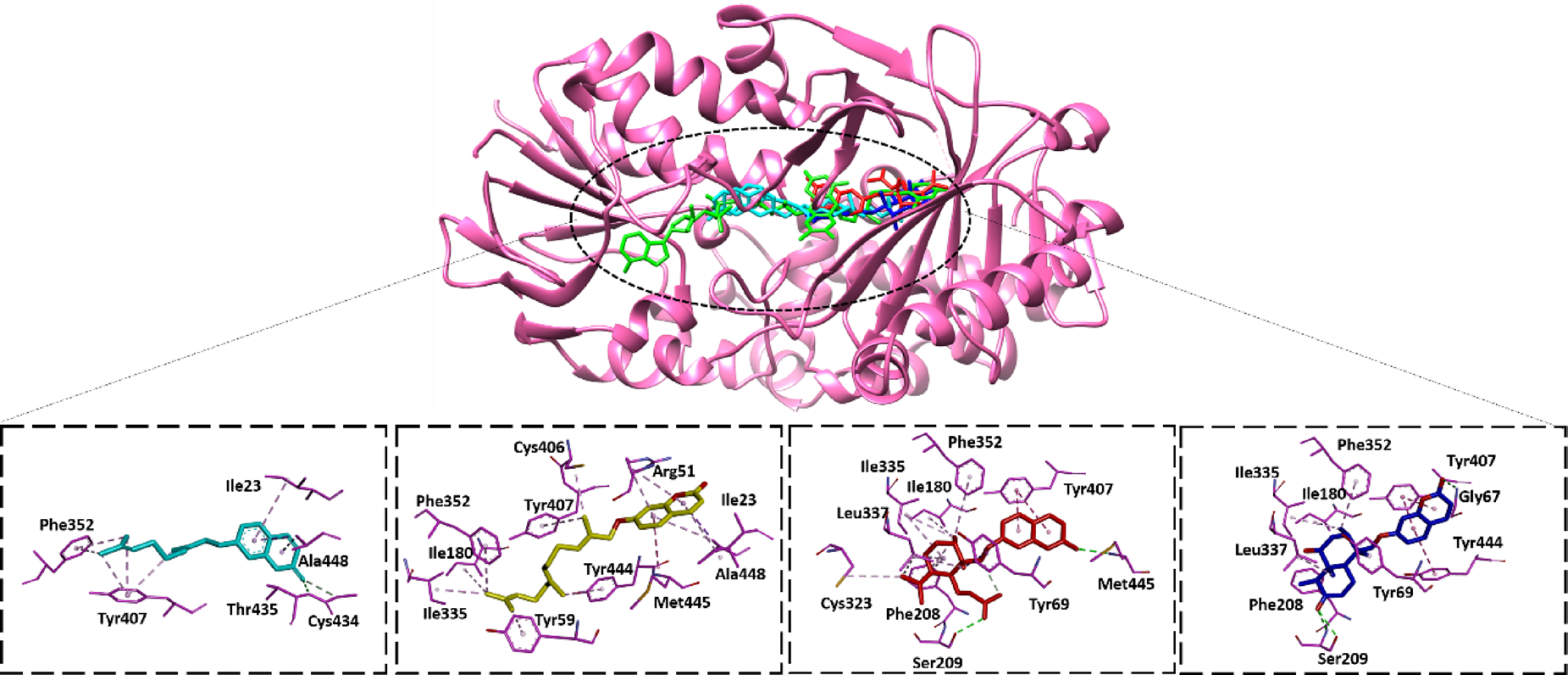
The docked complexes of compounds with MAOA protein (PDB ID: 2BXR). Compound **1**: Cyan, Compound **2**: Yellow, Compound **3**: Red, Compound **4**: Blue.

**Fig. 10:**
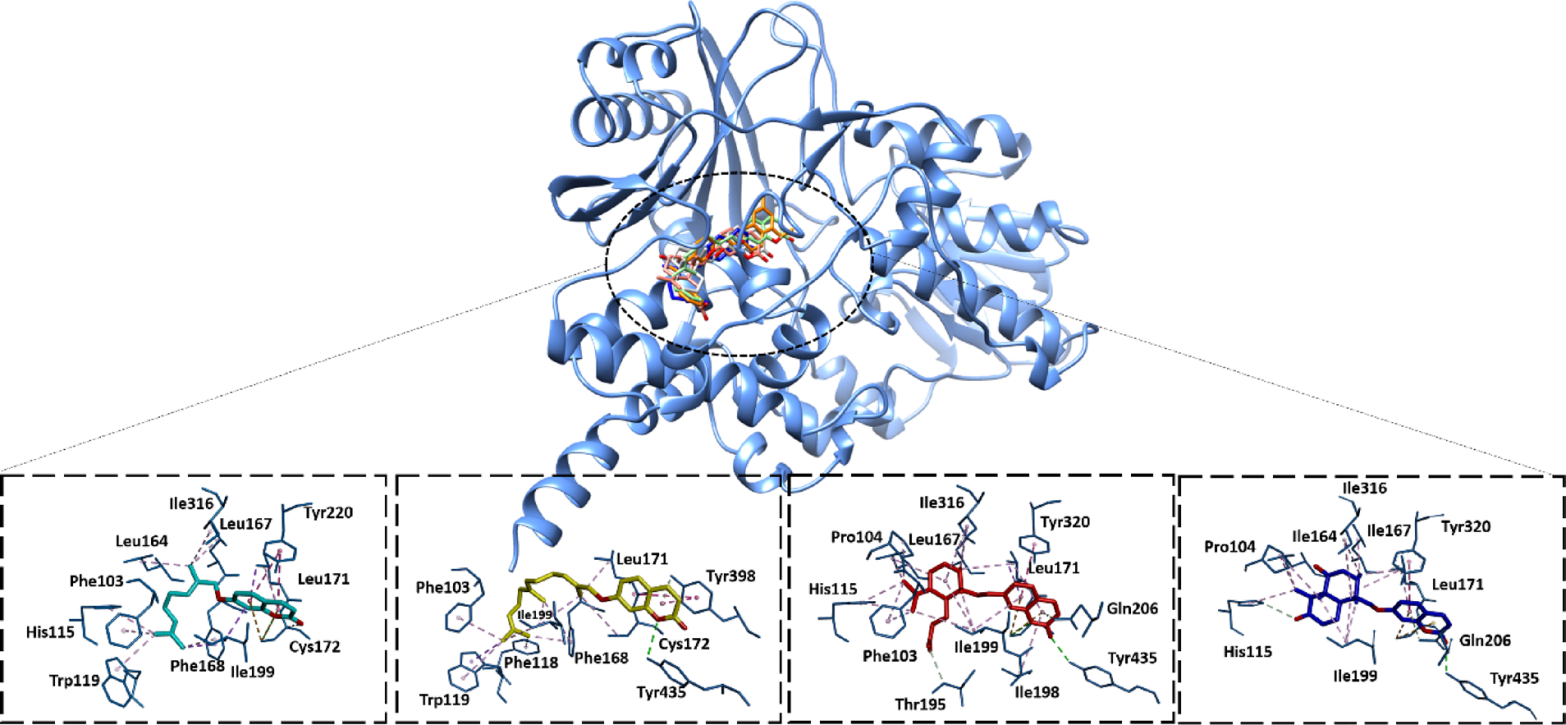
The docked complexes of compounds with MAOB protein (PDB ID: 7P4F). Compound **1**: Cyan, Compound **2**: Yellow, Compound **3**: Red, Compound **4**: Blue.

The compounds interacted with the active sites of MAOA and MAOB via multiple interactions forces (Figs. 9 and 10). The interaction residues shown in Table 7. For compound **4**, which is the most active one, it showed better binding with MAOA compared with MAOB. For MAOA, compound **4** involved in hydrogen binding with Ser209 with the carbonyl of hydronaphthalenone and multiple hydrophobic interactions such as Gly67, Tyr69, Ile180, Phe208, Ile335, Leu337, Phe352, Tyr407, and Tyr444 (Fig. 9). Similarly, compound **4** interacted with MAOB through formation of hydrogen bond with Tyr435 the carbonyl of hydronaphthalenone and other hydrophobic bonds with Pro104, His115, Ile164, Leu171, Ile199, Gln206, Ile316 and Tyr320 (Fig. 10).

### Molecular dynamic simulation analysis of compound 4 with MAOA and MAOB

**Fig. 11:**
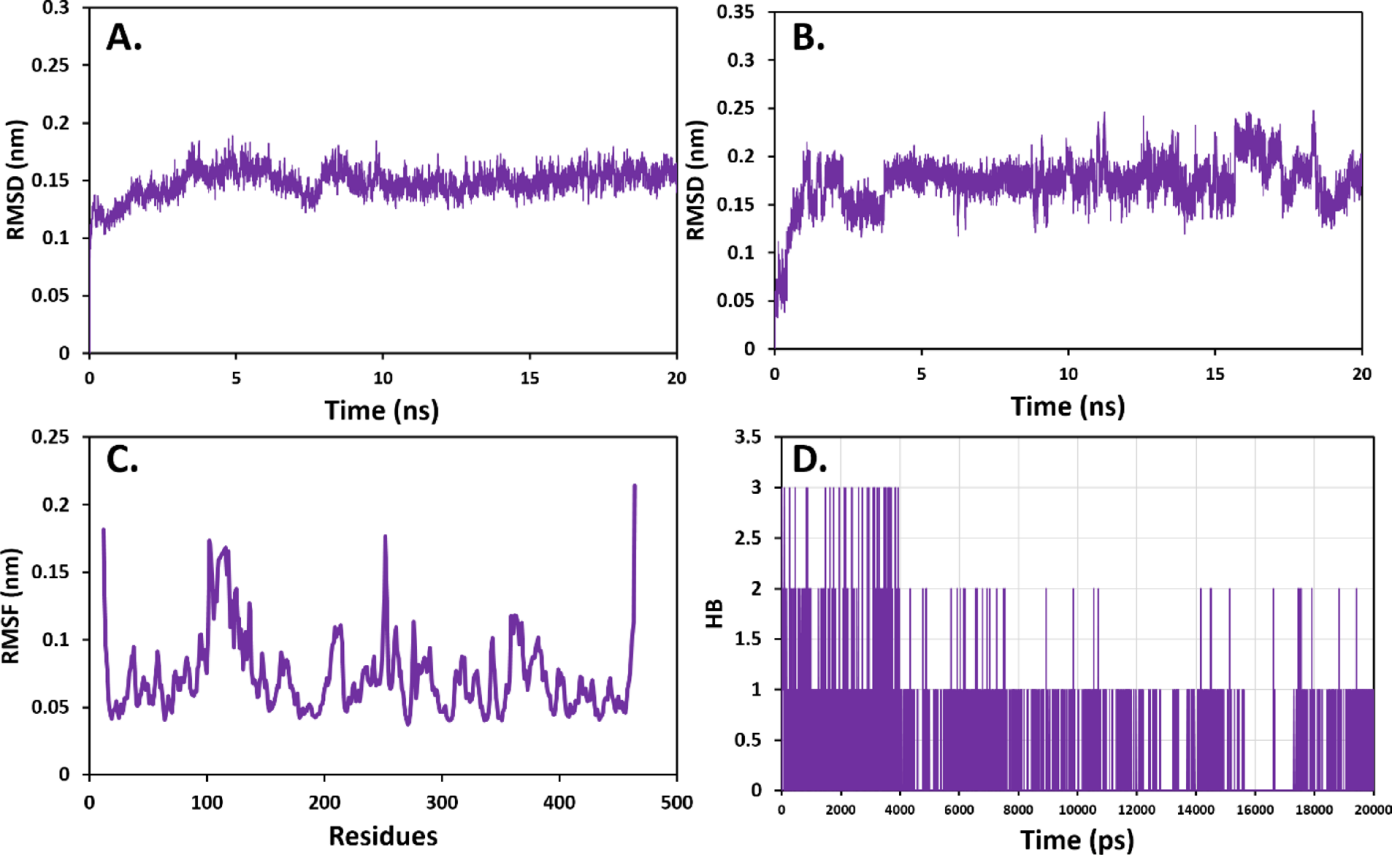
MD simulation analysis of compound **4** binding to active site of MAOA (PDB ID: 2bxr). (**A**) Protein backbone RMSD, (**B**) Ligand RMSD, (**C**) Protein backbone RMSF, (**D**) Number of hydrogen bonds.

The most active compound, compound **4**, was subjected to 20 ns molecular dynamic (MD) simulation to determine its interaction behavior with both proteins, MAOA and MAOB, in a manner mimicking physiological conditions. For the binding of compound **4** with MAOA, both protein backbone and ligand root mean square deviation (RMSD) showed a behavior without major fluctuations over the simulation-time suggesting a stable binding condition (Fig. 11A,B). The root means square fluctuation (RMSF) analysis showed that in addition to the high fluctuations in the incompatible residues located in the N-terminal (amino acids up to No. 12) as well as in the C-terminal (459–463), some fluctuation was seen in loop regions around residue 102–125 and 251–252 (Fig. 11C). The analysis of intermolecular hydrogen bond formation between compound **4** and MAOA indicated that this compound can form a maximum of 3 bonds. Hence, the formation of hydrogen bonds contributes significantly to the binding energy of interaction (Fig. 11D).

**Fig. 12:**
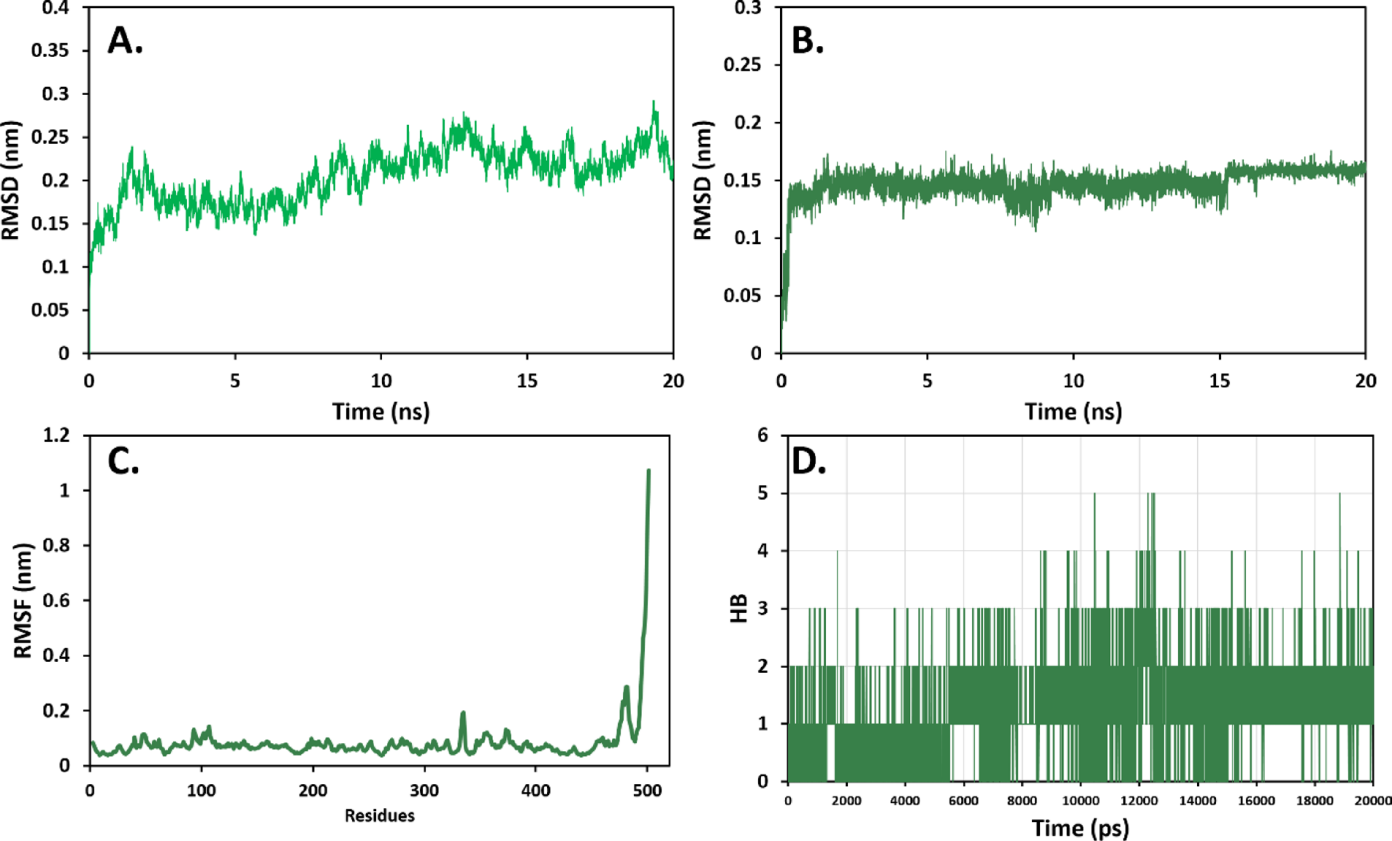
MD simulation analysis of compound **4** binding to active site of MAOB (PDB ID: 7p4f.). (**A**) Protein backbone RMSD, (**B**) Ligand RMSD, (**C**) Protein backbone RMSF, (**D**) Number of hydrogen bonds.

Similarly, it can be seen in Fig. 12A,B that the interaction behavior of compound **4** with MAOB follows the same fluctuation pattern as shown with MAOA. No major disrupted fluctuations with backbone RMSD between 0.15–0.25 nm and ligand RMSD being stable around 0.15 nm. In addition, The RMSF analysis showed that compound **4** binding residues (Pro104, His115, Ile164, Leu171, Ile199, Gln206, Ile316, Tyr320, and Tyr435) have very low fluctuations behavior. The significant fluctuations can be seen in the c-terminal region (490–501) (Fig. 12C). The analysis of intermolecular hydrogen bond formation between compound **4** and MAOB showed that there can be up to 5 stable hydrogen bonds between the ligand and protein (Fig. 12D).

## Discussion

Inflammation is an inherent natural physiological defense mechanism through which the organism responds to tissue injury or infection and the main goal of which is the restoration of both structural and functional integrity^1^. Although steroidal and non-steroidal anti-inflammatory drugs (NSAIDs) are common in the clinical world to treat inflammation-related conditions, the drug delivery process is often surrounded by unwanted side effects and its possible complications^25^. Plant-based natural agents, especially secondary metabolites of medicinal plants, have therefore gained attraction as alternate sources of anti-inflammatory. The carrageenan-induced paw edema model in rodents has been found as one of the most frequently used experimental methods in studying the potential response of an experimental animal by administering carrageenans as an inflammatory agent under laboratory conditions. It is a common model of acute inflammation, during which there is infiltration of polymorphonuclear neutrophils with an increase in prostaglandin production^26^. Inflammatory process caused by carrageenan involves neutrophilic leukocyte recruitment and the production of prostaglandins which further instigate the production of histamine, serotonin, bradykinin, among other inflammatory mediators^27^. The volume increase of the paws is normally used to indicate the level of inflammation in experimental assessment.

This experiment aimed to study the anti-inflammatory properties of the total extract of *Ferula assa-foetida* **(TEE)** and its fractions which are derived through the carrageenan-induced paw edema test in rats. There was a significant inhibition of paw swelling in the **TEE** treated group (34.10%, 43.12%, and 50.21%) 1 h, 2 h, and 3 h following induction of inflammation respectively. The hexane fraction **(HSF)** exhibited a significant activity as well with inhibition values of 41.65%, 53.33%, and 54.44% over the same periods of time. In the previous pharmacodynamic studies, it was reported that when administered orally, the ethyl acetate fraction **(ESF)** used at 200 mg/kg demonstrated greater anti-inflammatory effect, than the reference NSAIDs indomethacin (IND) at 10 mg/kg^28^. IND is quite known as having an analgesic, antipyretic, and inflammation inhibitory effect.  Interestingly, the *in vivo* anti-inflammatory effect of **ESF**  is comparable with that produced by IND suggesting the therapeutic potential of the tested extract. Conversely, the insoluble residue fraction **(EIF)** and the chloroform fraction **(CSF)** did not induce any anti-inflammatory effects under the same experimental conditions.

Phytochemical study of the most active **HSF** and **ESF** resulted in the isolation of four coumarins coupled with various terpene moieties **1–4**. The four compounds showed five signals in the aromatic region assigned for H-3, H-4, H-5, H-6 and H-8 (Table 2) with their correlated carbons (Table 3) were diagnostic for 7-oxygenated coumarin skeleton common in *Ferula* species^15–17^. Compound **1** showed in the HRESIMS an [M-H]^+^ at 297.1483 m/z in the negative mode and [M + H]^+^ at 299.1640 m/z for the molecular formula C_19_H_22_O_3_ (Figures S11, S12). The additional 10 carbon signals over the coumarin skeleton were sorted by DEPT135 experiment (Figure S8) into CH_3_ X 3, CH_2_ X 3, CH X 2 and 2 quaternary carbons. The molecular formula indicated 9 degrees of unsaturation. As the coumarin skeleton fulfill 7 unsaturation degrees, the monoterpene moiety was left with only 2 degrees of unsaturation. Two double bonds were observed in the ^1^HNMR and ^13^CNMR of 1 at δ_H_ 5.39 (t, *J* = 6.5 Hz), δ_C_ 119.0, 141.2 ppm and δ_H_ 5.09 (t, *J* = 6.9 Hz), δ_C_ 123.9, 131.4 ppm and assigned for C-2′ and C-6′ respectively (Tables 2, 3, Figures S1–S8). These features were diagnostic for acyclic monoterpene moiety coupled to the coumarin skeleton. The data of **1** were identical with those reported for auraptene^29,30^. Auraptene was reported to be effective for the management of inflammatory disorders, dysentery, wounds, scars, keloids, and pain^31^.

^13^CNMR and DEPT135 of **2** (Table 3, Figures S16–S20) displayed extra 5 carbon signals over **1**. The three degrees of unsaturation left for the terpenoid moiety were fulfilled by 3 double bonds. Signals at δ_H_ 5.40 (t, *J* = 6.5 Hz), δ_C_ 119.0, 141.2 ppm, δ_H_ 5.21 (t, *J* = 6.5 Hz), δ_C_ 124.5, 135.2 ppm and δ_H_ 5.17 (t, *J* = 6.5 Hz), δ_C_ 123.7, 130.9 ppm were assigned for C-2′, C-6′ and C-10′ double bonds, respectively in an acyclic sesquiterpene structure (Tables 2, 3, Figures S13–S20). HRESIMS showed [M + H]^+^ at 367.2263 m/z and [M + Na]^+^ at 367.2263 m/z (Figure S23) for the molecular formula C_24_H_30_O_3_. The data of **2** showed close similarity with those reported for umbelliprenin^32^. Umbelliprenin is reported to possess cancer chemopreventive, anti-bacterial, anti-protozoal, anti-fungal, anti-inflammatory, neuroprotective, and antioxidant^33^.

Compound **3** showed in the HRESIMS an [M-H]^+^ at 397.2016 m/z in the negative mode and [M + H]^+^ at 399.2162 m/z and [M + Na]^+^ 421.1981 in the positive mode m/z for the molecular formula C_24_H_30_O_5_ (Figures S45, S46). In the ^13^CMR in addition to the coumarin skeleton, 15 carbon resonances were observed and categorized by DEPT135 experiment into CH_3_ X 4, CH_2_ X 5, CH X 2 and 4 quaternary carbons (Table 3, Figures S27–S30, S37–S39). The molecular formula revealed 10 degrees of unsaturation’s three of them were for the terpenoid moiety. Compound 3 showed two extra oxygen atoms over **2**. These two oxygen atoms were assigned for a carboxylic group at δ_C_ 172.6 ppm. Two olefinic carbons at δ_C_ 129.7 and 125.9 ppm constituted one double bond. The left unsaturation degree indicated a ring structure in the terpene part. Spectral data of **3** expressed close similarity with galbanic acid literature data^34^. Among the reported biological activity of galbanic acid are the anticancer, cancer chemopreventive, anticoagulant, hepatoprotective, antiviral, acetylcholinesterase inhibitory and antileishmanial activities^35^.

The HRESIMS of **4** was identical with **3** showing an [M + H]^+^ at 399.2165 and [M + Na]^+^ 421.1982 m/z (Figure S61). The ^13^CNMR spectra showed carbonyl signal at δ_C_ 214.2 ppm diagnostic for a ketone function 9. Another oxygen was involved in the formation of secondary alcohol function at δ_C_ 72.6 ppm. There was no indication of the presence of double bonds in the terpenoid part and consequently, there must be two ring structures (Table 3, Figures S49–S51, S57-S60). Comparison with literature data enables the identification of **4** as the sesquiterpene coumarin kamolonol^36^. Cytotoxicity antimicrobial and antioxidant activity were reported for Kamolonol acetate^37^. Kamolonol showed antibacterial activity against *Heliobacter pylori* and *Staphylococcus aureus*^36^.

Nitric oxide (NO) is regarded as a central pro-inflammatory factor in the development and progressive evolution of various inflammatory illnesses. High levels of NO have been observed in instances of increased severity of disease, and this shows that it is a good parameter in the treatment of inflammation by way of monitoring^38,39^. Prevention of excessive formation of NO is thus a logical mode of treatment of inflammatory diseases. The current in vitro experiment evaluated the inhibitory activity of the isolated components on NO production in the case of lipopolysaccharide (LPS)-activated macrophages as alternative to the in vivo testing that require more quantities from the test materials. The in vitro NO inhibition assay demonstrated that compounds **1, 2,** and **4** showed higher NO inhibition activity compared to the positive control quercetin (IC₅₀ = 22.10 ± 0.11 µM). In contrast, compound **3** exhibited the weakest inhibition, with an IC₅₀ value of 33.59 ± 2.65 µM.

The physiochemical behavior of these compounds **3** and **4** was found to have almost the same parameters (Table 4), but the anti-inflammatory activity of the two compounds varied decisively. This discrepancy could be attributed to the carboxylic acid moiety in compound **3 (**galbanic acid), which may ionize at physiological pH, compromising membrane permeability and intracellular uptake. This hypothesis aligns with previous reports on methyl galbanate, where esterification enhances bioavailability by preventing ionization^35^. Compounds **1** and **2** lack the hydroxyl and carbonyl functions in compound **4** enable better binding of the latter at the molecular targets. Compound **2** had no prediction of pharmacokinetic interactions. This can be attributed to the lack of structural similarity with known ligands currently within the SwissADME database.

The SwissTargetPrediction database failed to detect gene targets for compound **3** (Table 5). The remaining isolated compounds **1**, **2**, and **4** had common targets of MAOA and MAOB (Fig. 5), both of which are implicated in immunity and inflammatory responses^40–42^. Targeting these genes could mediate the observed anti-inflammatory properties.

Analysis of PPIs revealed a high degree of interaction revolving around inflammation and immunity-related genes (Table 6). This is consistent with the observed anti-inflammatory properties. Even though the common gene targets (MAOA and MAOB) were not the top genes in terms of interactions, they still had a relatively high DC (Fig. 7).

Biological processes in gene ontology describe and categorize overarching cellular events rather than specific pathways or biological components, in this study, compounds **1**, **2**, and **4** were predicted to interact with a plethora of biological targets with the common trait of being responsive to oxygen-containing compounds, regardless of whether these compounds are exogenous or endogenous. Enrichment analysis of the 39 genes targeted by compounds **1**, **2**, and **4** also suggests involvement with a variety of cellular responses affecting cell activity, cellular movement, secretions, and metabolic states. This is due to targeting several receptor pathways and binding of many enzymes including kinases (Fig. 8). This encompasses a wide variety of cellular functions, not excluding inflammatory responses^43^.

Molecular docking revealed that all isolated compounds are localized within the pharmacophores of both MAOA and MAOB in a manner like known controls (Figs. 9 and 10). Compound **4** had highest binding affinity (− 12.00 and − 9.10 kcal/mol against MAOA and MAOB, respectively), a characteristic that was associated with its maximum anti-inflammatory activity compared to compound **3** that was characterized by the weakest interaction. The docking findings were consistent with the molecular dynamics’ simulations of the compound **4** with MAO-A and MAO-B in which the established ligand–protein interaction was stable over the simulation time, as evidenced by the stable RMSD and RMSF patterns and consistent hydrogen bonding. The above findings (Figs. 11 and 12), together argue that the compound could be stable and suitable in physiological conditions.

## Conclusion

This study shows that *Ferula assa-foetida* has anti-inflammatory properties, especially in the EtOAc fraction, which supports its usage in ethnomedicine. The most promising pharmacological characteristics of the isolated sesquiterpene coumarins were demonstrated by kamolonol (compound 4), which exhibited substantial affinity for monoamine oxidase A (MAOA) and B (MAOB), superior nitric oxide (NO) inhibition, and sustained molecular interactions in physiologically simulated settings. The anti-inflammatory effect of **4** is reported for the first time. These results establish compound 4 as a viable candidate for additional drug development initiatives in addition to identifying MAOA and MAOB as anti-inflammatory targets. In vivo models of chronic inflammation, thorough pharmacokinetic characterization, and synergistic studies with established anti-inflammatory drugs.Such investigations could further affirm the therapeutic relevance of compound 4 and support its progression toward clinical application.

## Methods

### General

The tools and materials used in the process of this study have been sufficiently outlined in the previous studies^44^.

### Asafoetida oleo-gum-resin

In 2023, oleo-gum-resin of *Ferula assa-foetida* (asafoetida) was obtained in one of the Riyadh markets, Saudi Arabia.

### Extraction and isolation

An amount of 1000 g oleo-gum-resins underwent exhaustive extraction using alcoholic content of 95%. Aggregation of the combination of ethanol extracts obtained yielded 600.45 g of the total ethanol extract (**TEE**). Ethanol-insoluble fraction (**EIF**) had the mass of 399.55 g as is the case with the remaining quantity of the initial starting material. **TEE** (28 g) was dissolved in 300 mL of 40% aqueous ethanol, fractionated by successive liquid–liquid extraction using an aliquot part. Hexane (3 × 400 mL) partitioning gave 13.45 g of the hexane-soluble fraction (**HSF**). Extraction with chloroform (CHCl_3_) (4 × 400 mL) yielded 3.60 g of the CHCl_3_-soluble fraction (**CSF**) and ethyl acetate (EtOAc) ( 2 × 400 mL) gave rise to 5.00 g of the EtOAc-soluble fraction (**ESF**).

The **HSF** fraction (10 g) was used as the sample under the column chromatography (gravity) using silica gel (250 g) over packed gravity column (150 × 5 cm i.ds.). Hexane was used to elute the column followed by hexane/EtOAc gradient. Fractions of 200 ml were taken and attached through thin layer chromatography (TLC). Fractions exhibiting the same TLC patterns were pooled into 4 fractions, namely **HSF**-A to and **HSF**-D. Reversed-phase RP-18 medium-pressure liquid chromatography (MPLC) (45 × 1 cm i.d.) in a water/methanol gradient was used to purify the fraction of HSF-B (1.38 g) sediment that remains purified after chromatography through the silica gel to a greater extent (fraction HSF-B). A 70% methanol elution provided 66 mg of compound **1.**

Fractions eluted with 75% MeOH (72 mg) were further purified via preparative TLC (CHCl₃/MeOH, 9.5:0.5), affording 34 mg of compound **2.**

Fraction **D** (0.78 g), eluted with hexane/EtOAc (1:1), was purified using a flash chromatography column (45 cm × 1 cm i.d., 30 g silica gel) with CHCl₃ followed by CHCl₃/MeOH gradient. Fractions eluted with CHCl₃/MeOH (95:5) afforded 75 mg of compound **3.**

Column chromatography on silica gel (75 × 3 cm i.d., 150 g) was applied to a 4.34 g sample of the **ESF** fraction. EtOAc, followed by ethyl acetate (EtOAc)-methanol (MeOH) mixtures was used in a gradients system. Fractions eluted with 5% MeOH in EtOAc (252 mg) were purified further on a RP18 flash column eluted with 70% MeOH/30% H_2_O to afford 35 mg of **4**. The more polar fractions provided 200 g of glucose.

### Experimental animals

This animal experiment was conducted in accordance with the ARRIVE 2.0 guidelines for reporting animal research^45^.

Forty-two male Wistar rats of 7-month-old and weighing about 180–200 g, were obtained from the Laboratory Animal Unit (LBU) of Prince Sattam bin Abdulaziz University, Al Kharj, Saudi Arabia. The animals had a one-week acclimatization prior to the beginning of the experiment; during the acclimatization period animals were kept in ventilated cages (Rat IVC Blue Line, Techniplast, Buguggiate VA, Italy) which had sawdust bedding with moisture-absorbing characteristics. Housing was in constant condition of controlled temperature of 25 ± 1°C, 12:12 h of light dark cycle and free access to standard laboratory diet and filtered water were offered in stainless steel drinking bottles. Fuel cleaning was also a common practice and allowed the bedding to be changed on a regular basis, therefore sanitary conditions in housing were to be maintained. Animal handling during the work was carried out following the ARRIVE guidelines.

### Animal ethics statement

The animal experiments obeyed all the ethical considerations of laboratory animal care spelled out by the Scientific Research Ethics Committee at Prince Sattam bin Abdulaziz University. Approval was given by the issuance of authorization number SCBR-319/2024 and the procedures undertaken were in conformity to the national guidelines when treating animals with humane treatment and welfare of animals.

### Carrageenan-induced acute paw edema model

The anti-inflammatory effects of the crude extract (**TEE**) and four major fractions (**EIF**, **HSF**, **CSF**, **ESF**) of *Ferula assa-foetida* were evaluated using the carrageenan-induced paw edema model in adult male Wistar rats (7 months old, 180–200 g). Forty-two rats were randomly divided into seven groups (6 rats each). Indomethacin (10 mg/kg, oral) served as the positive control, while the negative control received saline (2 mL/kg). Treatments included oral administration of **TEE** or **EIF** (400 mg/kg) and **HSF**, **CSF**, or **ESF** (200 mg/kg). One hour post-treatment, inflammation was induced by injecting 0.1 mL of 1% λ-carrageenan into the right hind paw supplanter region. Paw volumes were recorded pre-injection and at 1, 2, and 3 h post-injection using a digital plethysmometer. Edema volume and percentage inhibition were calculated following Abdel-Rahman et al.^46^. All assessments were performed by an independent, blinded observer:

$$\text{Edema rate }\left(\text{\%}\right)=\frac{Vt-V0}{V0}$$

$$\text{Edema inhibition }\left(\text{\%}\right)=\frac{Ec-Et}{Ec} \times 100$$

where V0 is the paw volume (mL) before carrageenan injection and Vt is the paw volume (mL) at t hour after carrageenan injection. Ec and Et are the edema rates of control and treated groups, respectively.

Extensive monitoring practices were established in the entire study to identify any possible indications of disaffection, sickness or mood swings. Not a single subject was omitted in the experiment as well as repeated during the study period. No surgical, invasive, and terminal procedures, or tissue collection were performed; therefore, euthanasia of the rats was not required after completion of the experiment.

### *In vitro* anti-inflammatory testing

#### Cell culture

Murine macrophage RAW 264.7 cells were purchased in Nawah Scientific Inc. (Mokatam, Cairo, Egypt). The cells were grown in Dulbecco Modified Eagle Medium (DMEM) that had additional components: streptomycin (100 100 g/mL), penicillin (100 U/mL) and 10% heat-inactivated fetal bovine serum. Cultures were maintained in a humidified incubator at 37 °C in an atmosphere of 5% CO₂.

#### Cytotoxicity assay

Sulforhodamine B (SRB) assay was used in determining cell viability. In a nut shell, 100 μL of cell suspension (5 × 10^3 cells) was seeded in 96 well plates and culture was incubated to reach 24 h to adhere to the plate. Then the cells were exposed to 100 μL of media with different doses of the test compounds. Fixed, cells were added 150 μL, 10%Trichloroacetic acid (TCA) and placed in 4 °C condition one hour. Five times, Wells were cleaned with distilled water and were desiccated in air. Thereafter, 0.4 per cent SRB solution (70mL) was added into the wells and incubated in the dark at room temperature, 10 min. Three washes using 1 percent acetic acid were done to eliminate excess dye and plates allowed to dry overnight. The 150 μL of 10 mM TRIS buffer was added to bound dye and absorbance was read at 540 nm on Infinite F50 microplate reader (TECAN, Switzerland)^47,48^.

#### *In vitro* anti-inflammatory assay

In order to test the anti-inflammatory activity, RAW 264.7 cells were incubated to grow in 96 well plates by seeding them over the 24 h. Lipopolysaccharide (LPS) at the concentration of 1 μg/mL caused inflammation. Control wells had fresh medium added to them after untreated cells and the LPS group did not have any treatment added to the LPS. Test compounds were either tested alone or in combination with LPS at concentrations between 0. 001to 10 uM (LPS + Drug). Quercetin was used as the positive control. Mean production of nitric oxide (NO) was measured through the Griess reagent assay, whereby the same amounts of supernatant culture contents and reagent were combined, incubated with room temperature at darkness by ten minutes and the optical density of the mixture determined at 540 nm with the use of ELISA microplate reader^49,50^.

### Statistical analysis

The means are reported as the standard error of the mean ± (SE). One-way analysis of variance (ANOVA) was conducted as a measure to make statistical comparisons. Dunnett post hoc test was used where it showed a significant F-value (*p* ≤ 0.05). The analysis was done on data with SPSS software version 17.0 (Chicago, IL, USA).

### Determination of physicochemical properties and pharmacokinetic interactions

The SwissADME database (https://www.swissadme.ch) was utilized to predict the following physicochemical and pharmacokinetic properties for compounds **1**–**4**: lipid solubility calculated (LogP), hydrogen-bonding, interaction with Pgp, and interaction with several microsomal enzymes (CYP1A2, CYP2C9, CYP2D6, CYP3A4) ^51^.

### Virtual screening of inflammation-related genes

The GeneCards database (https://www.genecards.org) was utilized to identify genes associated with inflammation, using the keyword “inflammation” ^52^. Microsoft Excel was used to collect and process data for later steps.

### In silico screening of gene targets of compounds 1–4

The possible targets of compounds **1–4** on genes were discovered with the assistance of the SwissTargetPrediction platform^40^. The tool predicts probable targets by contrasting the molecular structure of each compound with that of the known ligand of individual proteins based on both two-dimensional and three-dimensional structural similarities, and on the number of ligands having analogous properties. A probability score is created that will show the probability of interaction. This study identified that any predicted targets whose probability value was larger than zero were used further in analyses.

### Identification of common genes between inflammation and compounds 1–4

Venn diagrams (web-server available online: https://bioinfogp.cnb.csic.es/tools/venny/) were utilized to identify common targets between the isolated test compounds, and inflammation genes obtained from the previous steps. Overlapping genes for individual compounds were identified^41^. The Cytoscape bioinformatics software (version 3.10.1) was used to highlight common, as well as exclusive gene targets of each compound^42^.

### Protein–protein interaction (PPI) analysis

The target genes of the screened compounds **1–4** were scrutinized as well as the possible and known protein–protein interactions based on the STRING functional protein association networks database (https://m.string-db.org) ^53^. This process allowed identification of a small number of inflammation associated genes having most extensive interaction profiles. It only included interactions with a confidence score having a value greater than 0.4. A visualization of the resulting PPI network together with its analysis in terms of Cytoscape software was performed where all the genes were ranked based on their degree centrality (DC) value which is the cumulative number of PPIs associated to a given gene. Short descriptions about the highest DC value genes were created.

### Enrichment analysis of targeted genes

The ShinyGO database (https://bioinformatics.sdstate.edu/go/) analyzed the inflammation-related gene targets of compounds **1**–**4**^54^. This analysis is based on gene ontology and is performed to determine the biological processes influenced by these genes. This analysis determines the number of genes related to a biological aspect, and assigns an FDR significance score. Lower FDR values indicate a stronger prediction, or higher significance.

### Molecular docking procedure

Molecular docking simulations of the binding interactions of compounds 14 in the MAOA and MAOB isoforms of monoamine oxidase have been carried out. They were picked on the basis that both MAOA and MAOB were used as widely predicted targets of compounds **1**, **2**, and **4**, and the enzymes otherwise coded by them significantly contribute to the inflammatory pathways. The compounds 2D chemical structures were initially drawn in ChemDraw (version 7.0) then exported in Mol 3D format. These were then transformed to PDB files using BIOVIA discovery studio 2022 visualizer. Crystal structures of MAOA (PDB ID: 2BXR) and MAOB (PDB ID: 7P4F) have been retrieved at the Protein Data Bank^55^. The preparation of protein structures involved isolation of monomeric form and deletion of co-crystallized ligands as well as water molecules in BIOVIA Discovery Studio Visualizer 2022^56^. The ligand and protein files were then converted to Autodock PDBQT using the Autodock Tools (1.5.6) that was also used in defining the active site by modifying the grid box such that important amino acid residues that interact with the native ligands were included in the grid box. Autodock Vina (version 1.12) was used to do docking and the lowest binding energy compounds-protein complexes were further analyzed. 3D interaction visualization images were then created with Discovery Studio visualizer and UCSF ChimeraX (version 1.14)^57^.

### Molecular dynamic simulation

Molecular dynamics (MD) simulations in GROMACS (version 2018.6) with the OPLS-AA/L force field were carried out in the current study. Two monoamine oxidase (MAO-A and MAO-B) isoforms were explored, where the three-dimensional structures were improved through the usage of the DockPrep tool implemented in UCSF ChimeraX (version 1.14)^58^. Compound **4** was parameterized using the SwissParam webserver (https://www.swissparam.ch) ^59^. The MD simulations were performed for 20 ns, as following the same method in a previous study^60^. Essential MDS parameters including RMSD, RMSF, and Hydrogen bonding analysis were evaluated.

## Supplementary Information

Below is the link to the electronic supplementary material.

Supplementary Material 1

## Data Availability

All data generated or analysed during this study are included in this published article and its supplementary information files.
