# Supplementary material for: Decoding the anti-inflammatory properties of sesquiterpene coumarins from the active fractions of Ferula assa-foetida through integrated experimental and in silico analyses
Source: Sci Rep. 2025 Nov 26;15:42145. doi: 10.1038/s41598-025-26010-3 (PMC12658108; doi:10.1038/s41598-025-26010-3)
Supplement: Supplementary file 1 — Supplementary Material 1 [file 41598_2025_26010_MOESM1_ESM.docx]

Supplementary data

Anti-inflammatory Sesquiterpene Coumarins from the Active Fractions of *Ferula assa-foetida*: In Silico Analysis Endorse Experimental data

**Mubarak A. Alamri^1^, Gamal A. Soliman^2*^, Mohammed A. Alamri^2^, Rehab F. Abdel-Rahman^3^, Ahmed W. Alhalabi^4^, Raid S. Almahmoud^4^, Ibrahim M. Alsagheir^4^, Abdulrahman G. Alharbi ^5^, Maged S. Abdel-Kader^6^**

^1^Department of Pharmaceutical Chemistry, College of Pharmacy, Prince Sattam Bin Abdulaziz University, Al-Kharj 11942, Saudi Arabia.

^2^Department of Pharmacology and Toxicology, College of Pharmacy, Prince Sattam Bin Abdulaziz University, Al-Kharj 11942, Saudi Arabia.

^3^Department of Pharmacology, National Research Centre, Giza 12622, Egypt.

^4^College of Pharmacy, Prince Sattam Bin Abdulaziz University, Al-Kharj 11942, Saudi Arabia.

^5^ Maternity and Children’s Hospital, Ministry of Health, Al-Kharj 11942, Saudi Arabia.

^6^ Department of Pharmacognosy, College of Pharmacy, Prince Sattam Bin Abdulaziz University, Al-Kharj 11942, Saudi Arabia.

*Author to whom correspondence should be addressed: [g.soliman@psau.edu.sa](mailto:g.soliman@psau.edu.sa)

**Table** **of** **contents**

**Title** **Page**

| **Figure** **S1**: ^1^HNMR spectrum of **1** in C6D6. | 4 |
| --- | --- |
| **Figure** **S2**: ^1^HNMR spectrum of **1** in C6D6 (Exp.). | 4 |
| **Figure** **S3**: ^1^HNMR spectrum of **1** in C6D6 (Exp.). | 5 |
| **Figure** **S4**: ^1^HNMR spectrum of **1** in C6D6 (Exp.). | 5 |
| **Figure** **S5**: ^13^CNMR spectrum of **1** in C6D6. | 6 |
| **Figure** **S6**: ^13^CNMR spectrum of **1** in C6D6. | 6 |
| **Figure** **S7**: ^13^CNMR spectrum of **1** in C6D6 (Exp.). | 7 |
| **Figure** **S8**: DEPT135 spectrum of **1** in C6D6**.** | 7 |
| **Figure** **S9**: COSY spectrum of **1**in C6D6. | 8 |
| **Figure** **S10**: HSQC spectrum of **1** in C6D6. | 8 |
| **Figure** **S11**: HRESIMS spectrum of **1** (Negative mode)**.** | 9 |
| **Figure** **S12**: HRESIMS spectrum of **1** (Positive mode)**.** | 9 |
| **Figure** **S13**: ^1^HNMR spectrum of **2** in C6D6. | 10 |
| **Figure** **S14**: ^1^HNMR spectrum of **2** in C6D6 (Exp.). | 10 |
| **Figure** **S15**: ^1^HNMR spectrum of **2** in C6D6 (Exp.). | 11 |
| **Figure** **S16**: ^13^CNMR spectrum of **2** in C6D6. | 11 |
| **Figure** **S17**: ^13^CNMR spectrum of **2** in C6D6 (Exp.). | 12 |
| **Figure** **S18**: ^13^CNMR spectrum of **2** in C6D6 (Exp.). | 12 |
| **Figure** **S19**: ^13^CNMR spectrum of **2** in C6D6 (Exp.). | 13 |
| **Figure** **S20**: DEPT135 spectrum of **2** in C6D6. | 13 |
| **Figure** **S21**: COSY spectrum of **2** in C6D6. | 14 |
| **Figure** **S22**: HSQC spectrum of **2** in C6D6. | 14 |
| **Figure** **S23**: HRESIMS spectrum of **2.** | 15 |
| **Figure** **S24**: ^1^HNMR spectrum of **3** in C6D6. | 16 |
| **Figure** **S25**: ^1^HNMR spectrum of **3** in C6D6 (Exp.). | 16 |
| **Figure** **S26**: ^1^HNMR spectrum of **3** in C6D6 (Exp.). | 17 |
| **Figure** **S27**: ^13^CNMR spectrum of **3** in C6D6. | 17 |
| **Figure** **S28**: ^13^CNMR spectrum of **3** in C6D6 (Exp.). | 18 |
| **Figure** **S29**: ^13^CNMR spectrum of **3** in C6D6 (Exp.). | 18 |
| **Figure** **S30**: DEPT135 spectrum of **3** in C6D6. | 19 |
| **Figure** **S31**: COSY spectrum of **3** in C6D6. | 19 |
| **Figure** **S32**: COSY spectrum of **3** in C6D6 (Exp.). | 20 |
| **Figure** **S33**: HSQC spectrum of **3** in C6D6. | 20 |
| **Figure** **S34**: ^1^HNMR spectrum of **3** in CD3OD. | 21 |
| **Figure** **S35**: ^1^HNMR spectrum of **3** in CD3OD (Exp.). | 21 |
| **Figure** **S36**: ^1^HNMR spectrum of **3** in CD3OD (Exp.). | 22 |
| **Figure** **S37**: ^13^CNMR spectrum of **3** in CD3OD. | 22 |
| **Figure** **S38**: ^13^CNMR spectrum of **3** in CD3OD (Exp.). | 23 |
| **Figure** **S39**: DEPT135 spectrum of **3** in CD3OD. | 23 |
| **Figure** **S40**: COSY spectrum of **3** in CD3OD. | 24 |
| **Figure** **S41**: COSY spectrum of **3** in CD3OD (Exp.). | 24 |
| **Figure** **S42**: COSY spectrum of **3** in CD3OD (Exp.). | 25 |
| **Figure** **S43**: HSQC spectrum of **3** in CD3OD. | 25 |
| **Figure** **S44**: HSQC spectrum of **3** in CD3OD (Exp.). | 26 |

| **Figure** **S45**: HRESIMS spectrum of **3** (Negative mode). | 27 |
| --- | --- |
| **Figure** **S46**: HRESIMS spectrum of **3** (Positive mode). | 27 |
| **Figure** **S47**: ^1^HNMR spectrum of **4** in CD3OD. | 28 |
| **Figure** **S48**: ^1^HNMR spectrum of **4** in CD3OD (Exp.). | 28 |
| **Figure** **S49**: ^13^CNMR spectrum of **4** in CD3OD. | 29 |
| **Figure** **S50**: ^13^CNMR spectrum of **4** in CD3OD (Exp.). | 29 |
| **Figure** **S51**: DEPT135 spectrum of **4** in CD3OD. | 30 |
| **Figure** **S52**: COSY spectrum of **4** in CD3OD. | 30 |
| **Figure** **S53**: HSQC spectrum of **4** in CD3OD. | 31 |
| **Figure** **S54**: HSQC spectrum of **4** in CD3OD (Exp.). | 31 |
| **Figure** **S55**: ^1^HNMR spectrum of **4** in Pyridine d5. | 32 |
| **Figure** **S56**: ^1^HNMR spectrum of **4** in Pyridine d5 (Exp.). | 32 |
| **Figure** **S57**: ^13^CNMR spectrum of **4** in Pyridine d5. | 33 |
| **Figure** **S58**: ^13^CNMR spectrum of **4** in Pyridine d5 (Exp.). | 33 |
| **Figure** **S59**: DEPT135 spectrum of **4** in Pyridine d5. | 34 |
| **Figure** **S60**: DEPT135 spectrum of **4** in Pyridine d5 (Exp.). | 34 |
| **Figure** **S61**: HRESIMS spectrum of **4**. | 35 |


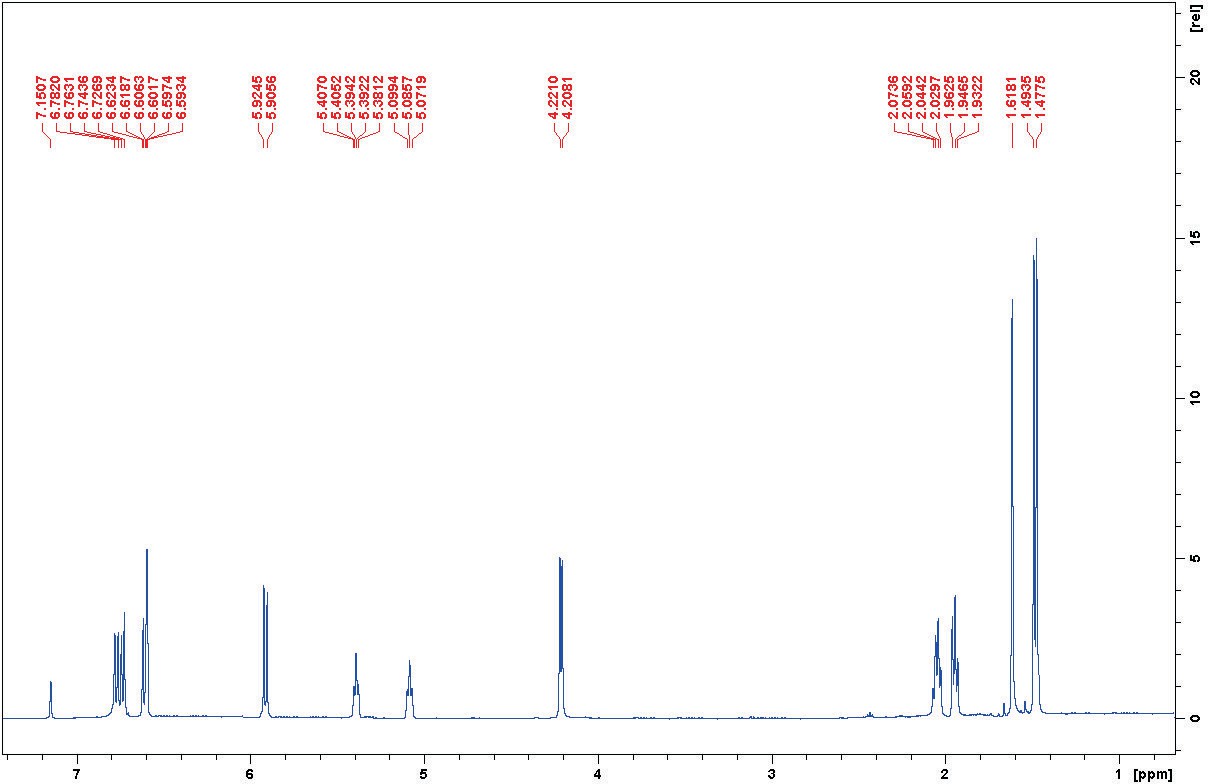


**Figure** **S1**: ^1^HNMR spectrum of **1** in C6D6.


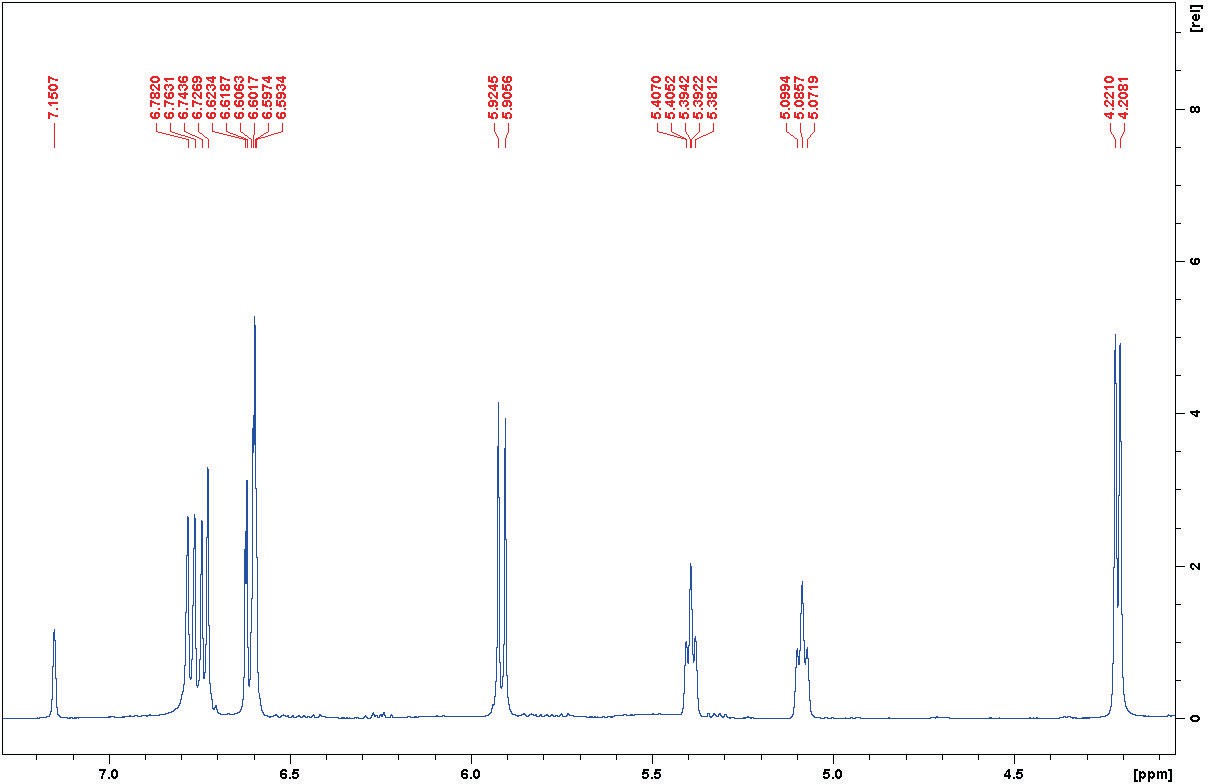


**Figure** **S2**: ^1^HNMR spectrum of **1** in C6D6 (Exp.).


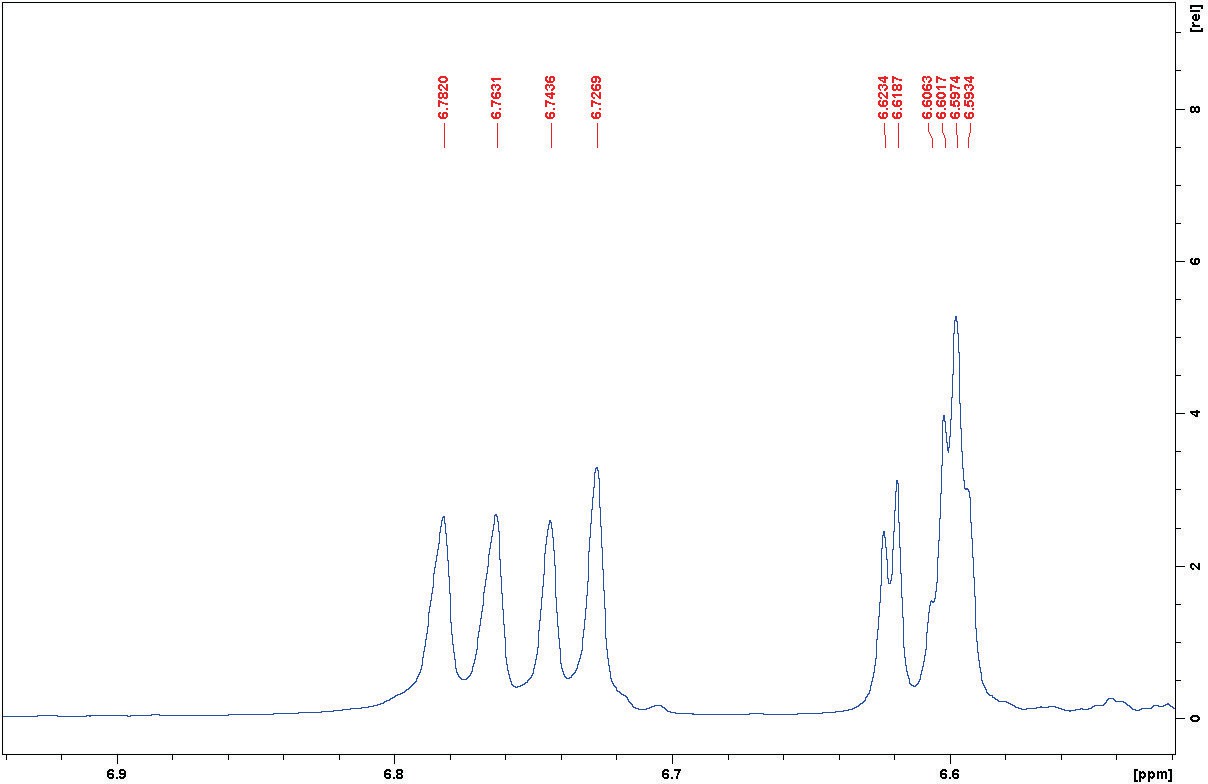


**Figure** **S3**: ^1^HNMR spectrum of **1** in C6D6 (Exp.).


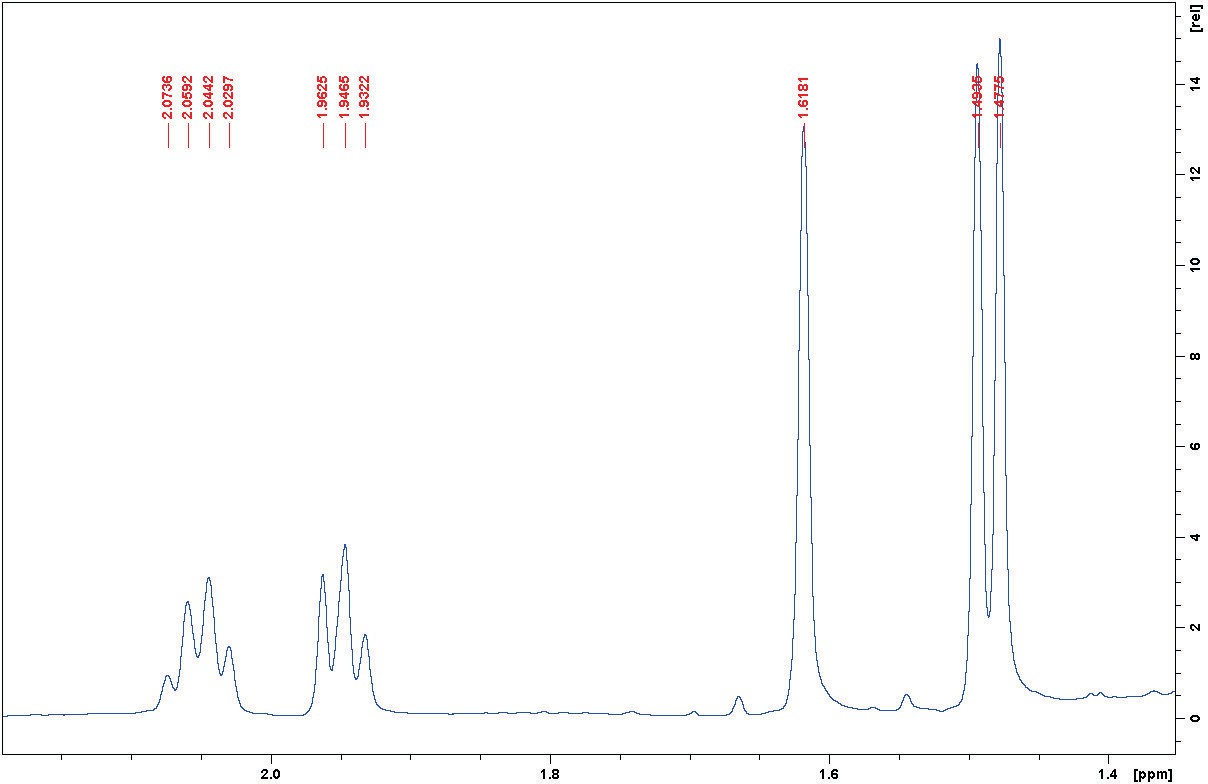


**Figure** **S4**: ^1^HNMR spectrum of **1** in C6D6 (Exp.).


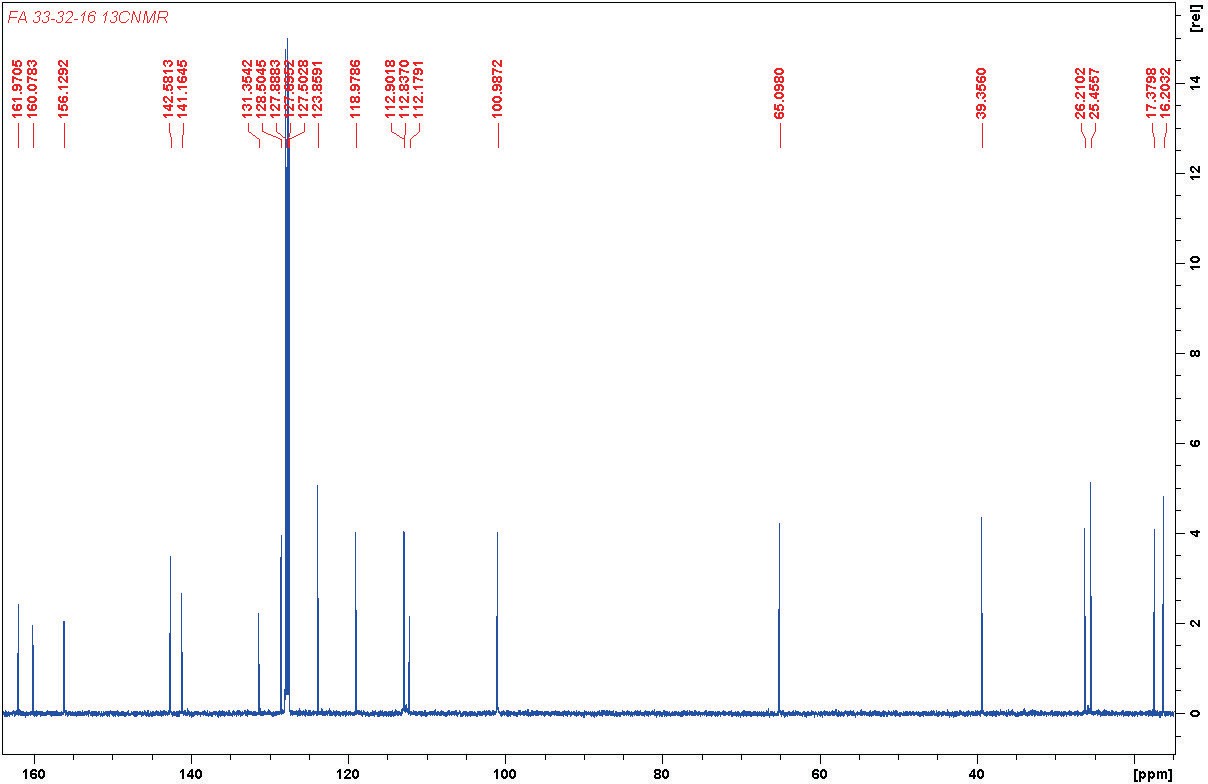


**Figure** **S5**: ^13^CNMR spectrum of **1** in C6D6.


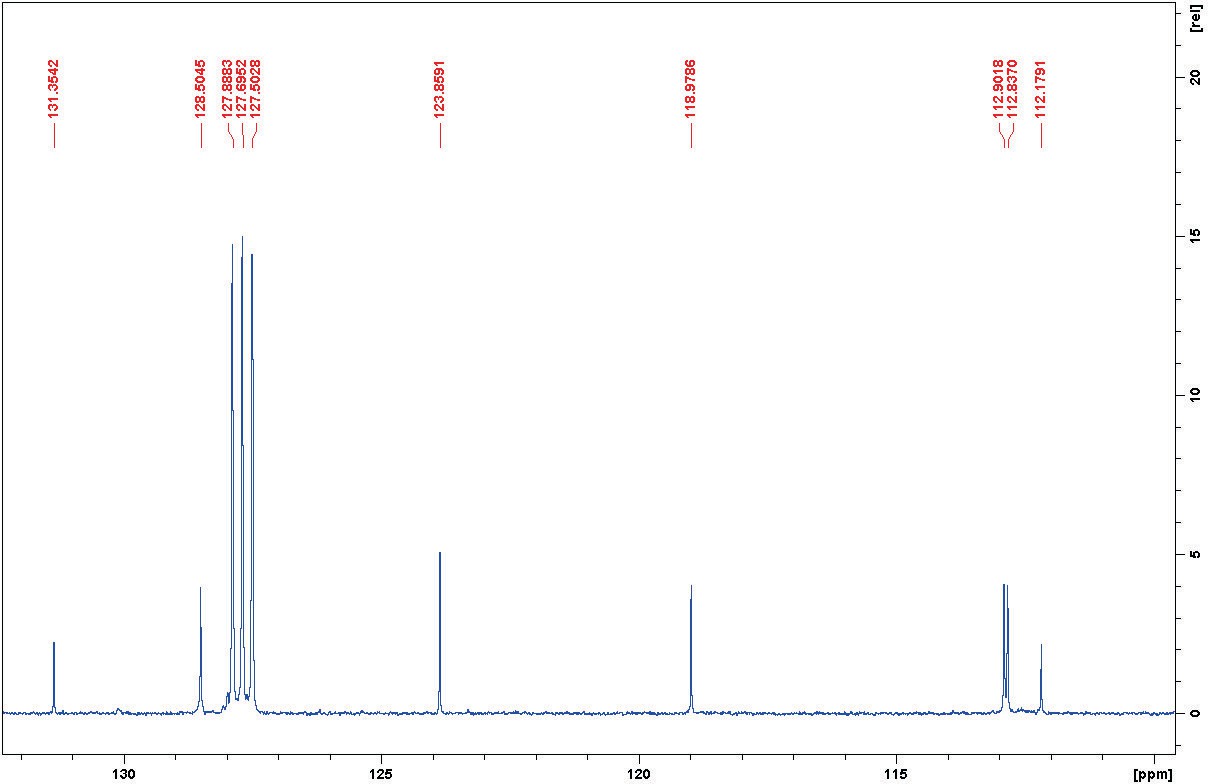


**Figure** **S6**: ^13^CNMR spectrum of **1** in C6D6.


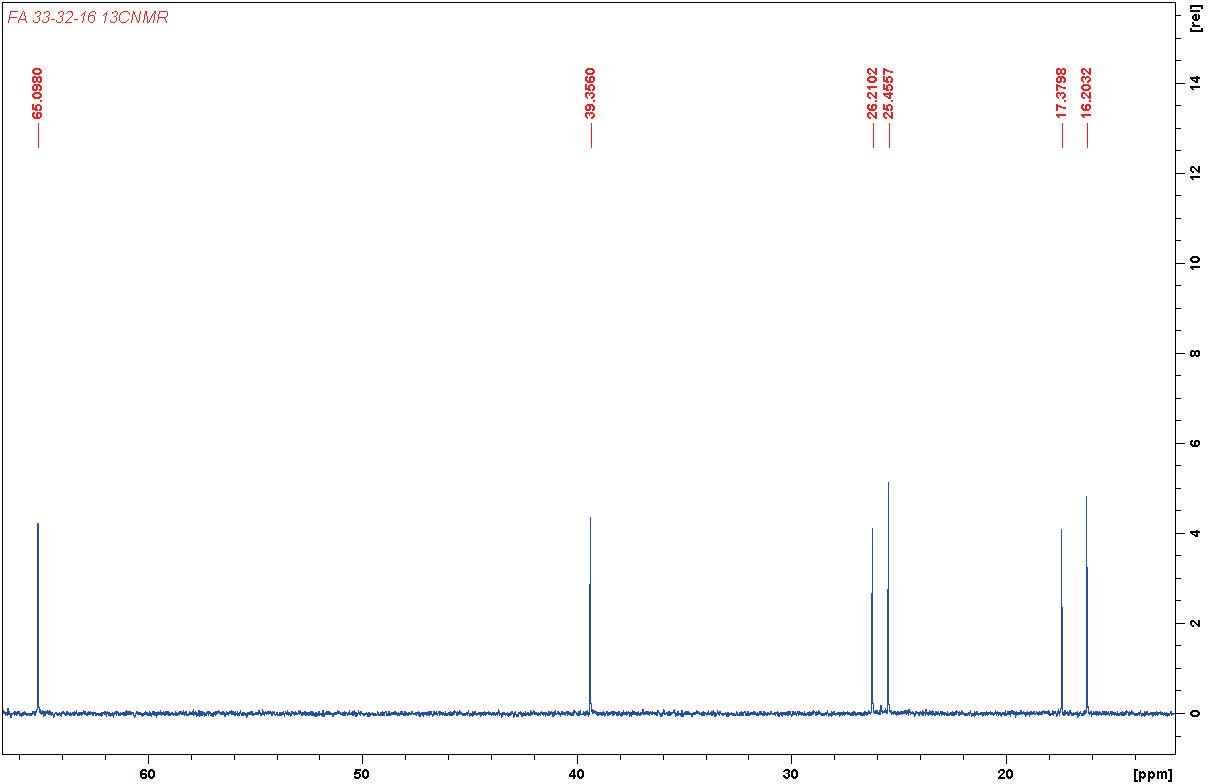


**Figure** **S7**: ^13^CNMR spectrum of **1** in C6D6 (Exp.).


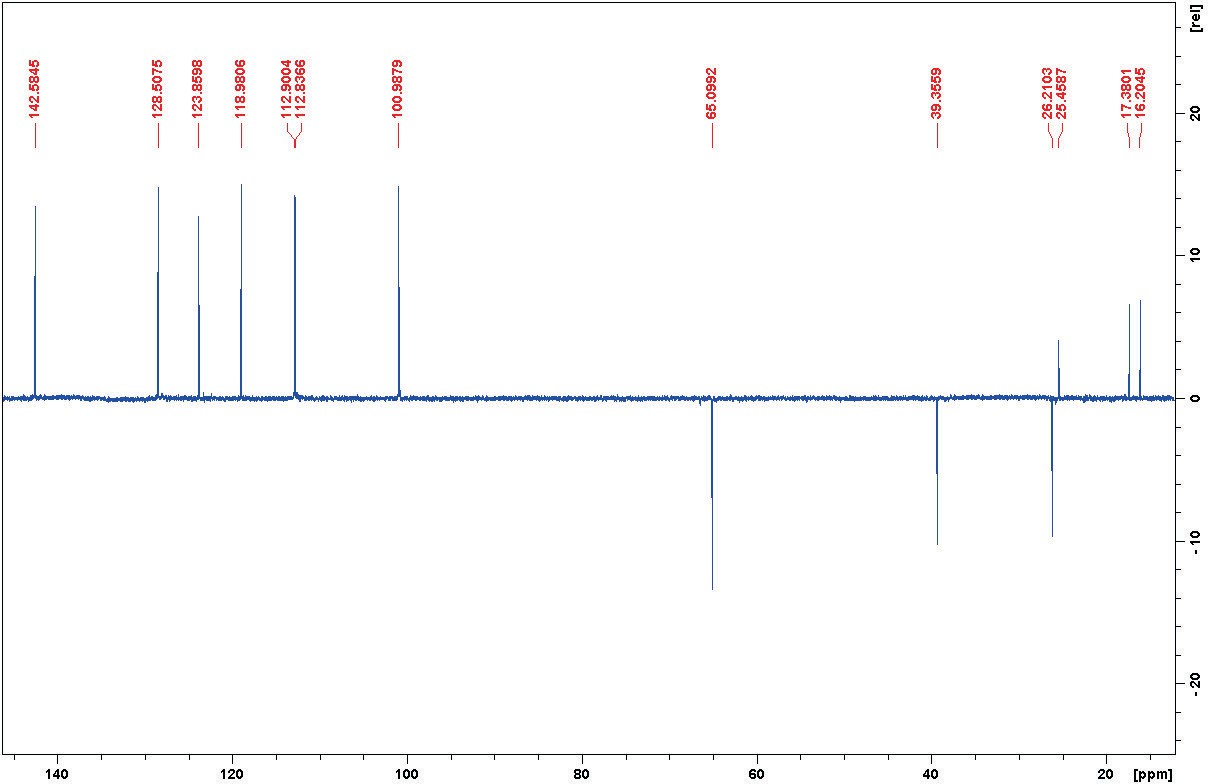


**Figure** **S8**: DEPT135 spectrum of **1** in C6D6**.**


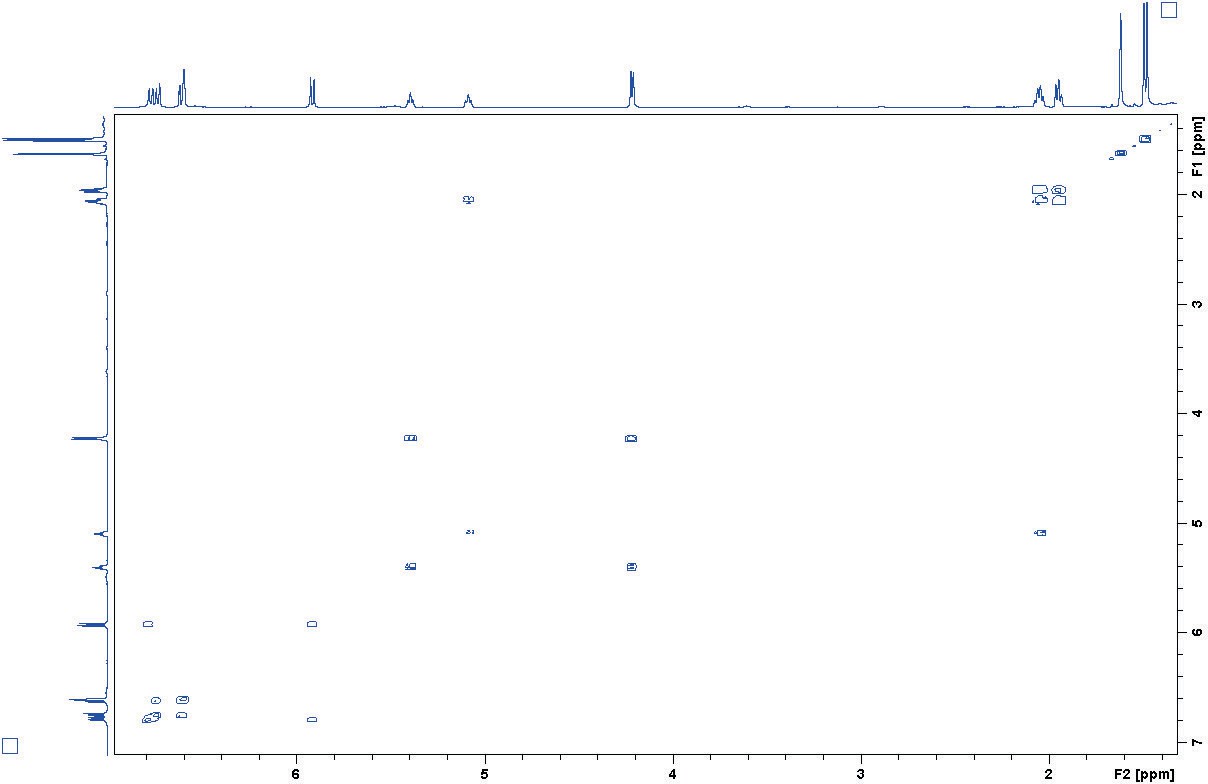


**Figure** **S9**: COSY spectrum of **1**in C6D6.


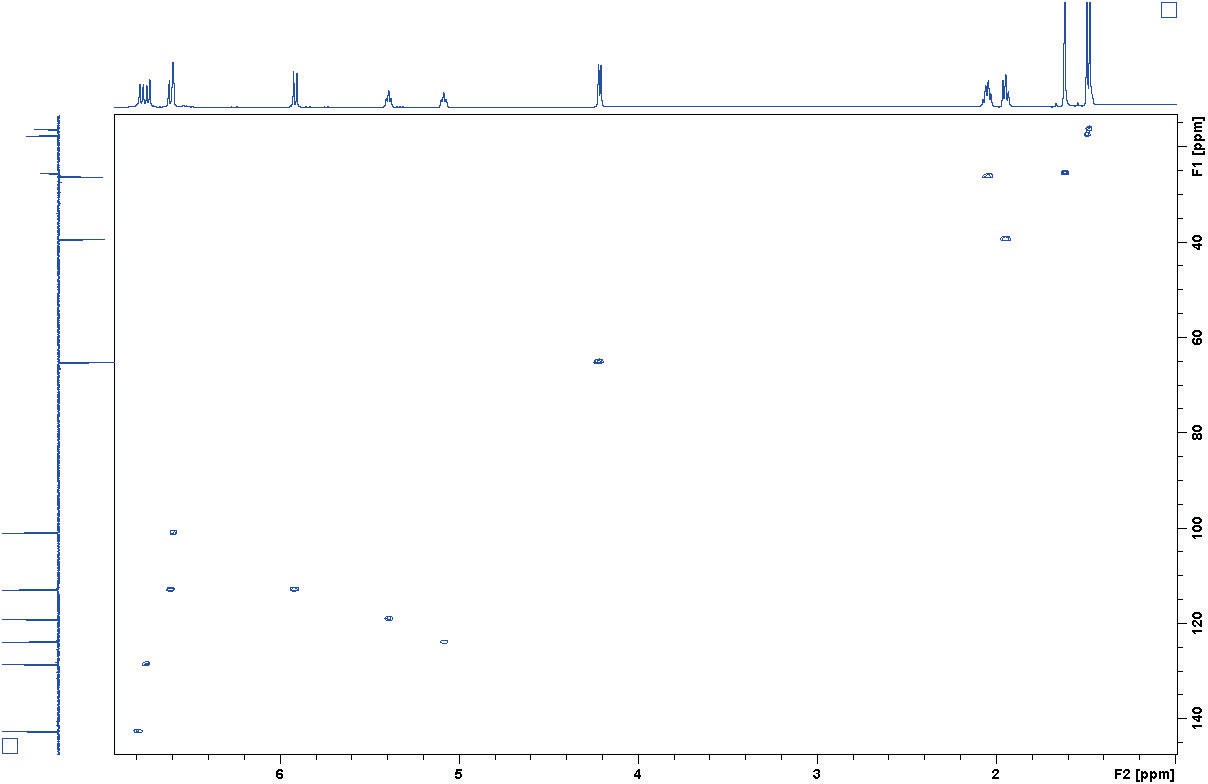


**Figure** **S10**: HSQC spectrum of **1** in C6D6.


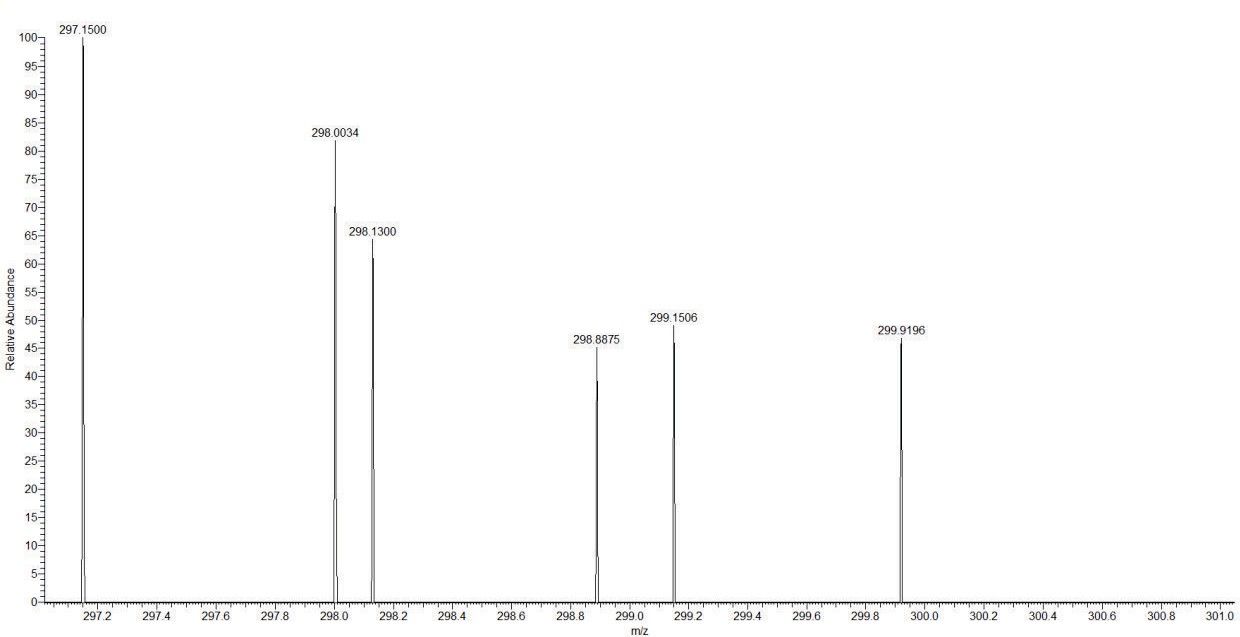


**Figure** **S11**: HRESIMS spectrum of **1** (Negative mode)**.**


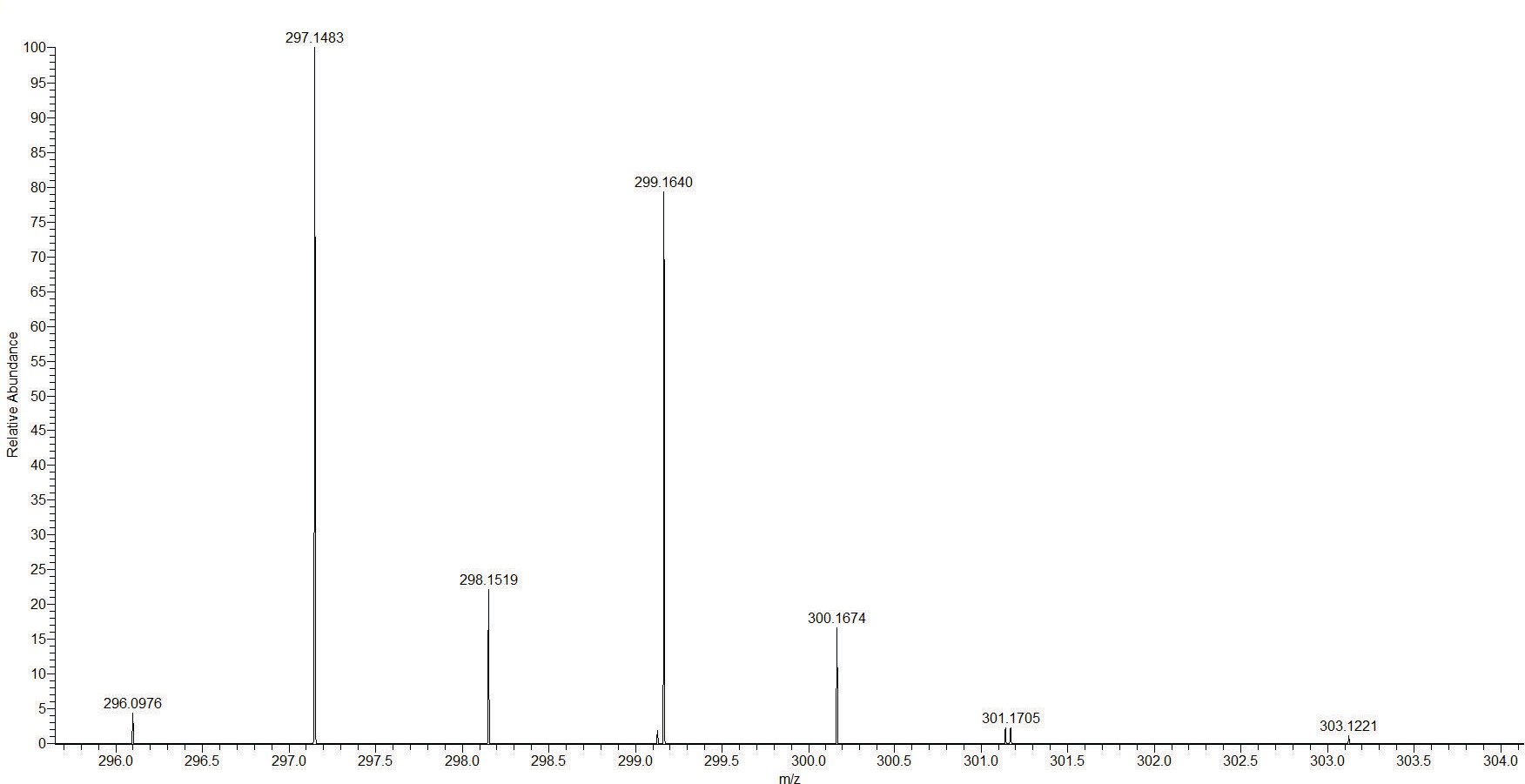


**Figure** **S12**: HRESIMS spectrum of **1** (Positive mode)**.**


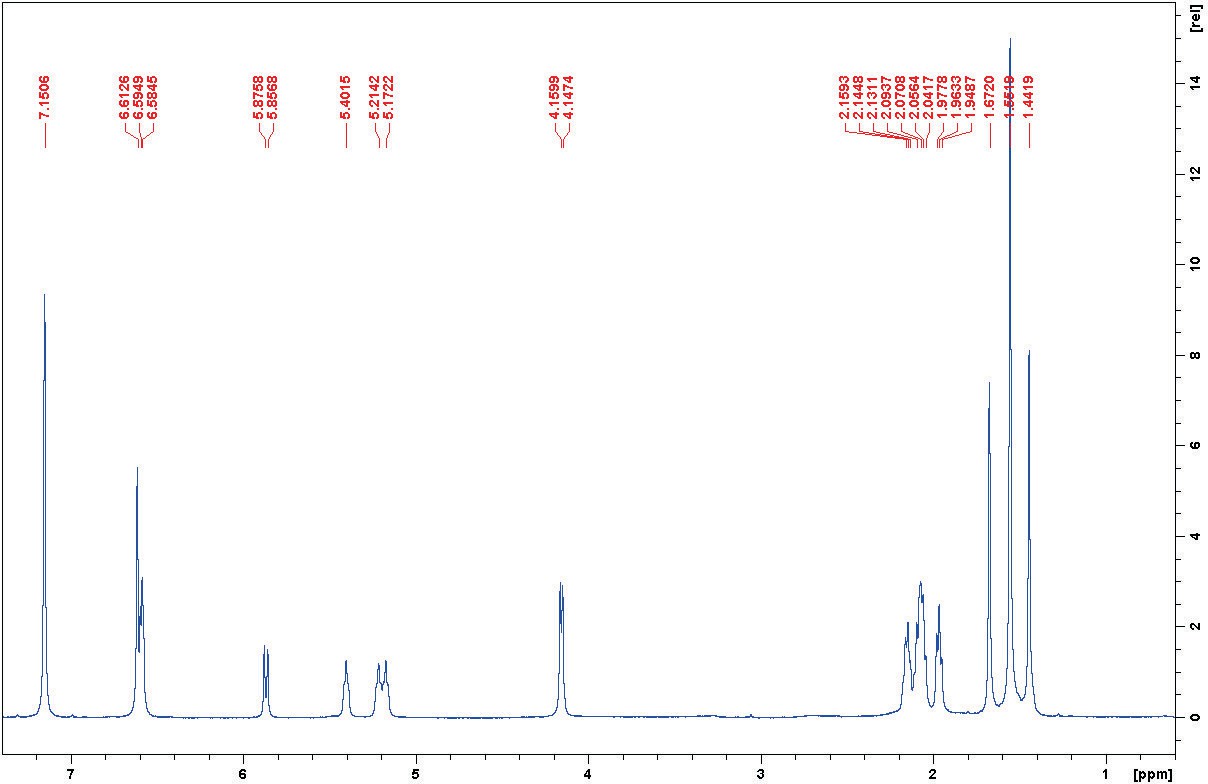


**Figure** **S13**: ^1^HNMR spectrum of **2** in C6D6.


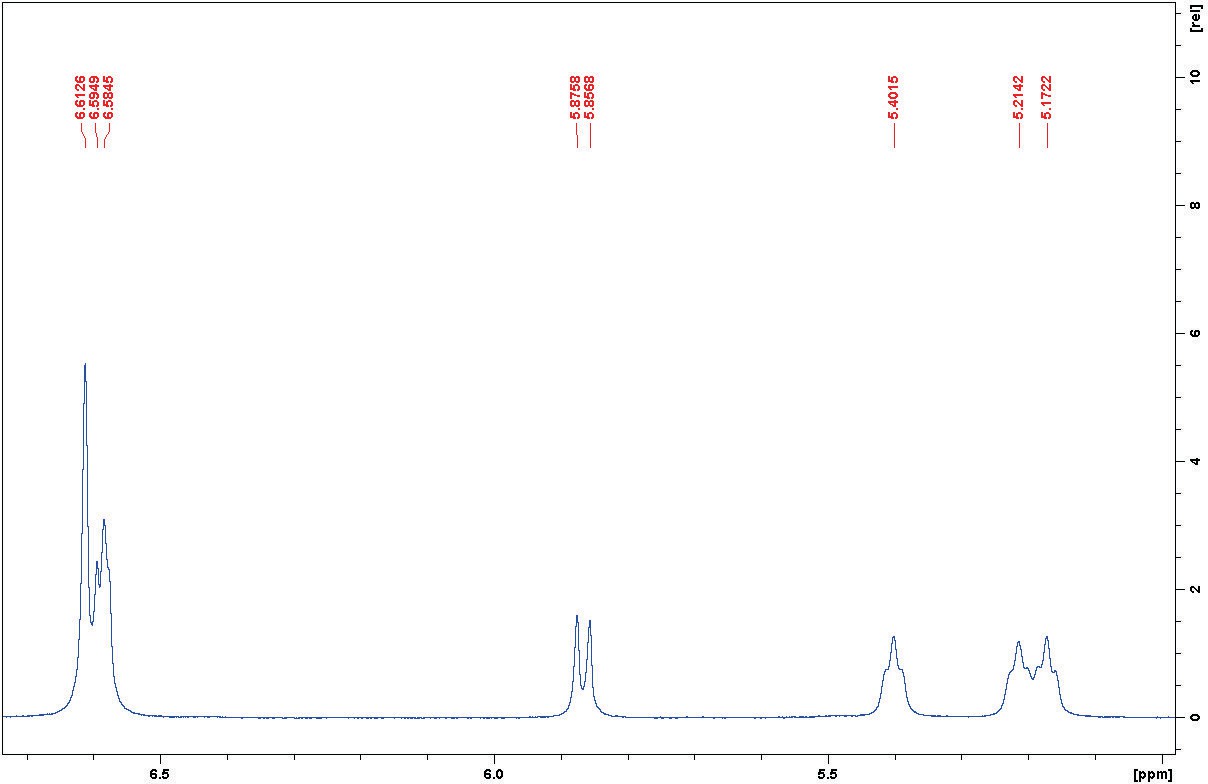


**Figure** **S14**: ^1^HNMR spectrum of **2** in C6D6 (Exp.).


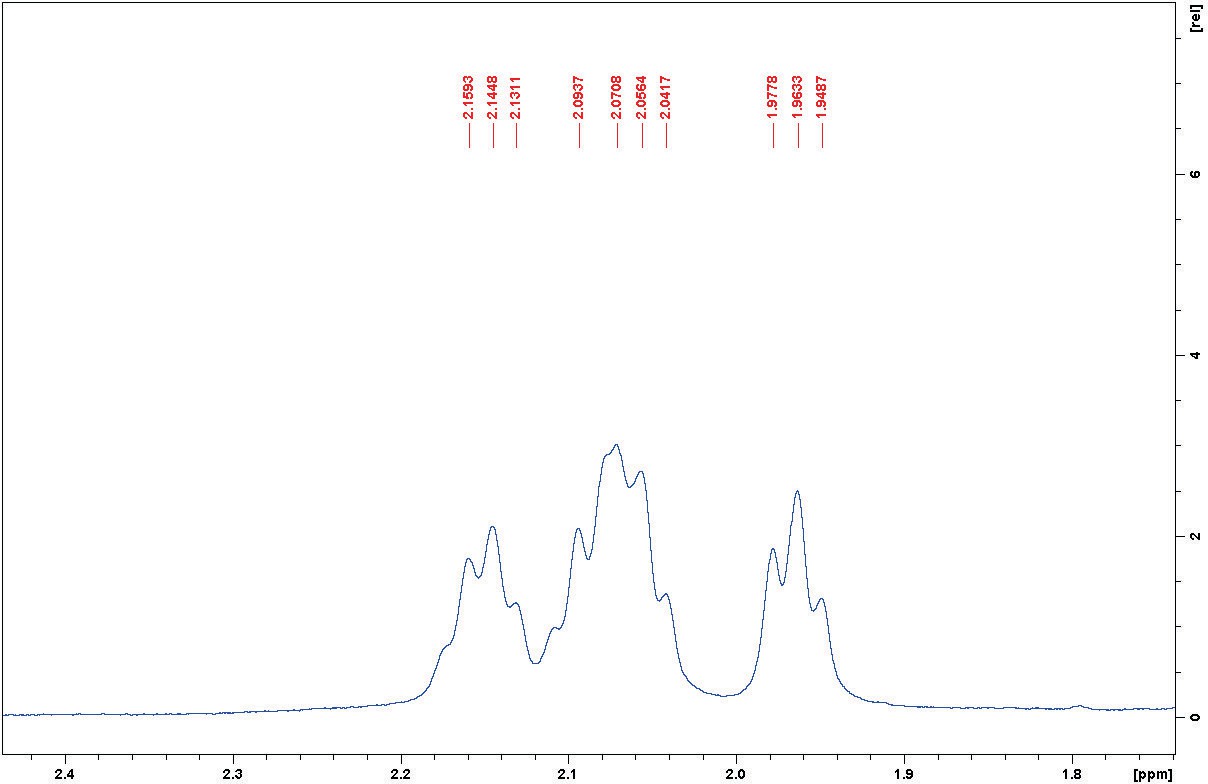


**Figure** **S15**: ^1^HNMR spectrum of **2** in C6D6 (Exp.).


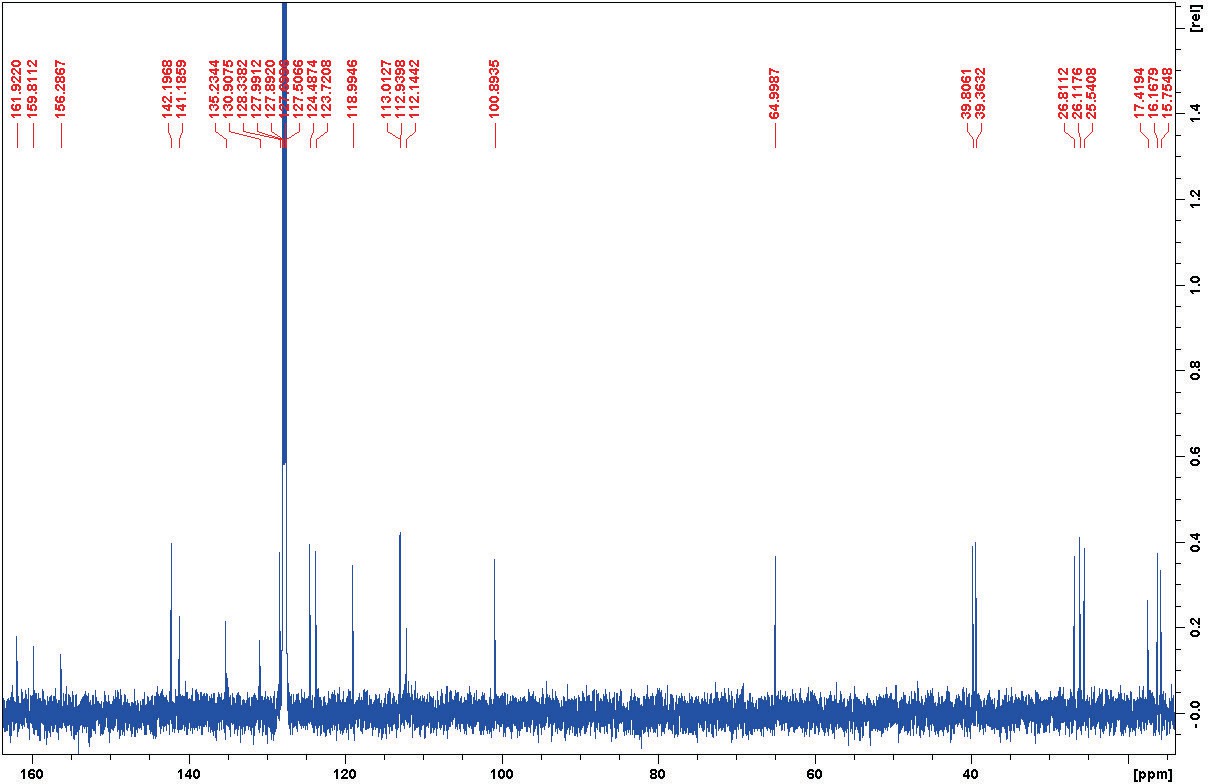


**Figure** **S16**: ^13^CNMR spectrum of **2** in C6D6.


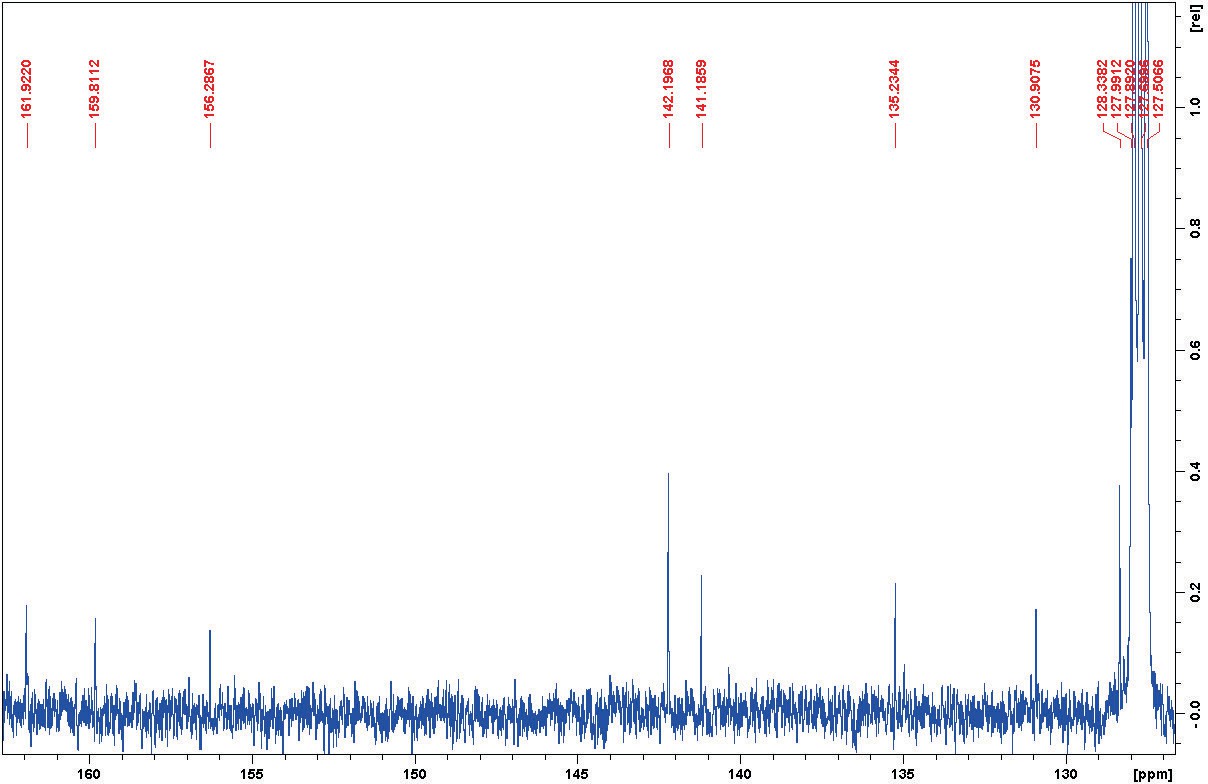


**Figure** **S17**: ^13^CNMR spectrum of **2** in C6D6 (Exp.).


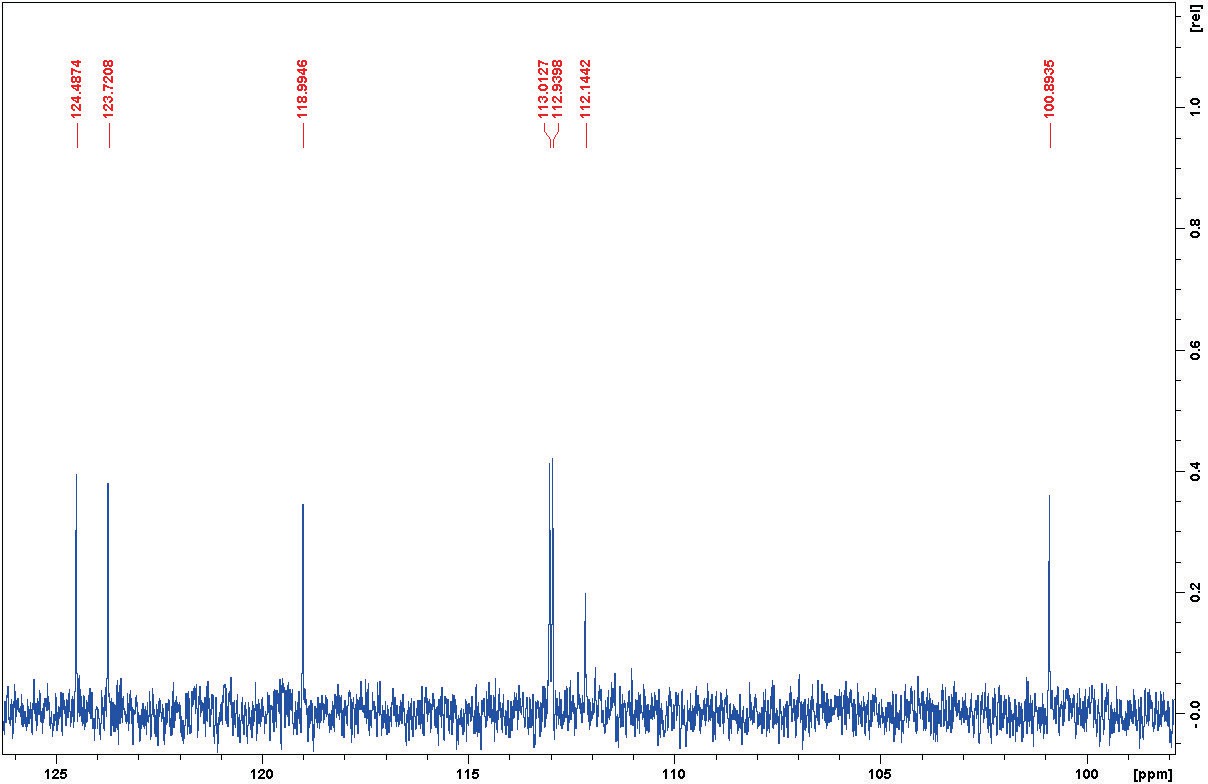


**Figure** **S18**: ^13^CNMR spectrum of **2** in C6D6 (Exp.).


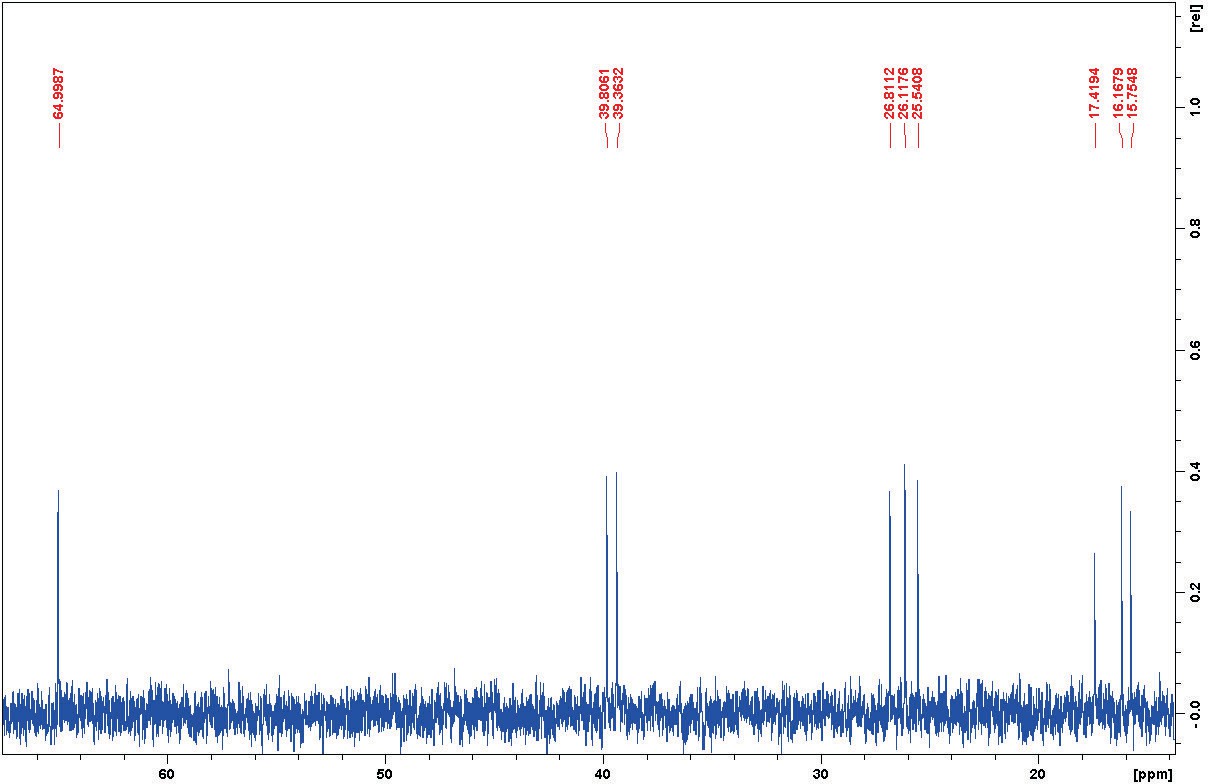


**Figure** **S19**: ^13^CNMR spectrum of **2** in C6D6 (Exp.).


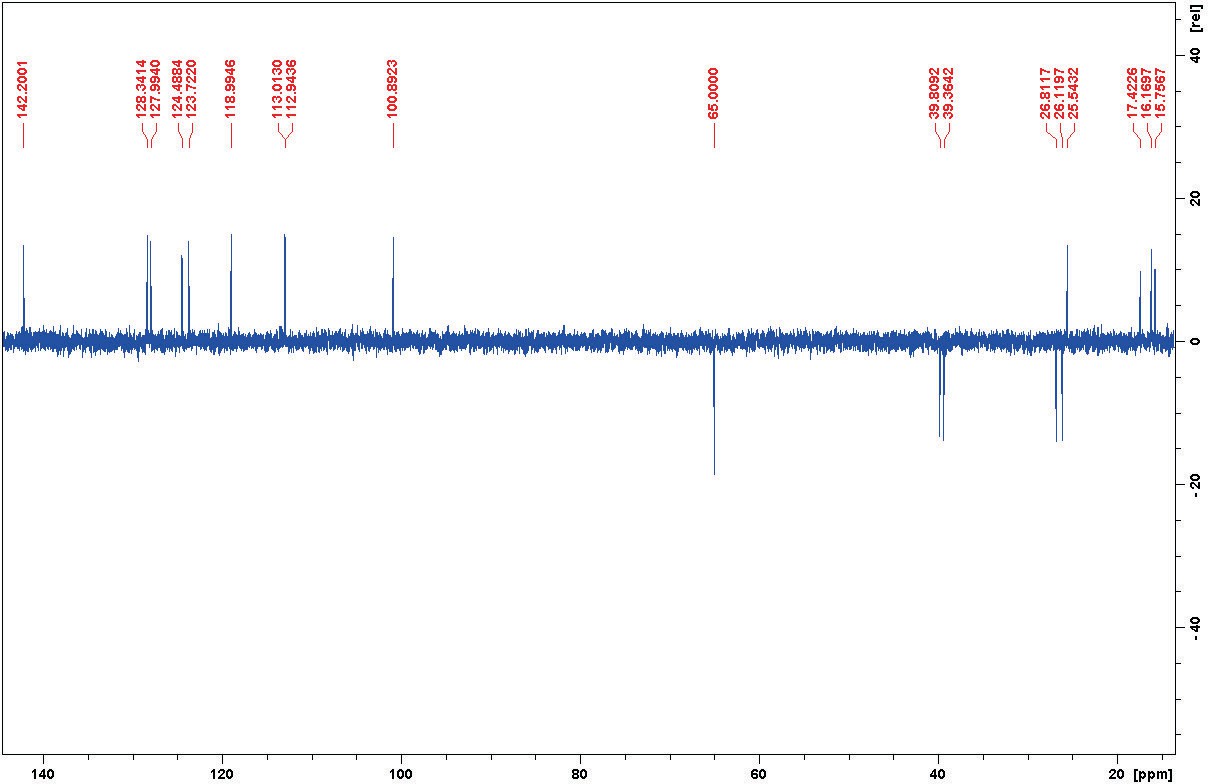


**Figure** **S20**: DEPT135 spectrum of **2** in C6D6.


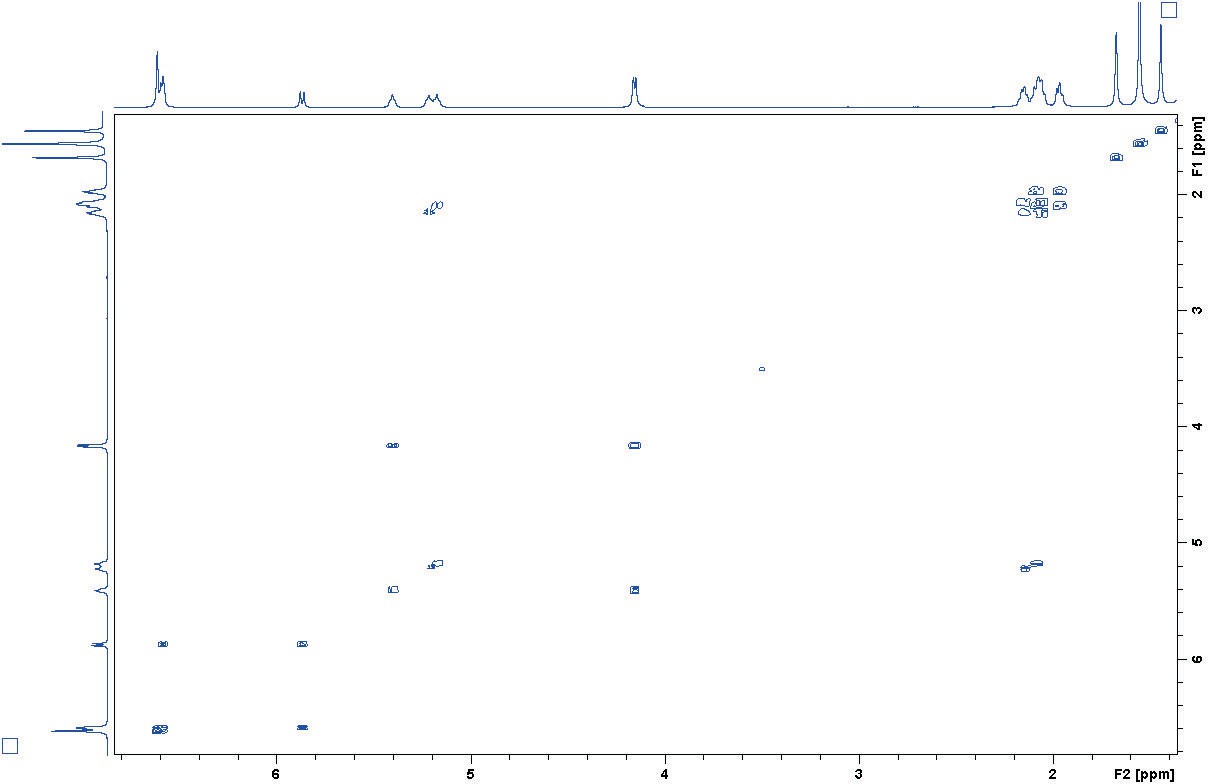


**Figure** **S21**: COSY spectrum of **2** in C6D6.


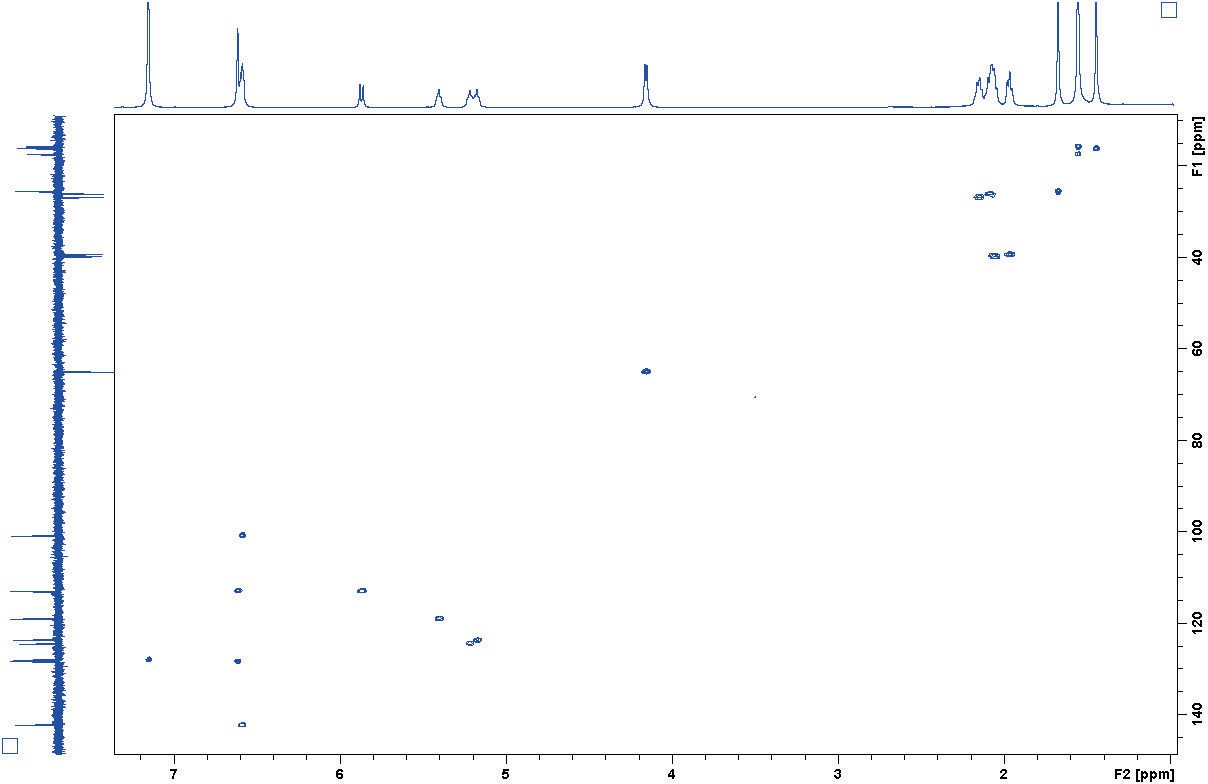


**Figure** **S22**: HSQC spectrum of **2** in C6D6.


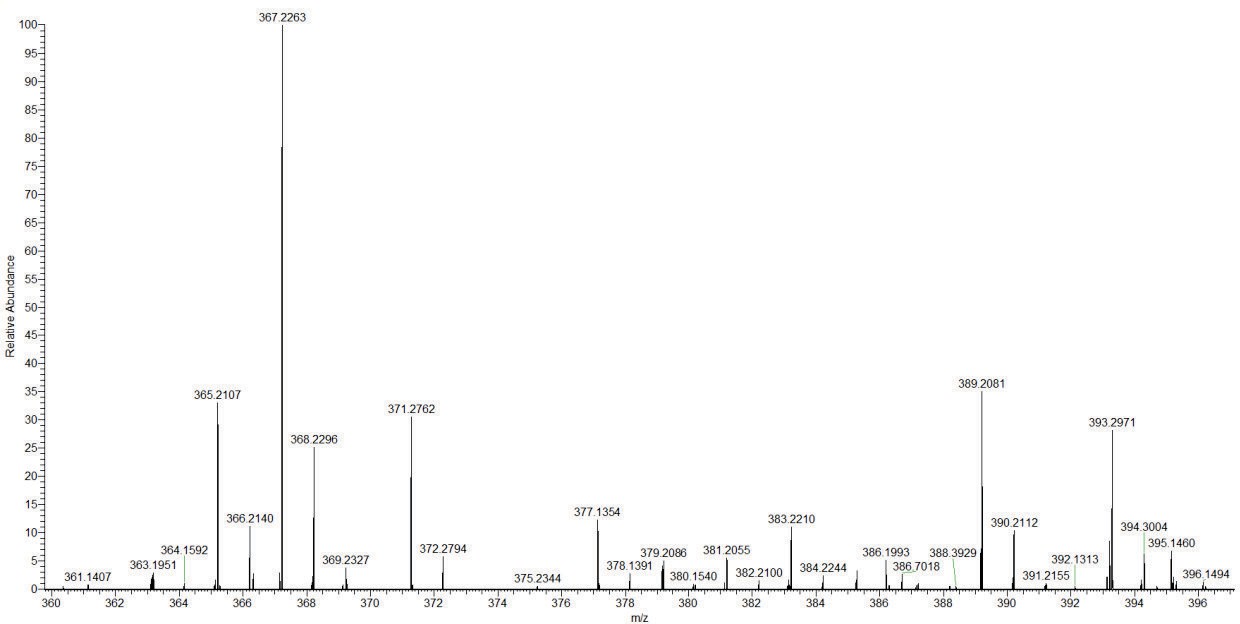


**Figure** **S23**: HRESIMS spectrum of **2.**


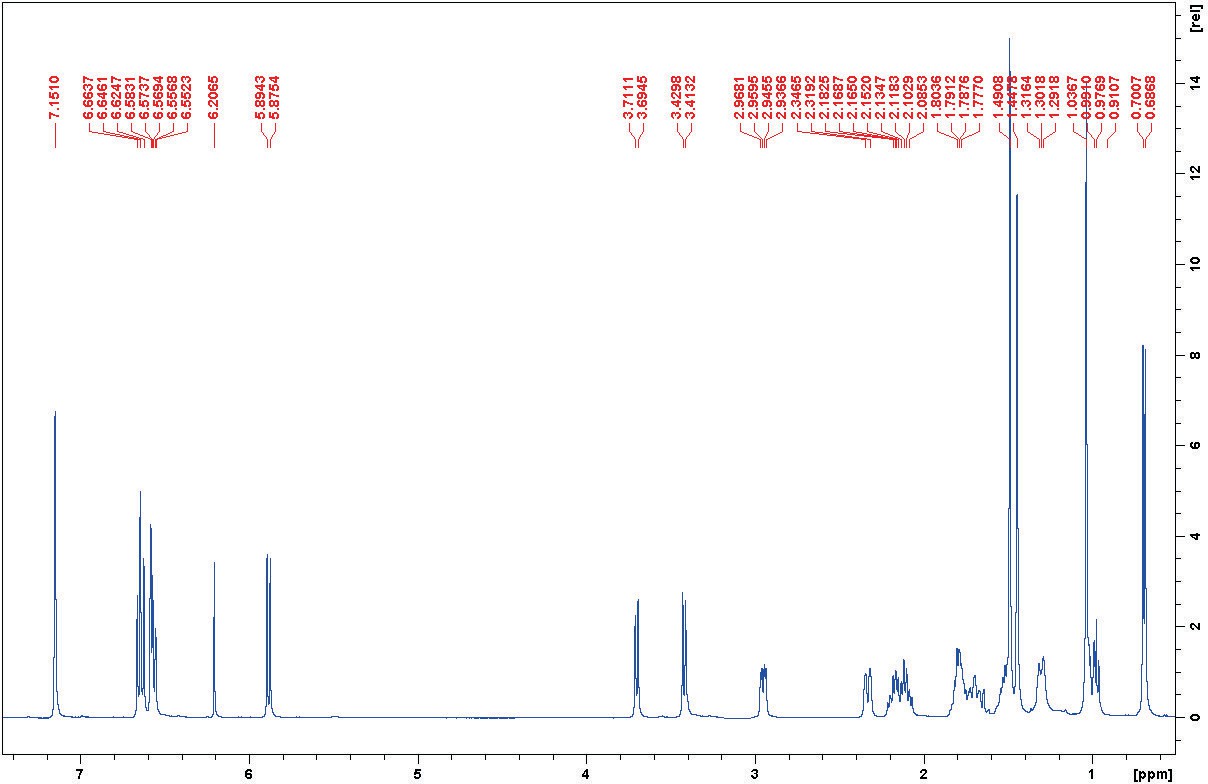


**Figure** **S24**: ^1^HNMR spectrum of **3** in C6D6.


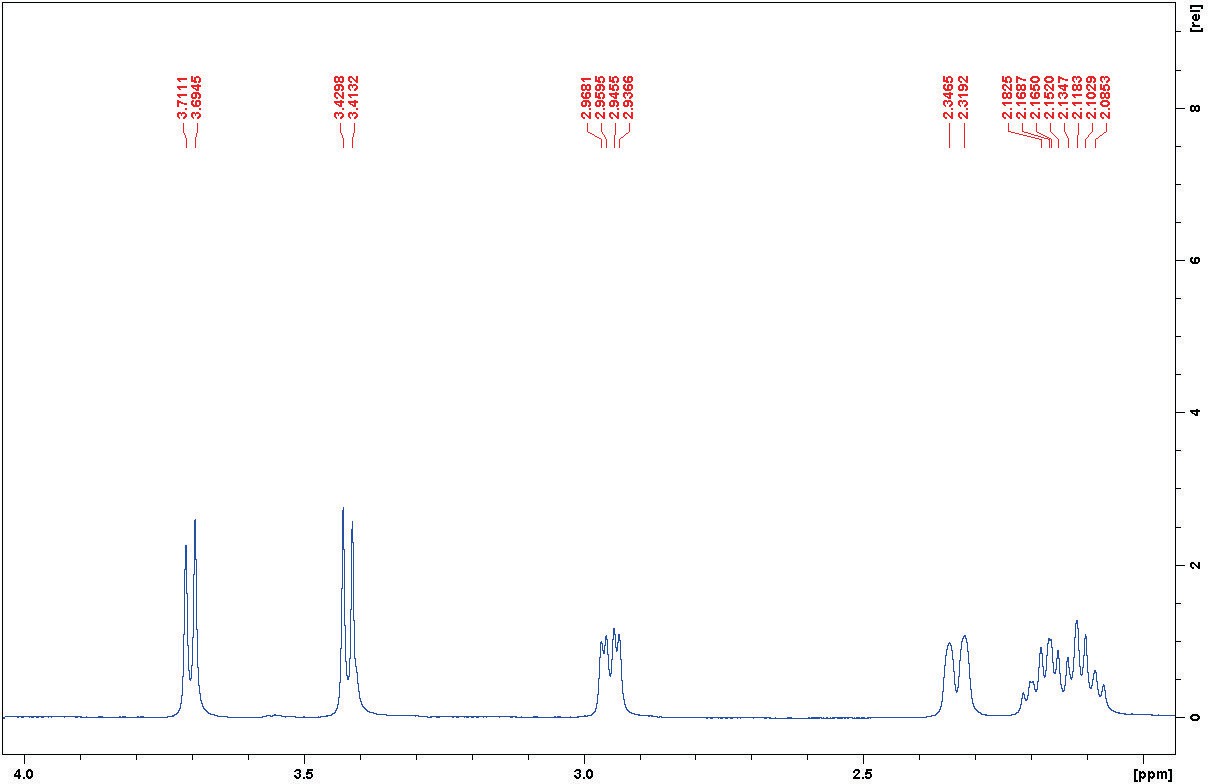


**Figure** **S25**: ^1^HNMR spectrum of **3** in C6D6 (Exp.).


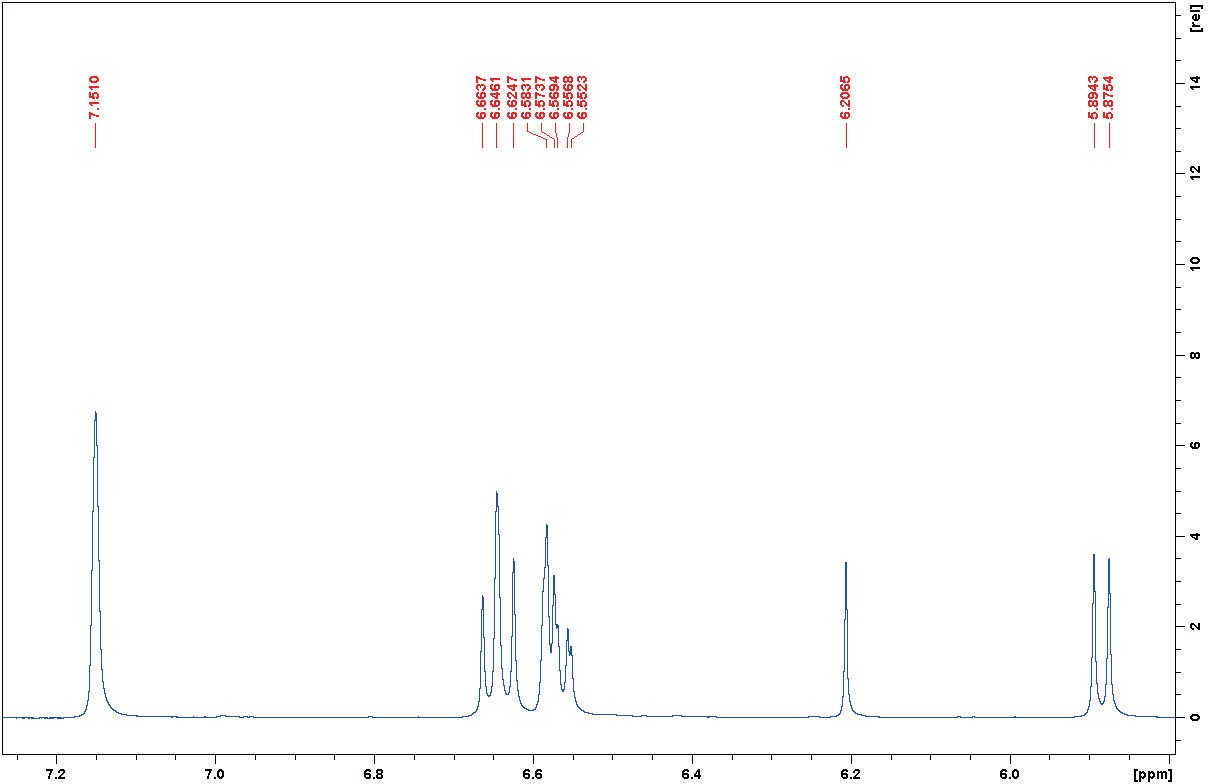


**Figure** **S26**: ^1^HNMR spectrum of **3** in C6D6 (Exp.).


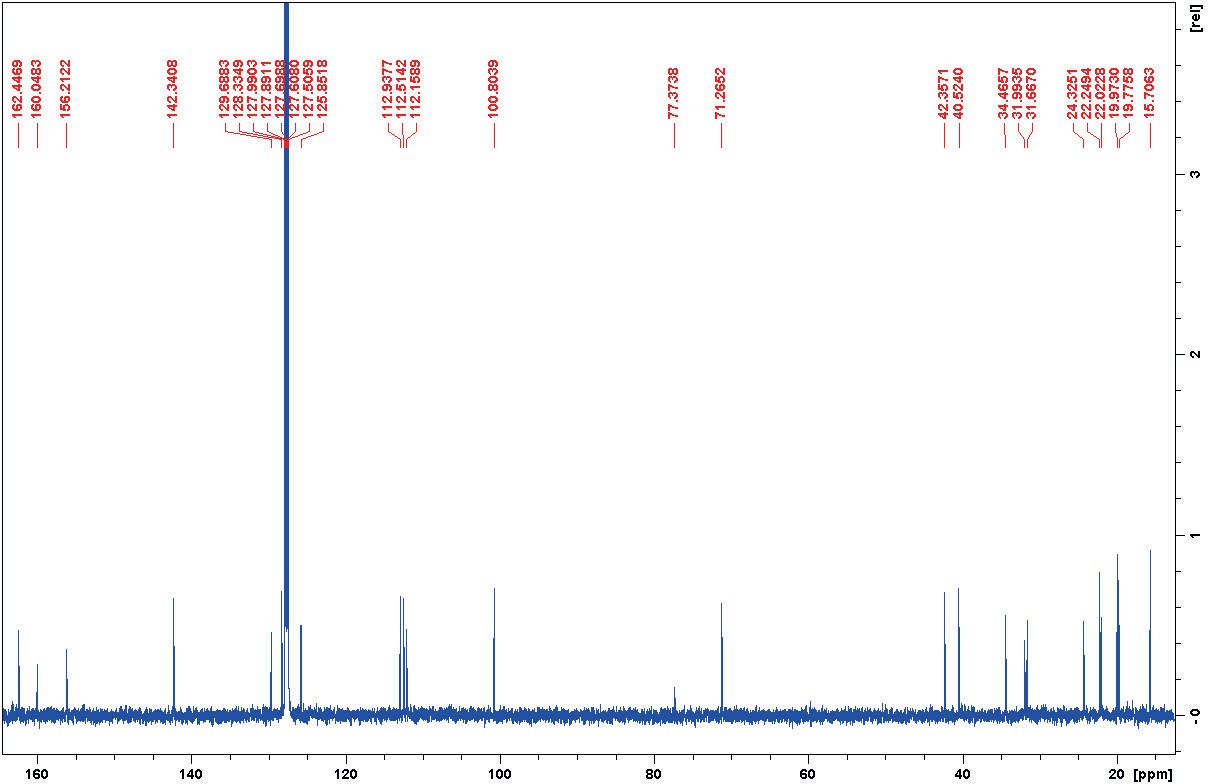


**Figure** **S27**: ^13^CNMR spectrum of **3** in C6D6.


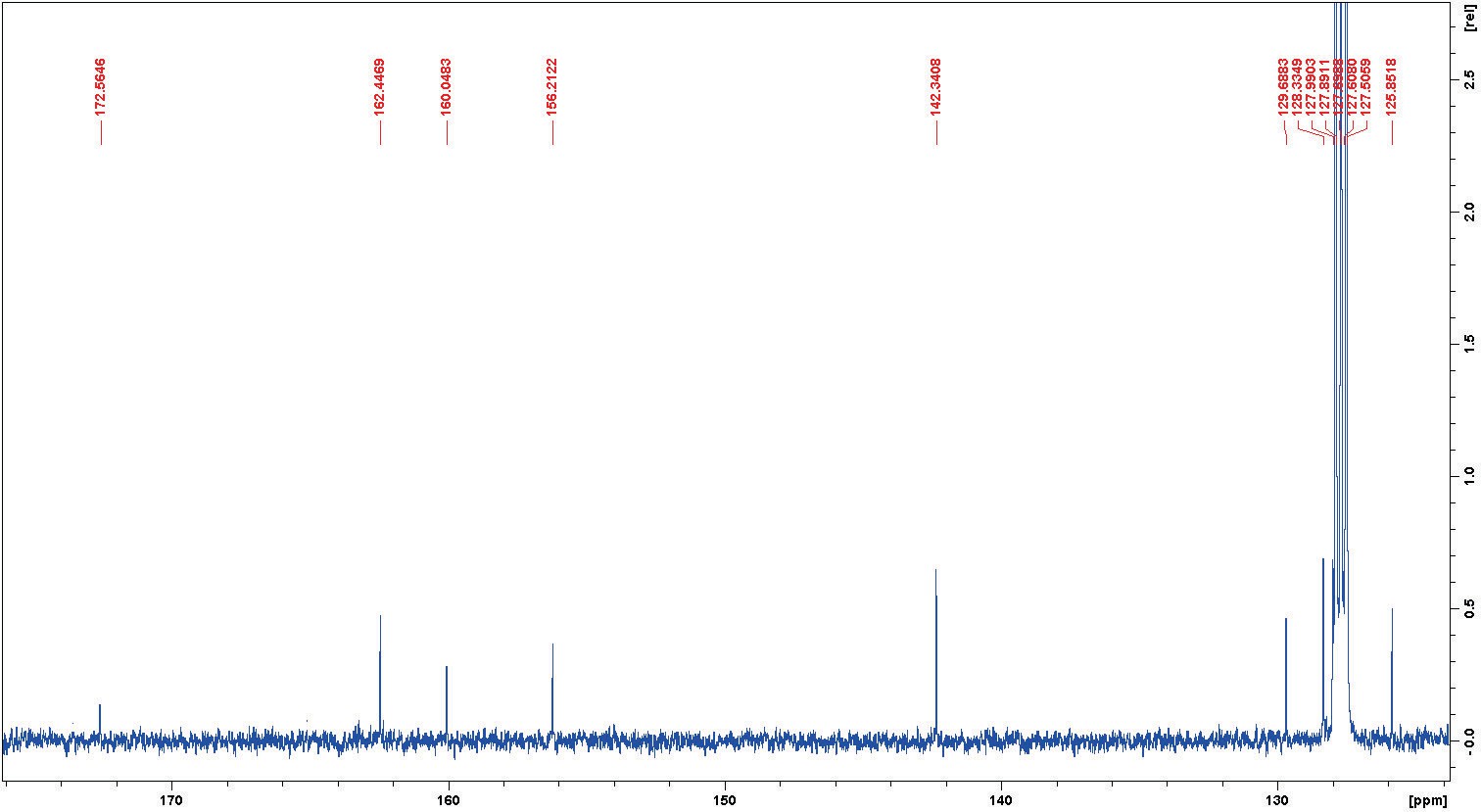


**Figure** **S28**: ^13^CNMR spectrum of **3** in C6D6 (Exp.).


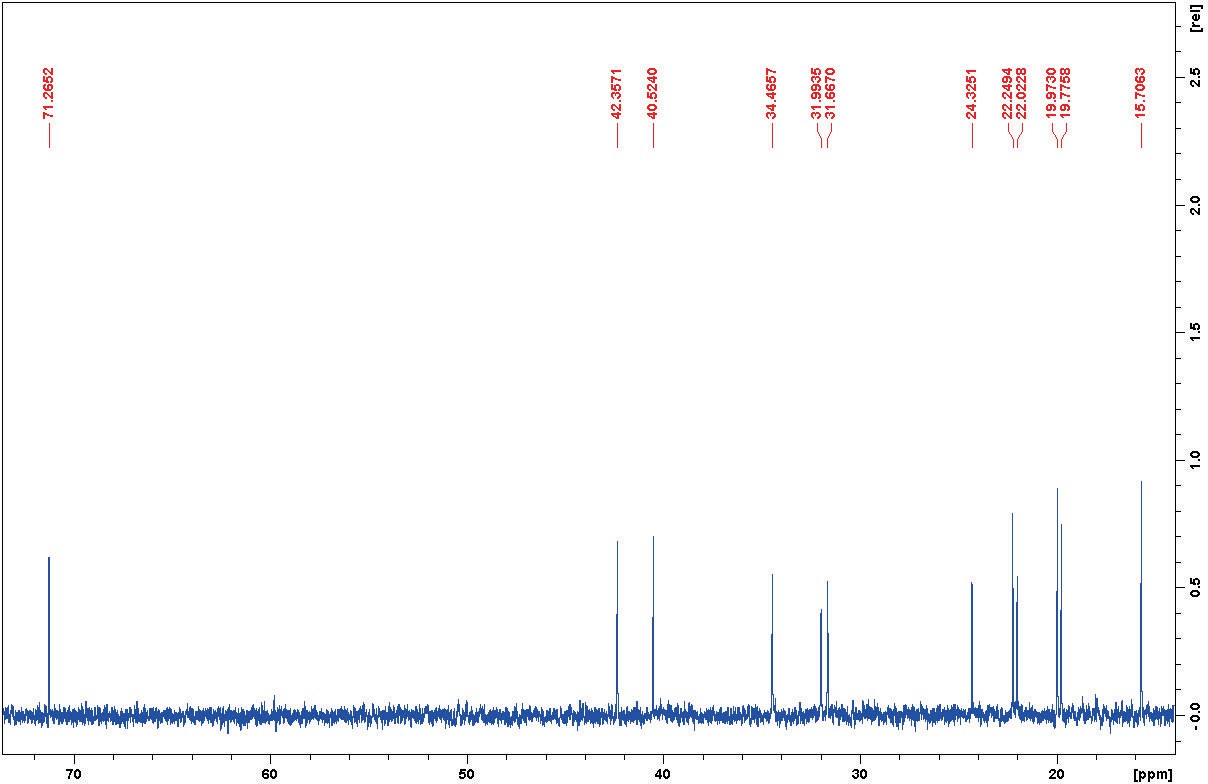


**Figure** **S29**: ^13^CNMR spectrum of **3** in C6D6 (Exp.).


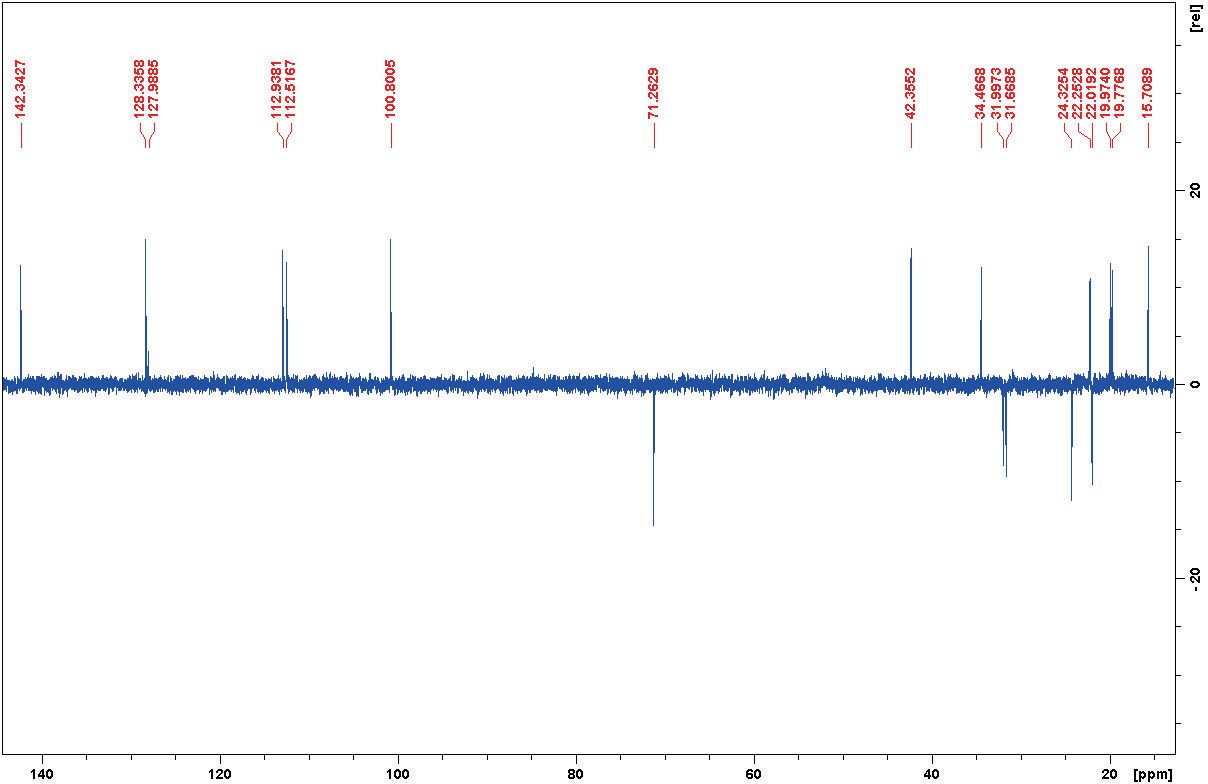


**Figure** **S30**: DEPT135 spectrum of **3** in C6D6.


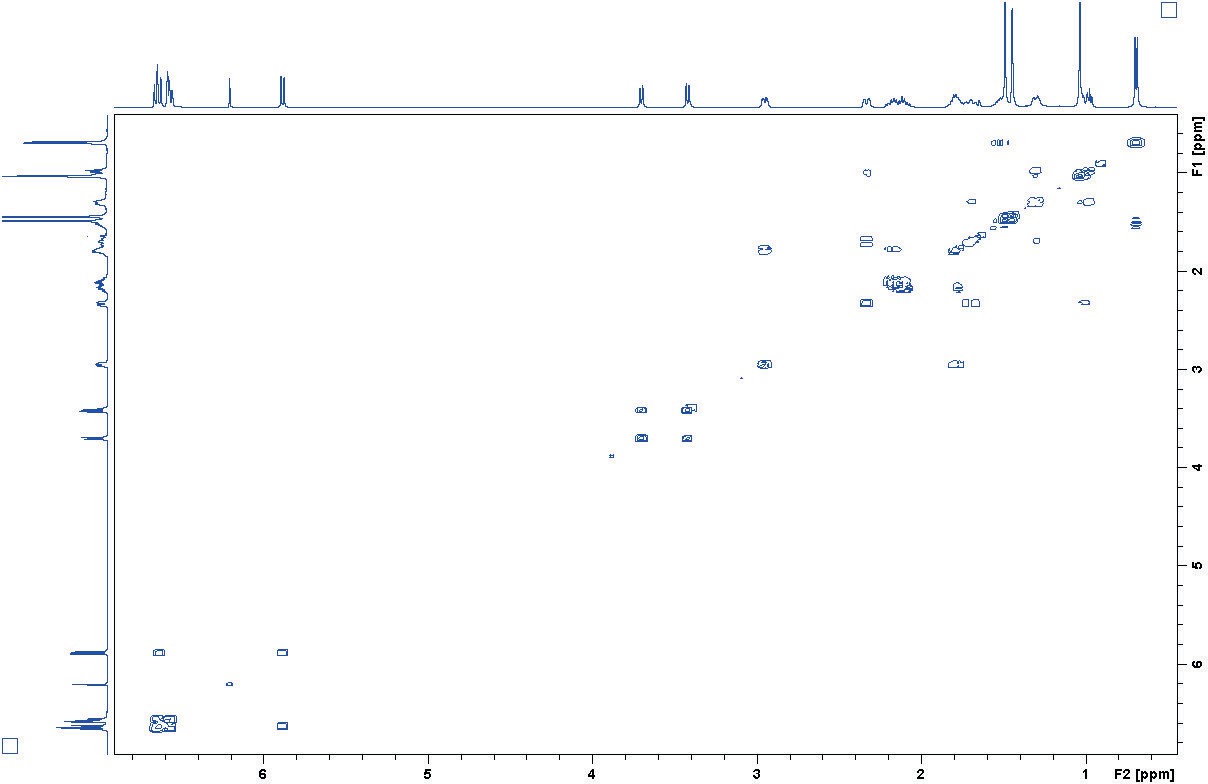


**Figure** **S31**: COSY spectrum of **3** in C6D6.


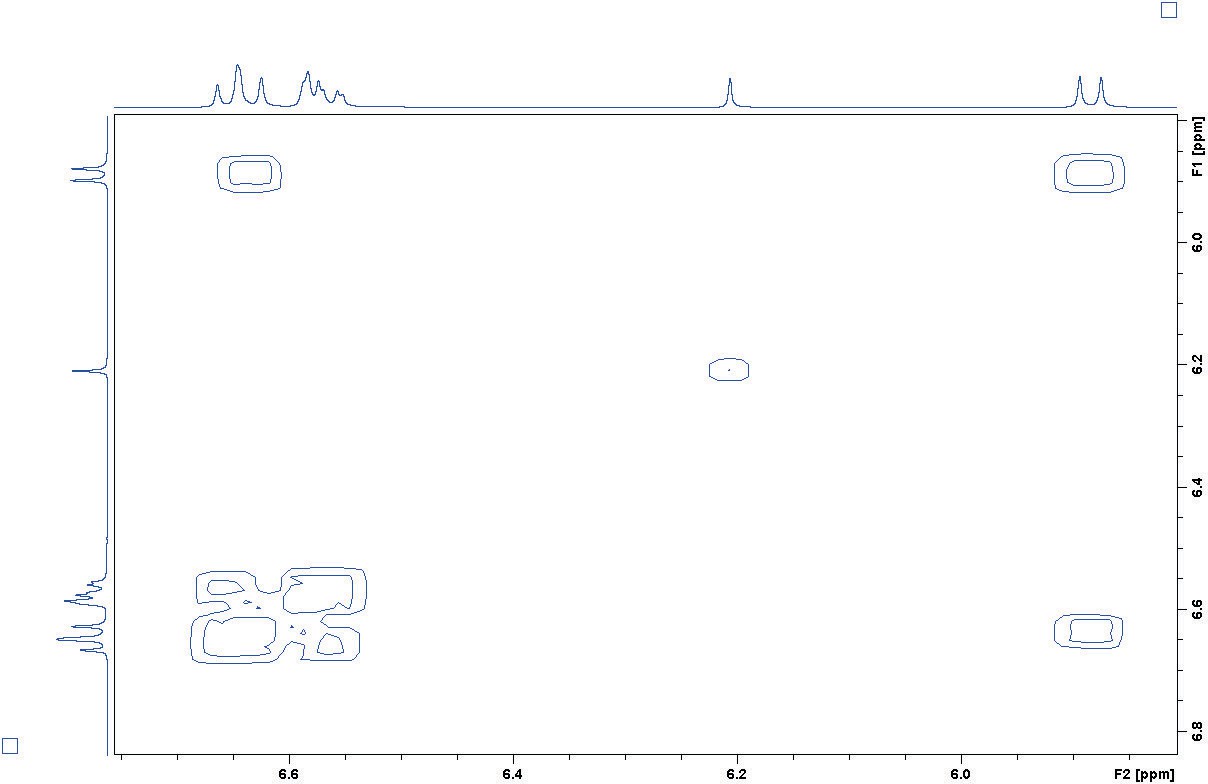


**Figure** **S32**: COSY spectrum of **3** in C6D6 (Exp.).


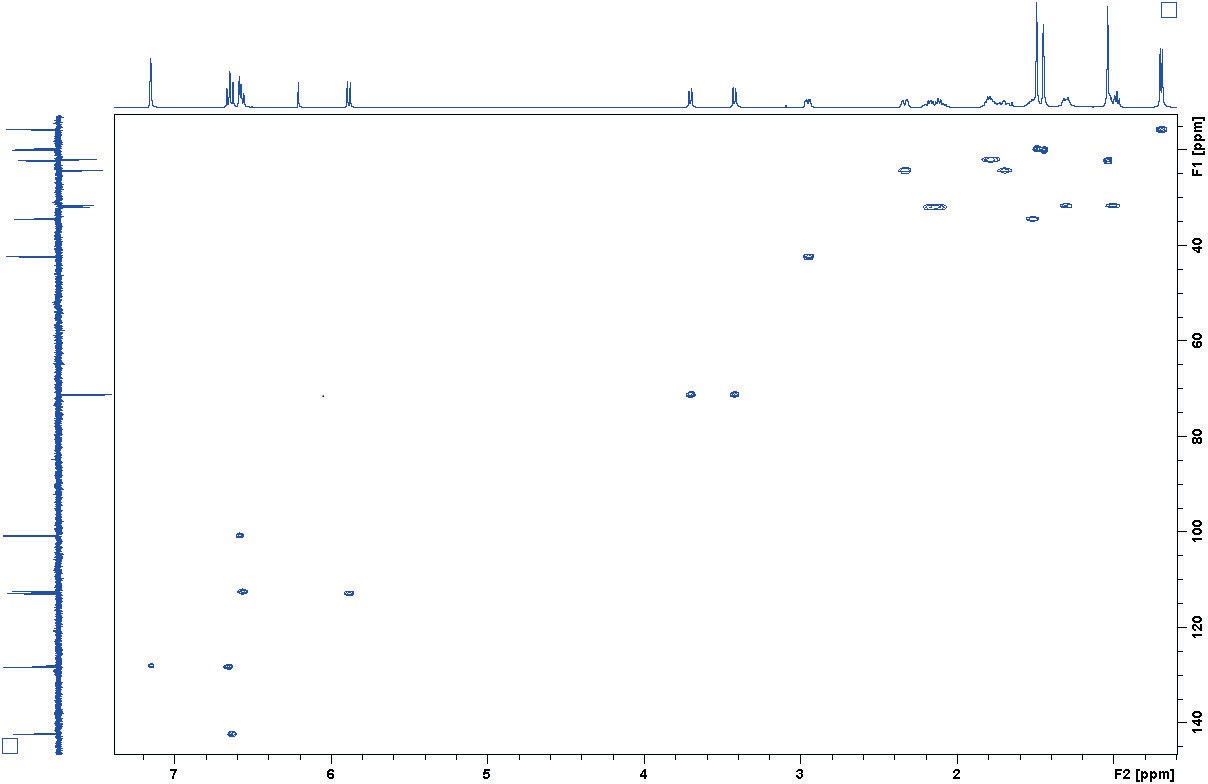


**Figure** **S33**: HSQC spectrum of **3** in C6D6.


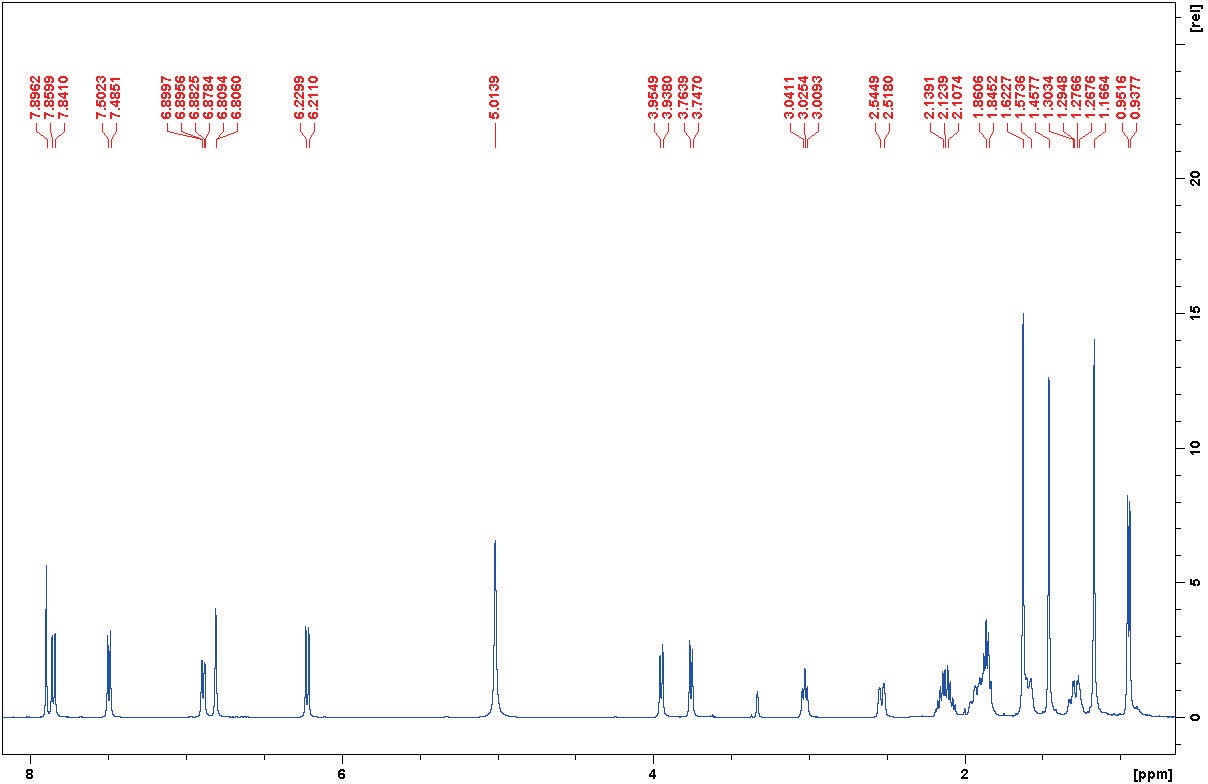


**Figure** **S34**: ^1^HNMR spectrum of **3** in CD3OD.


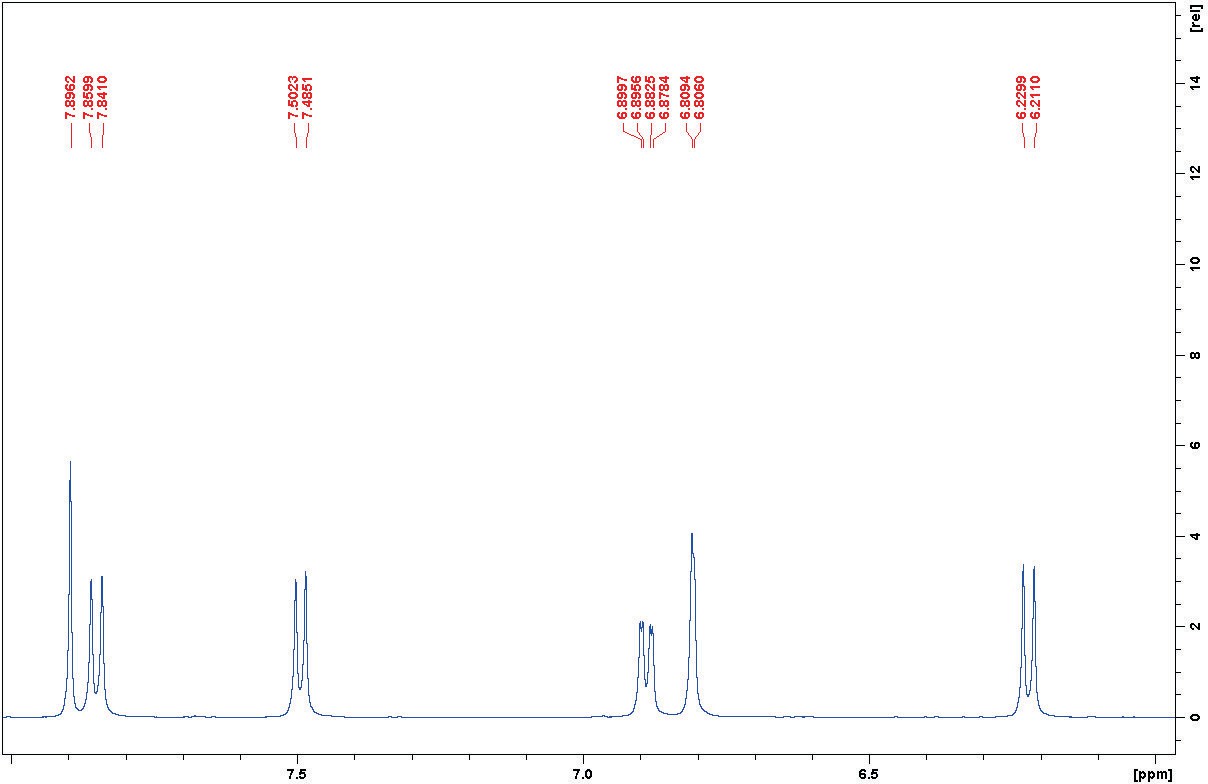


**Figure** **S35**: ^1^HNMR spectrum of **3** in CD3OD (Exp.).


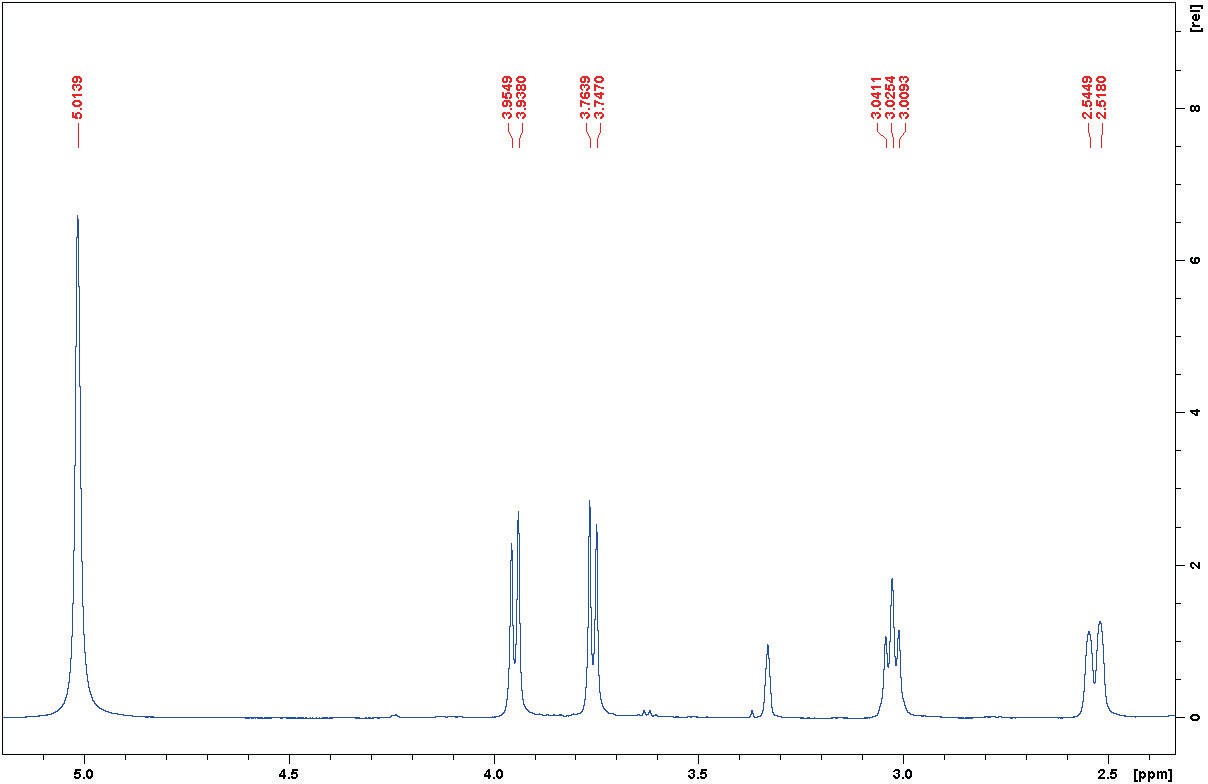


**Figure** **S36**: ^1^HNMR spectrum of **3** in CD3OD (Exp.).


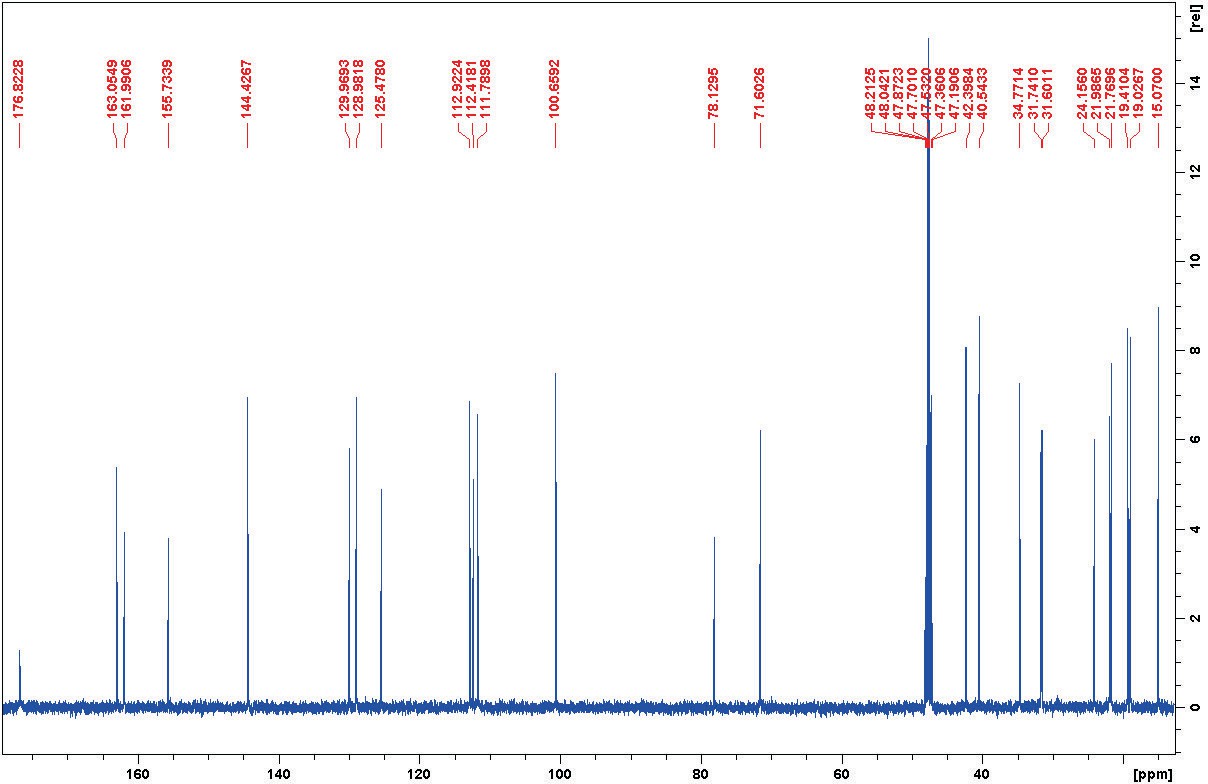


**Figure** **S37**: ^13^CNMR spectrum of **3** in CD3OD.


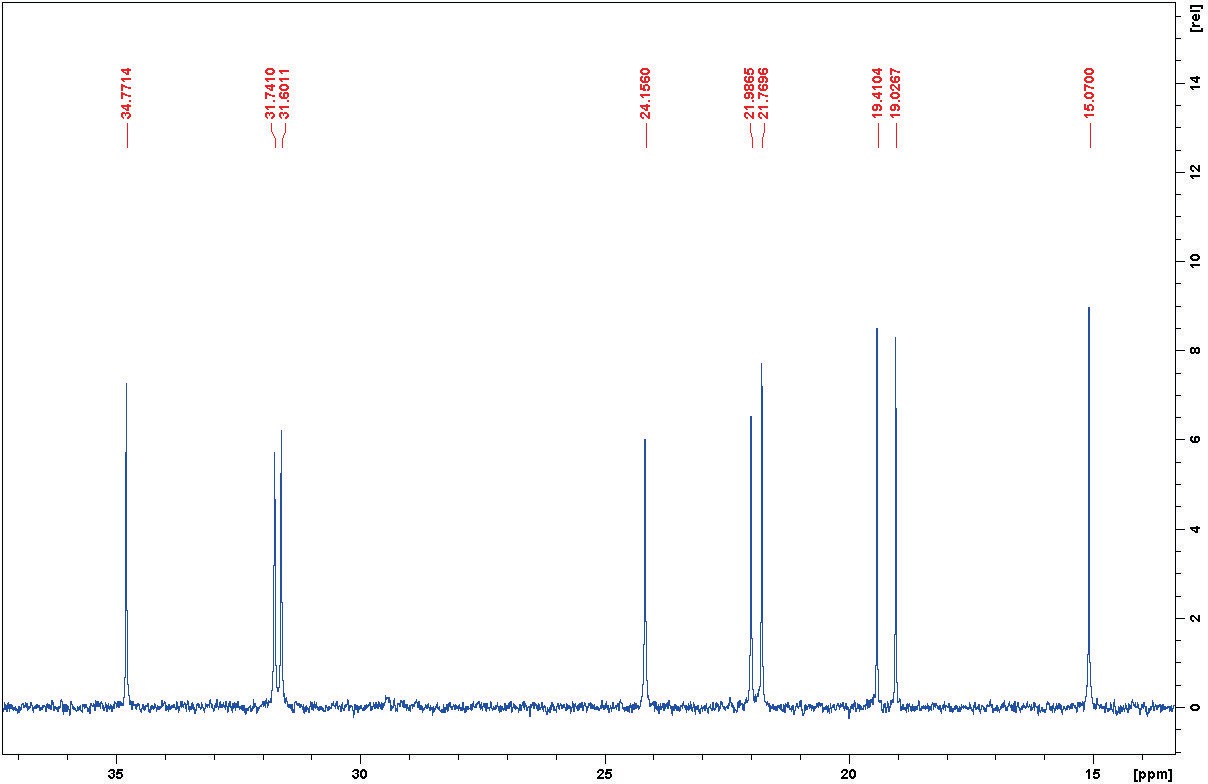


**Figure** **S38**: ^13^CNMR spectrum of **3** in CD3OD (Exp.).


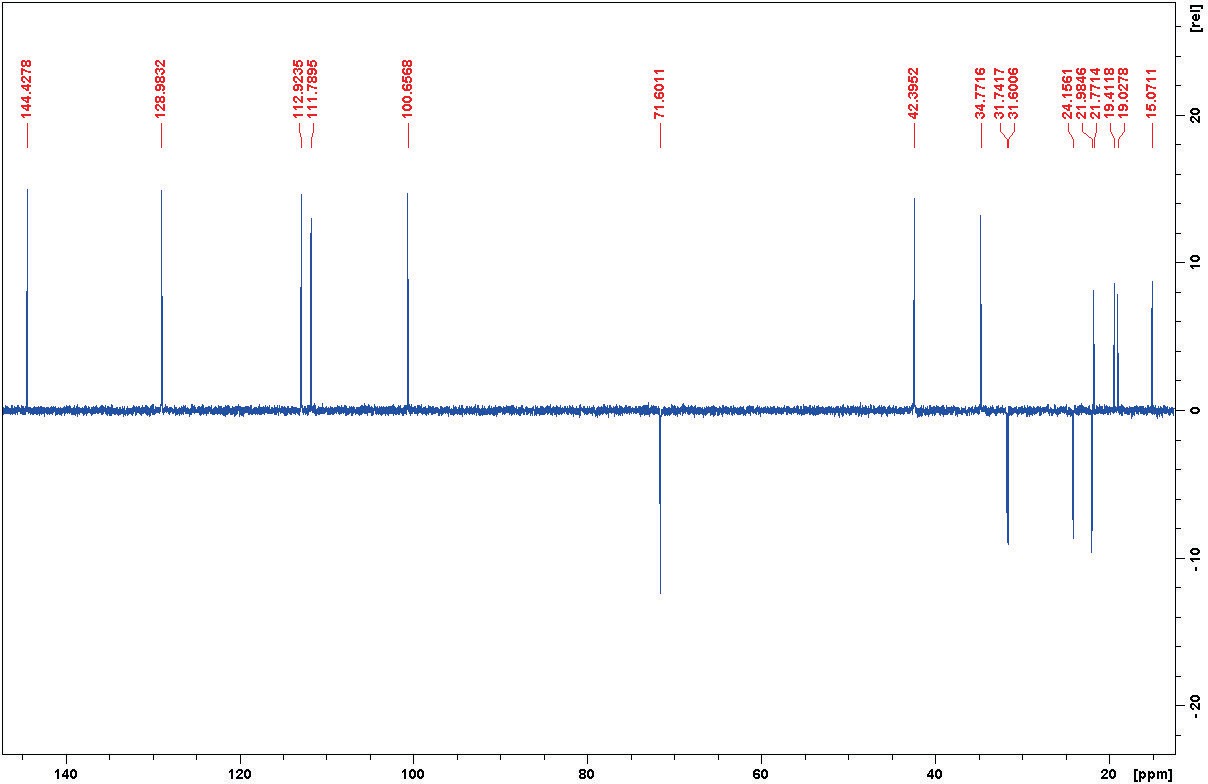


**Figure** **S39**: DEPT135 spectrum of **3** in CD3OD.


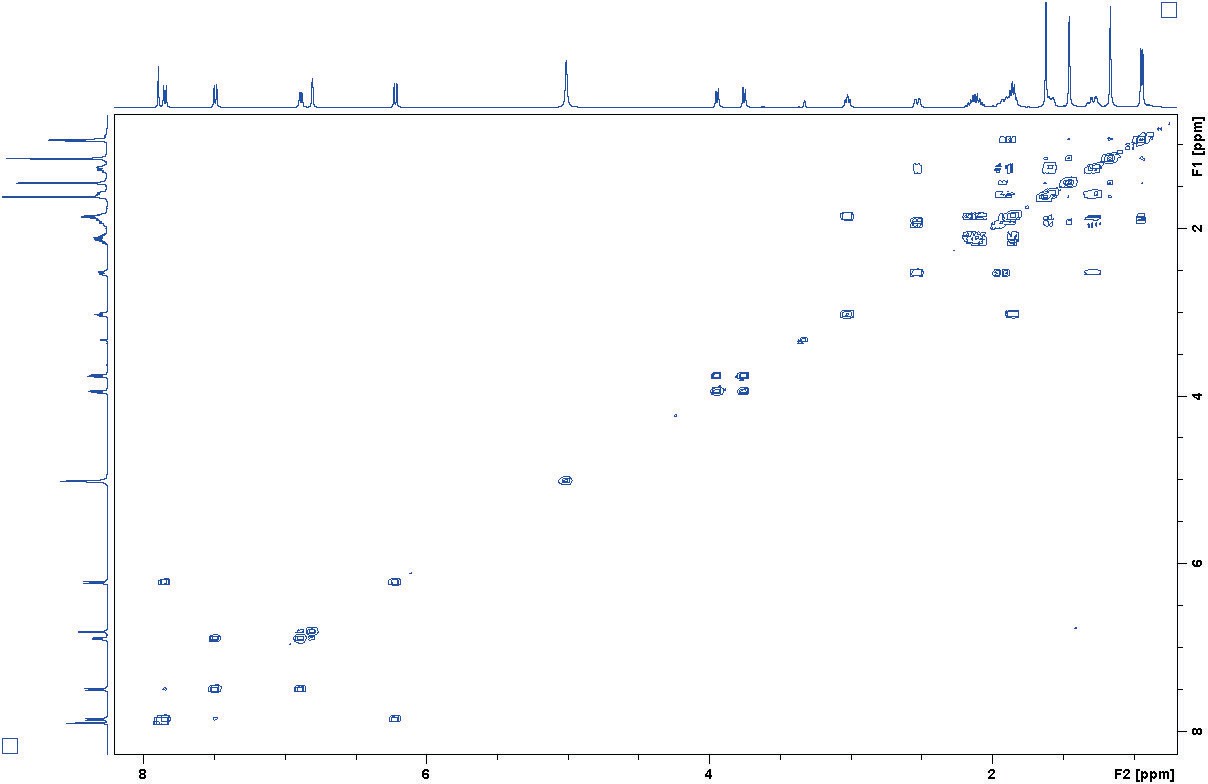


**Figure** **S40**: COSY spectrum of **3** in CD3OD.


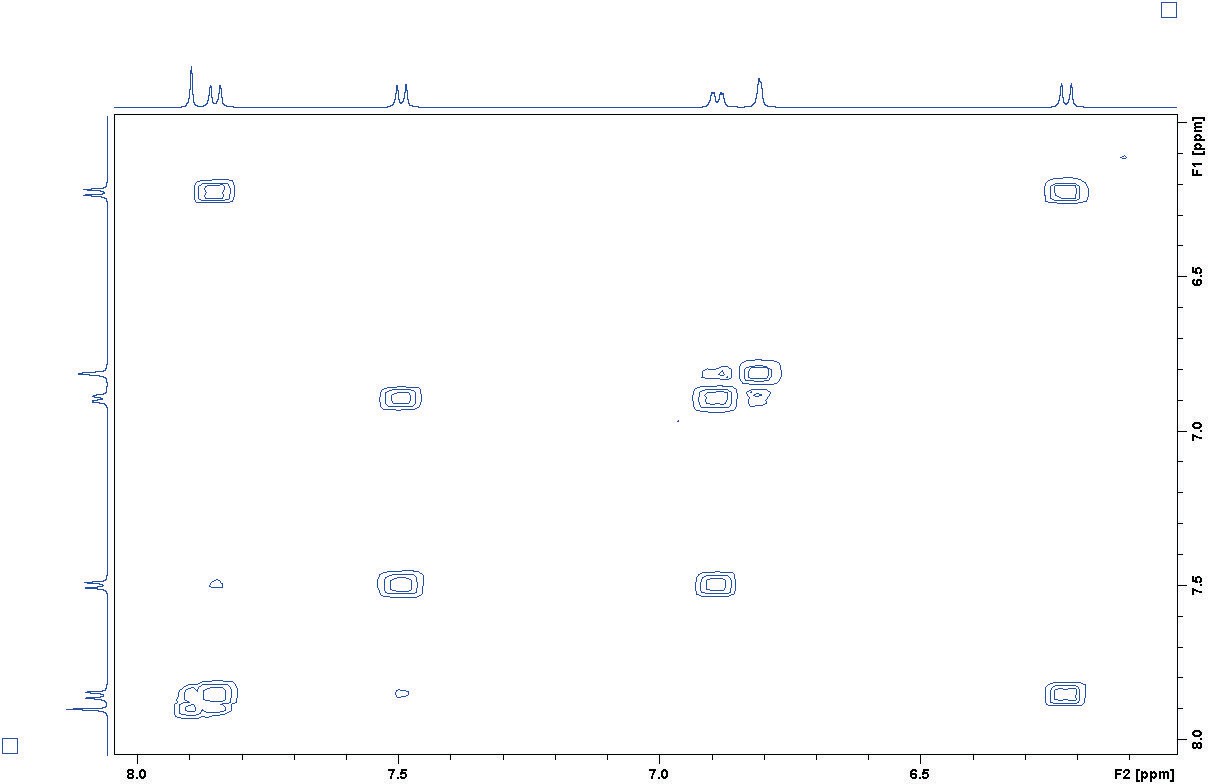


**Figure** **S41**: COSY spectrum of **3** in CD3OD (Exp.).


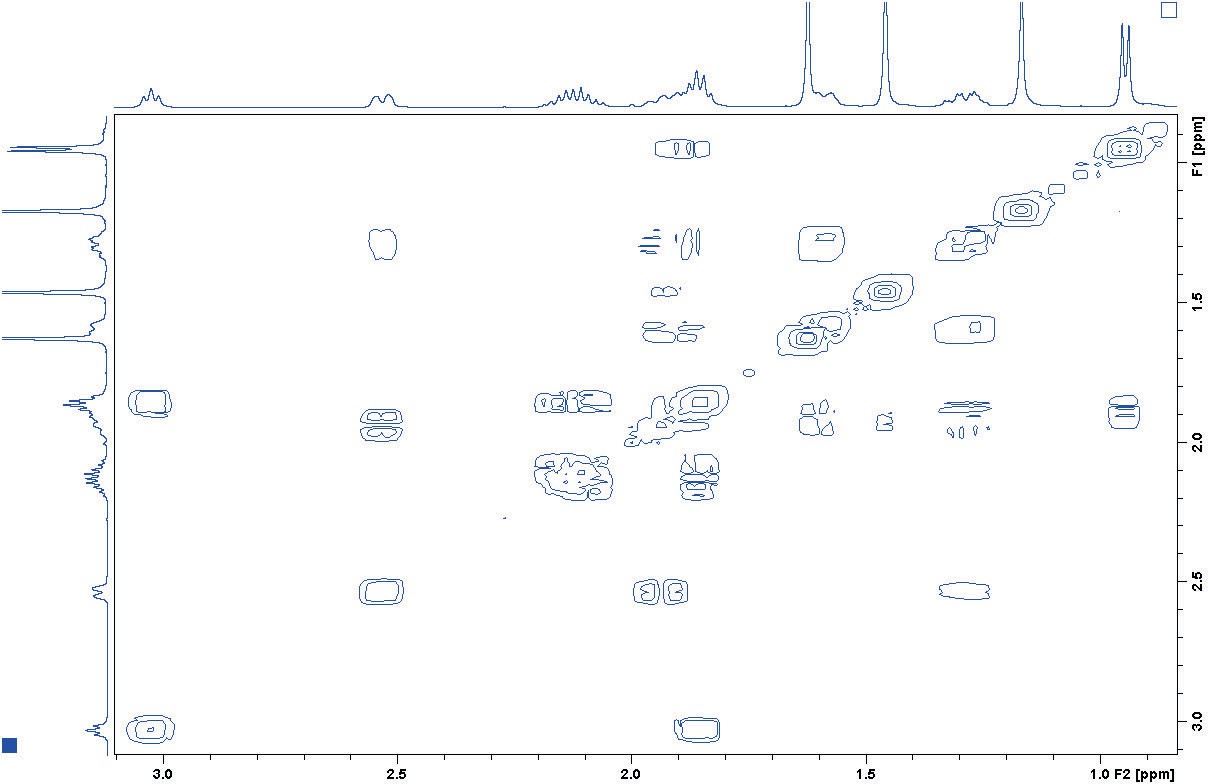


**Figure** **S42**: COSY spectrum of **3** in CD3OD (Exp.).


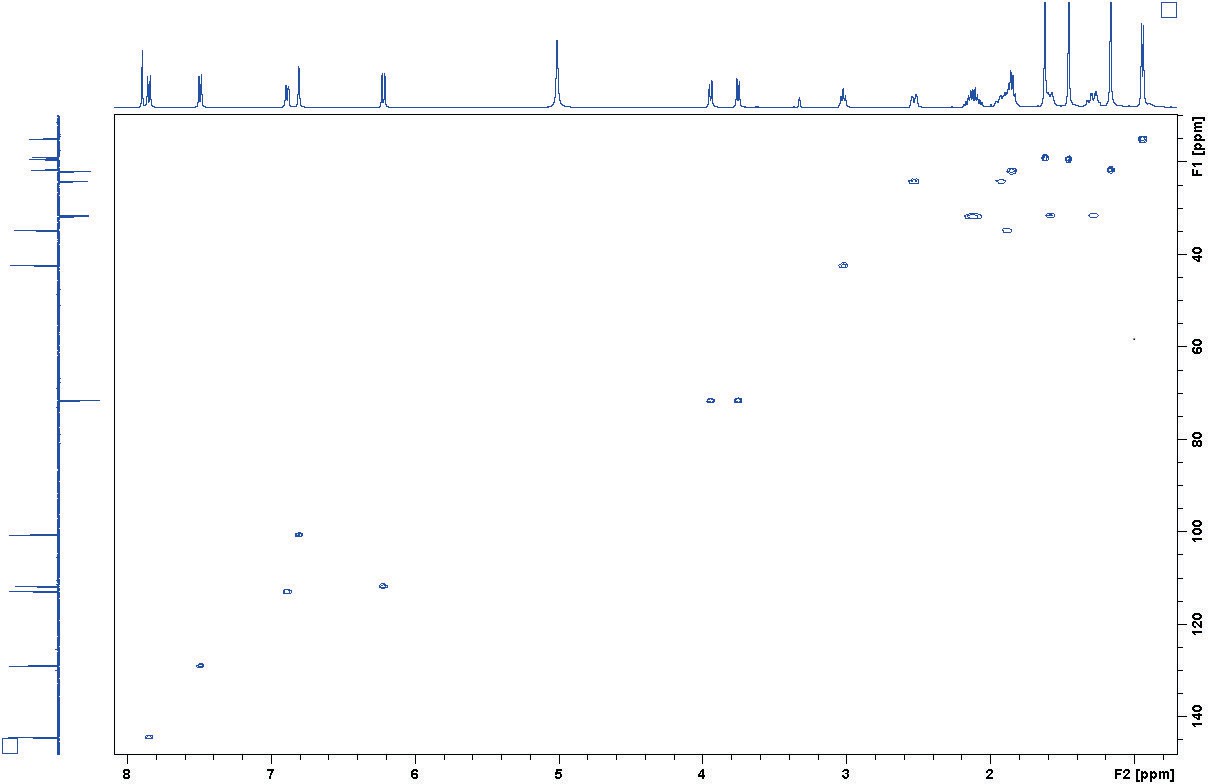


**Figure** **S43**: HSQC spectrum of **3** in CD3OD.


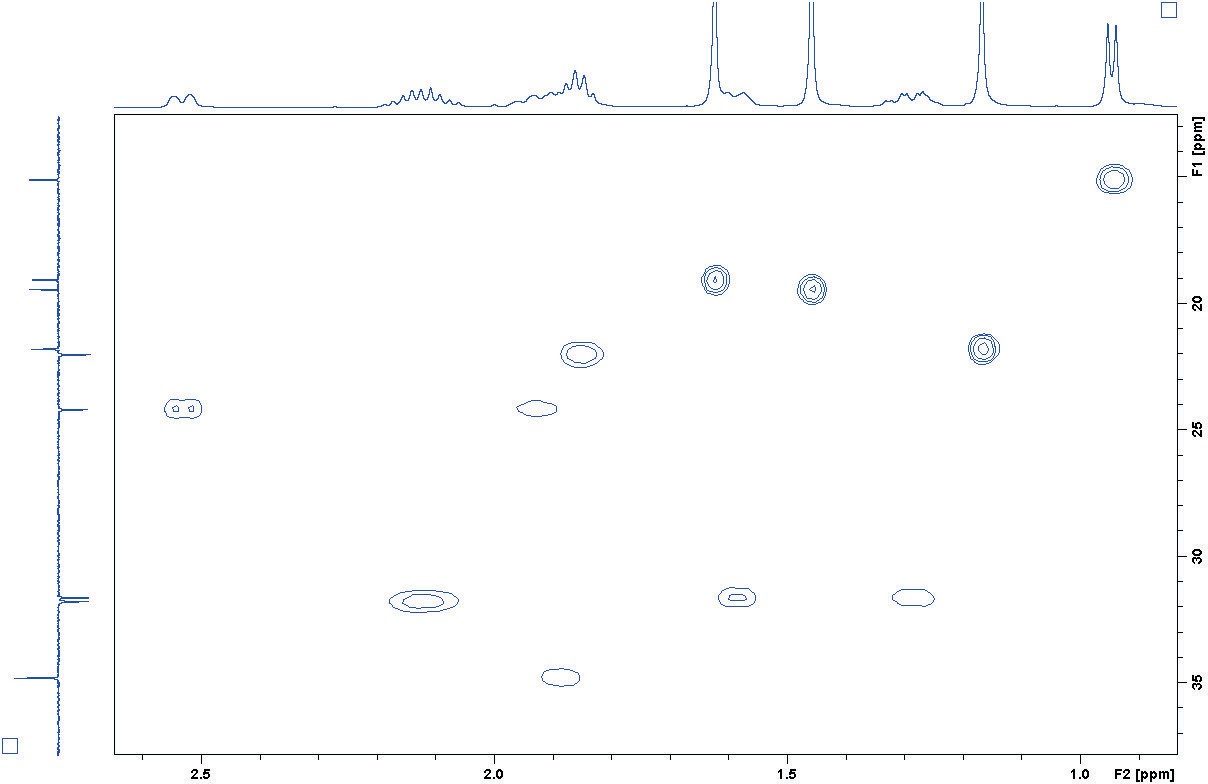


**Figure** **S44**: HSQC spectrum of **3** in CD3OD (Exp.).


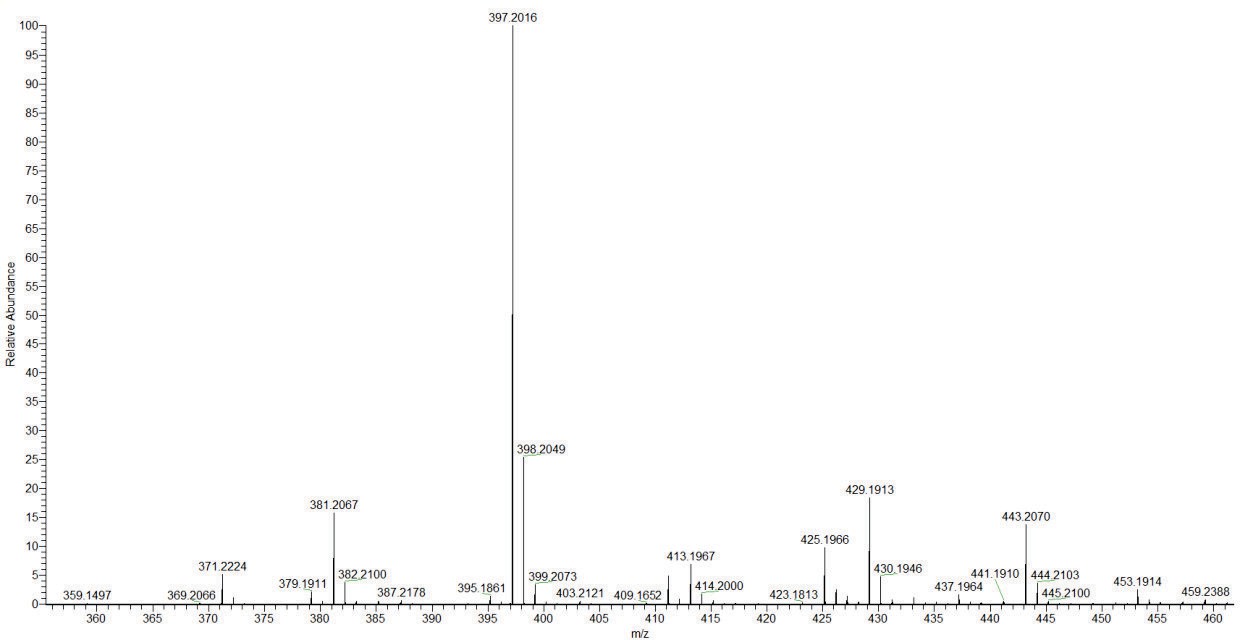


# **Figure** **S45**: HRESIMS spectrum of **3** (Negative mode).


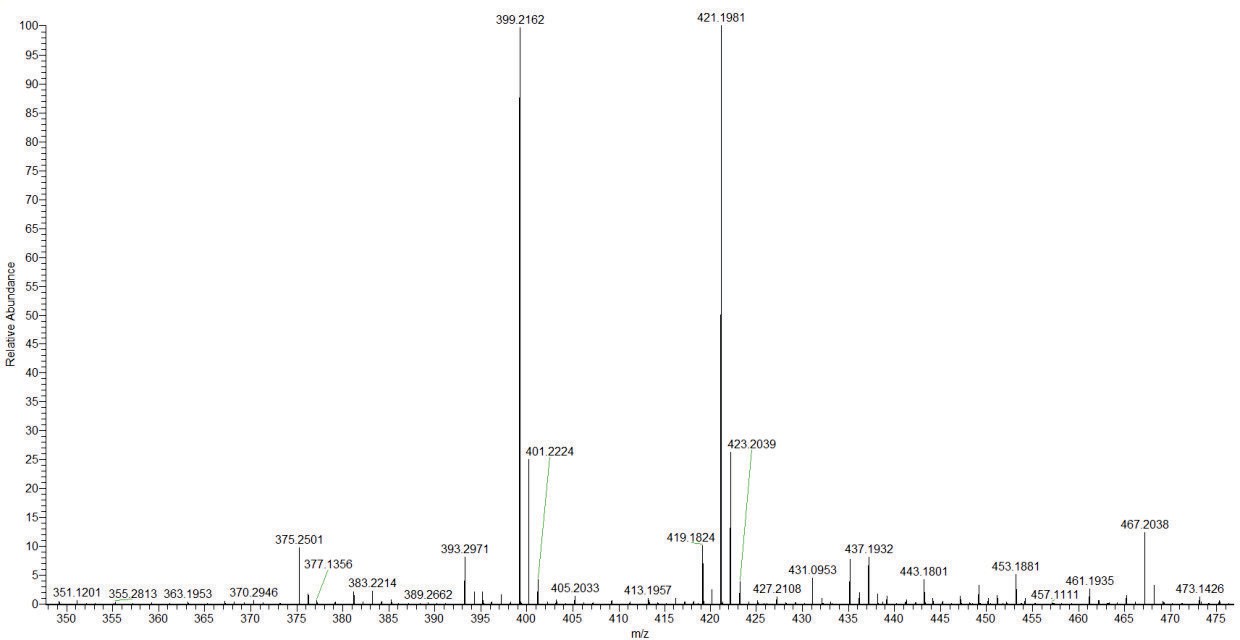


**Figure** **S46**: HRESIMS spectrum of **3** (Positive mode).


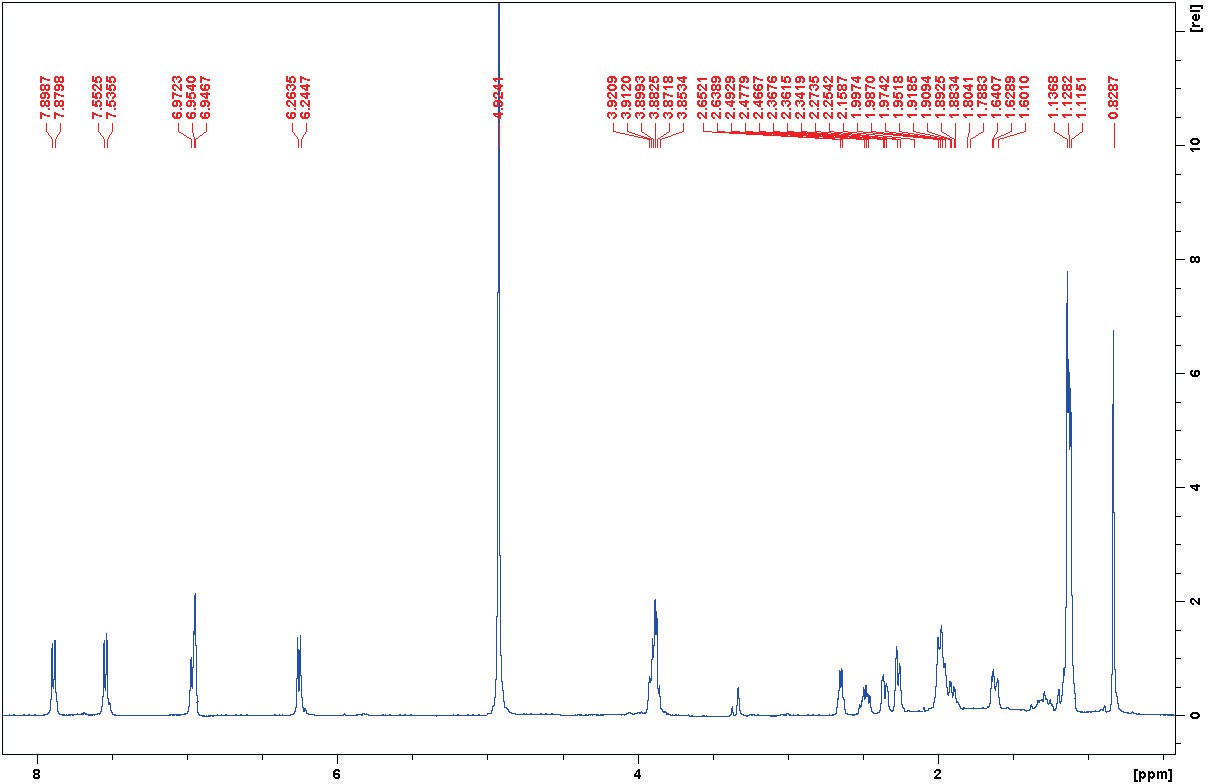


**Figure** **S47**: ^1^HNMR spectrum of **4** in CD3OD.


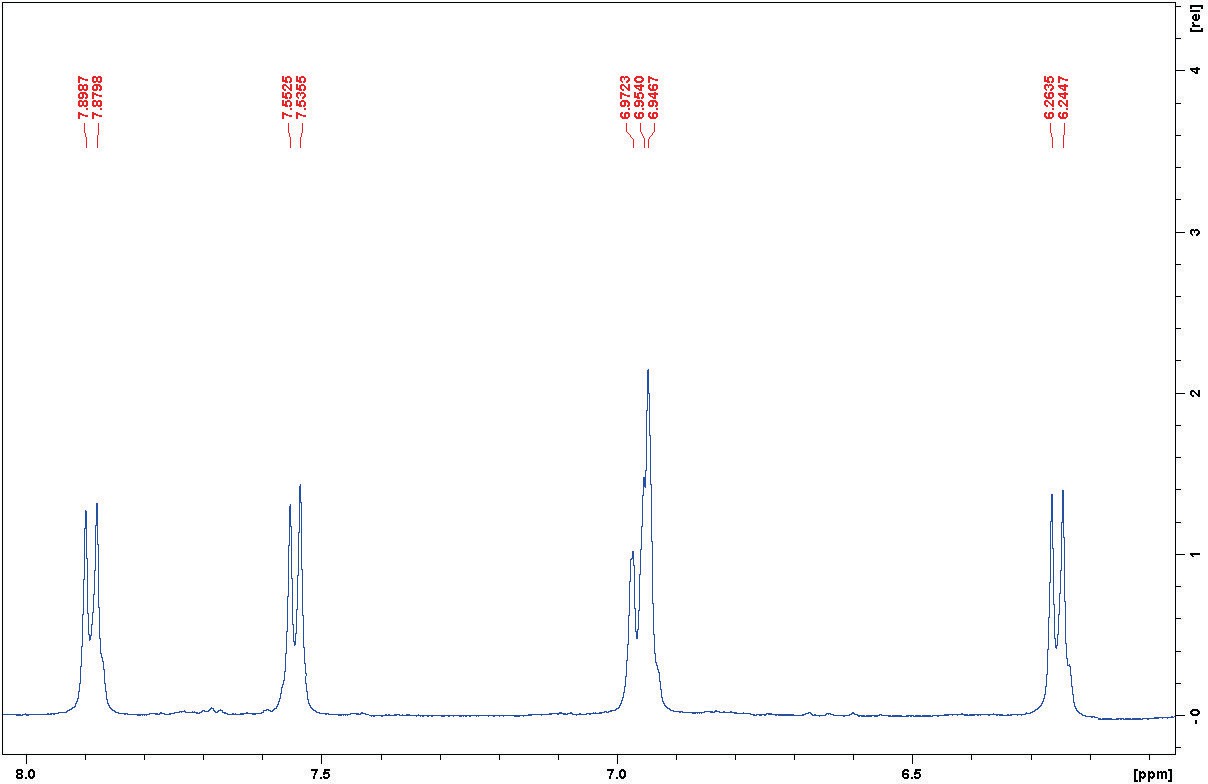


**Figure** **S48**: ^1^HNMR spectrum of **4** in CD3OD (Exp.).


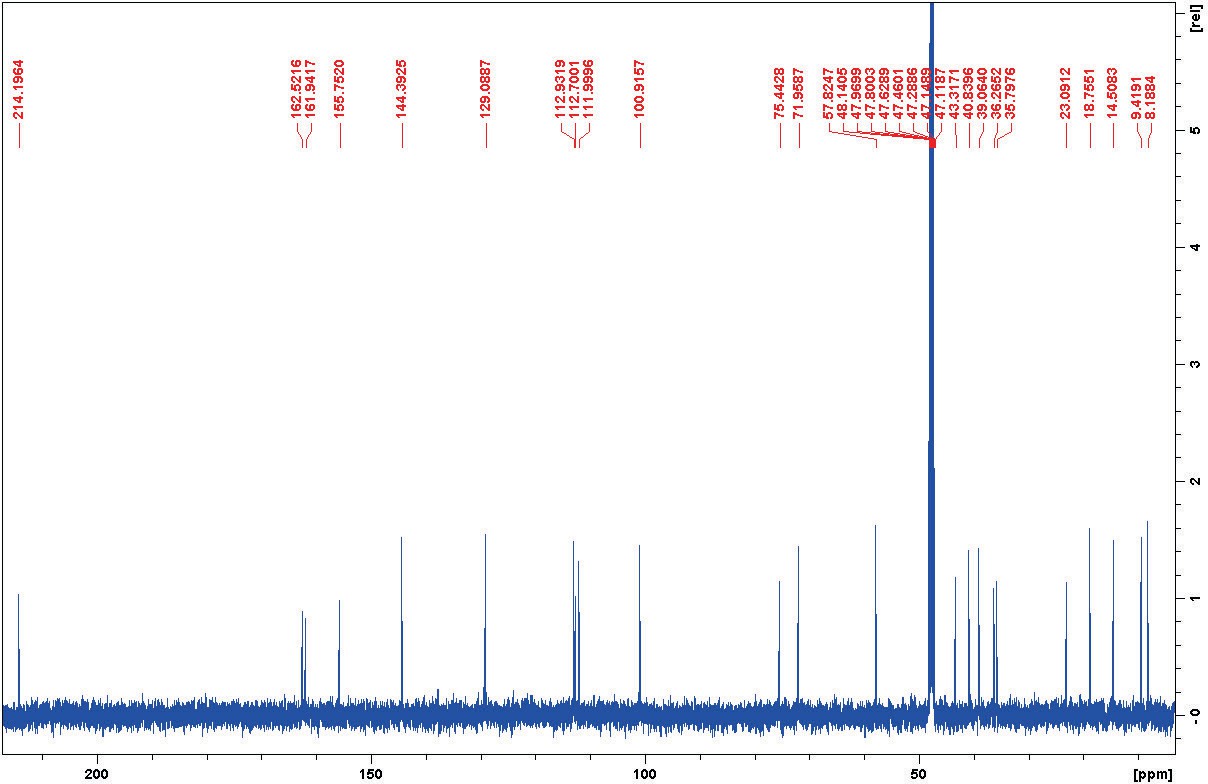


**Figure** **S49**: ^13^CNMR spectrum of **4** in CD3OD.


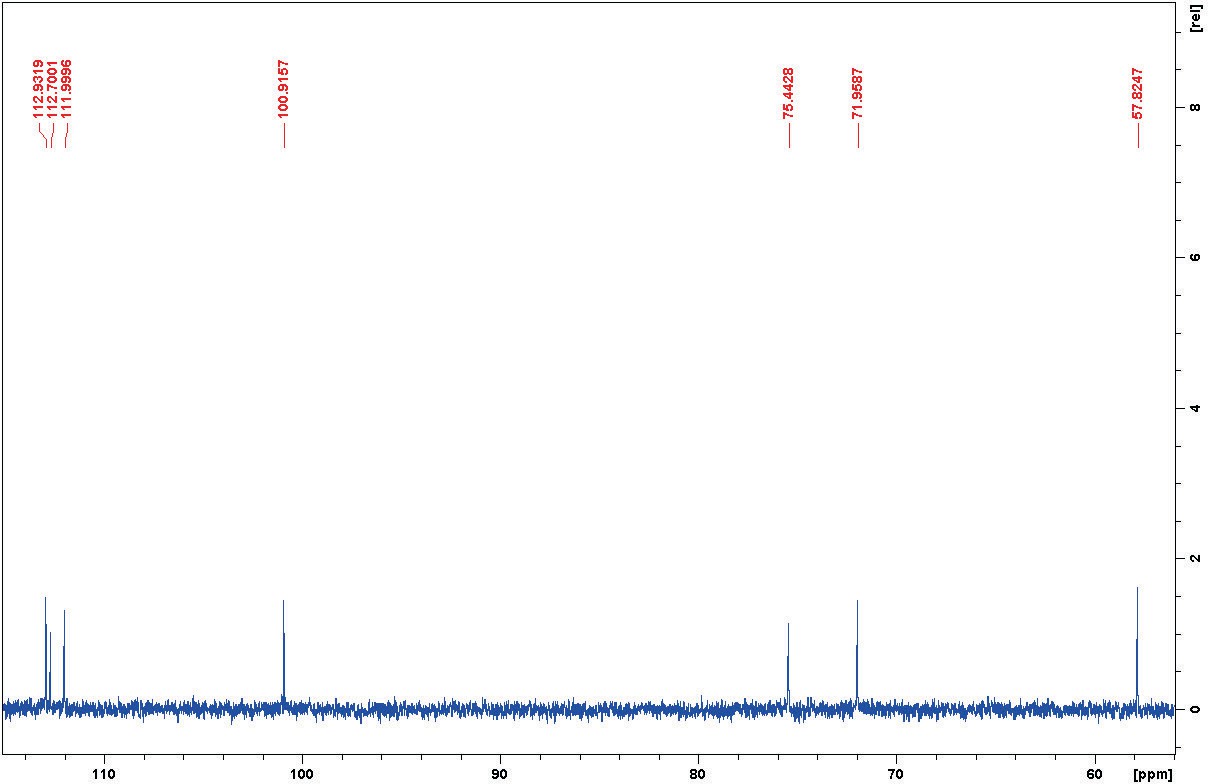


**Figure** **S50**: ^13^CNMR spectrum of **4** in CD3OD (Exp.).


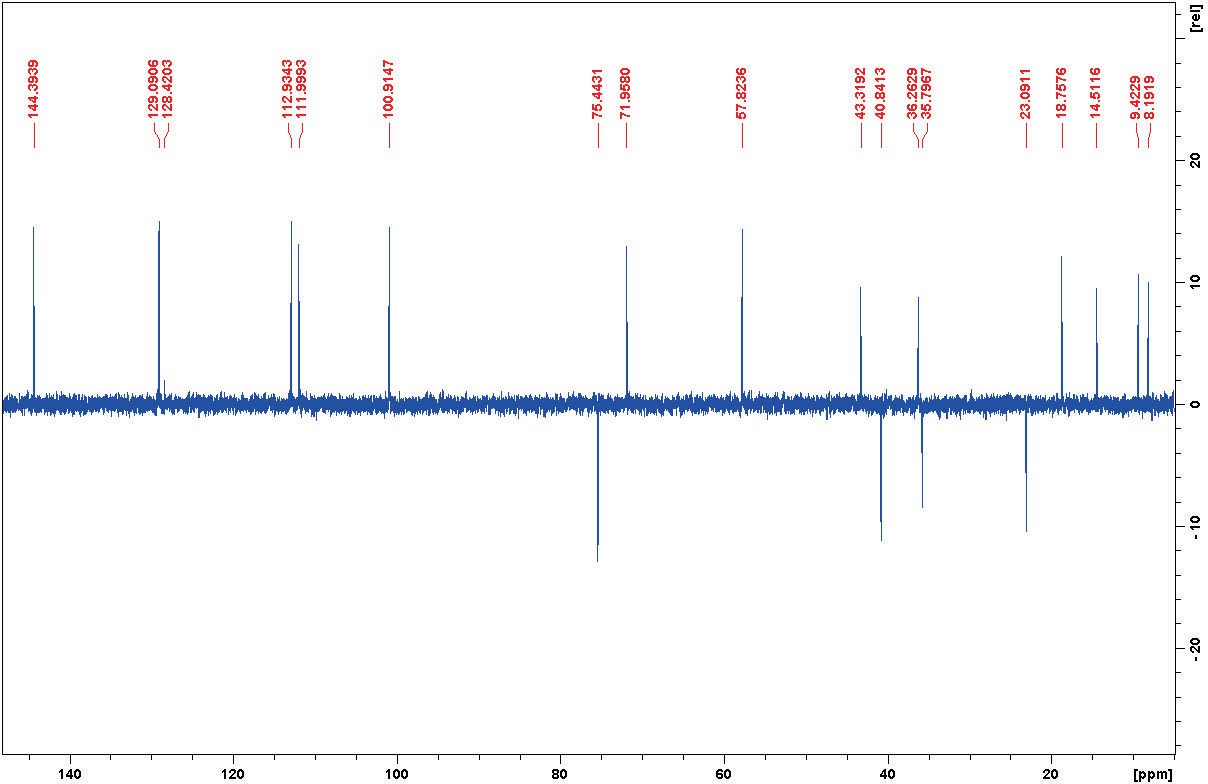


**Figure** **S51**: DEPT135 spectrum of **4** in CD3OD.


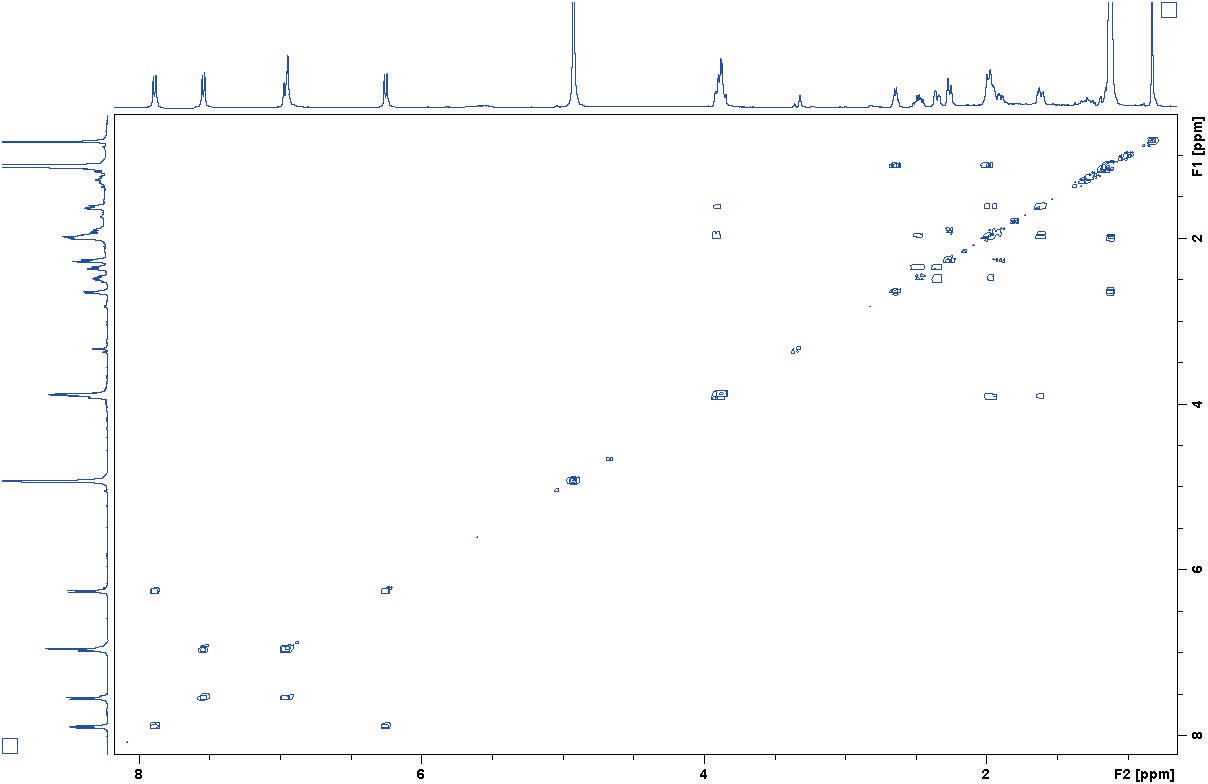


**Figure** **S52**: COSY spectrum of **4** in CD3OD.


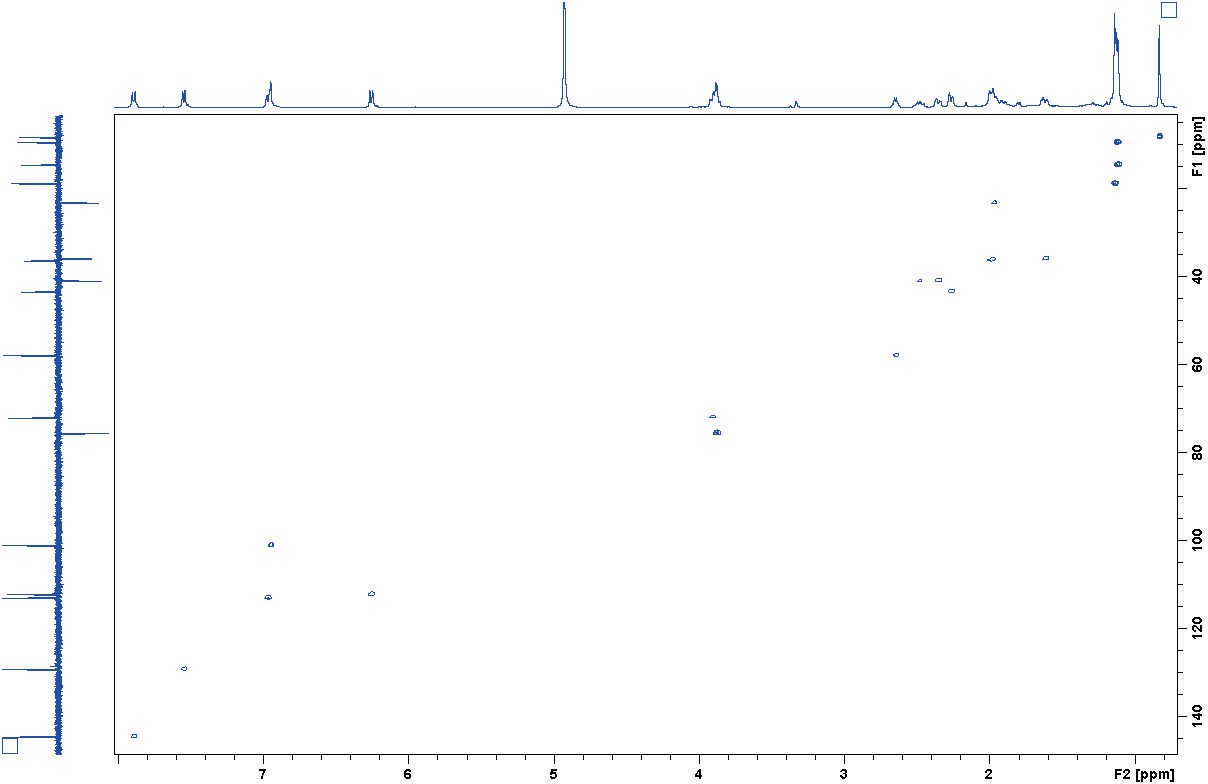


**Figure** **S53**: HSQC spectrum of **4** in CD3OD.


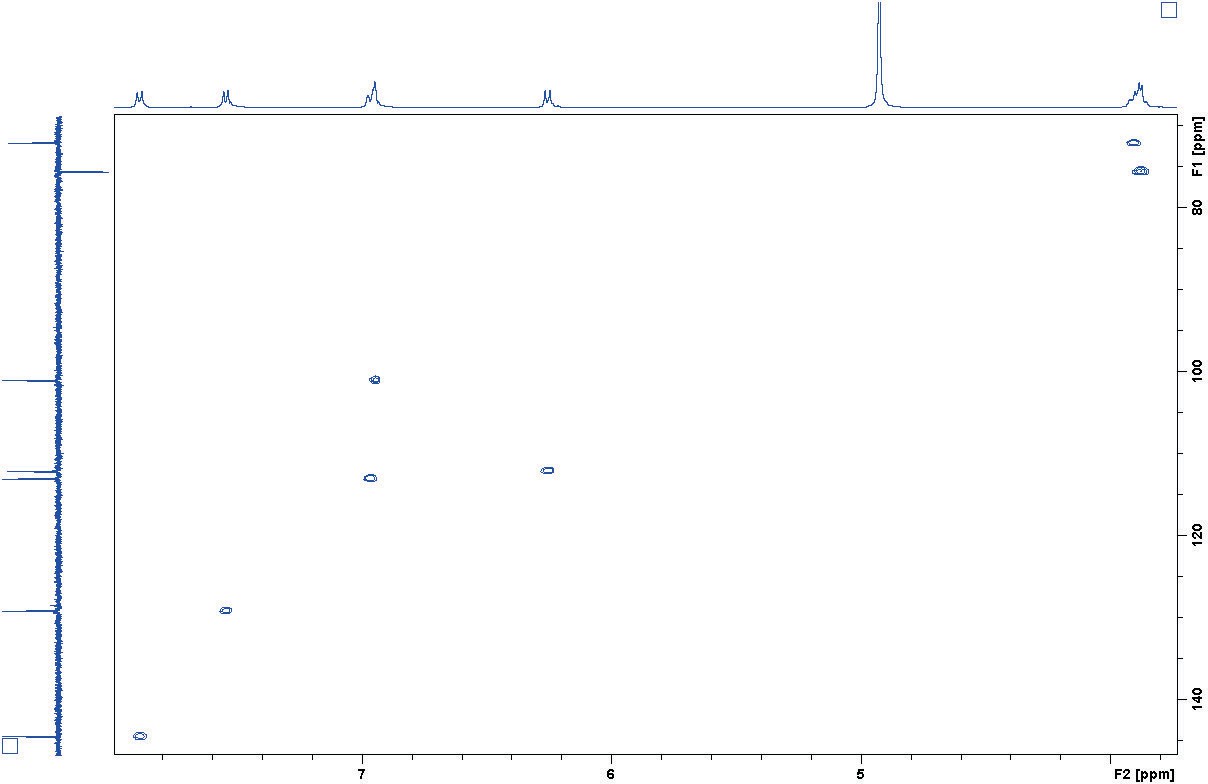


**Figure** **S54**: HSQC spectrum of **4** in CD3OD (Exp.).


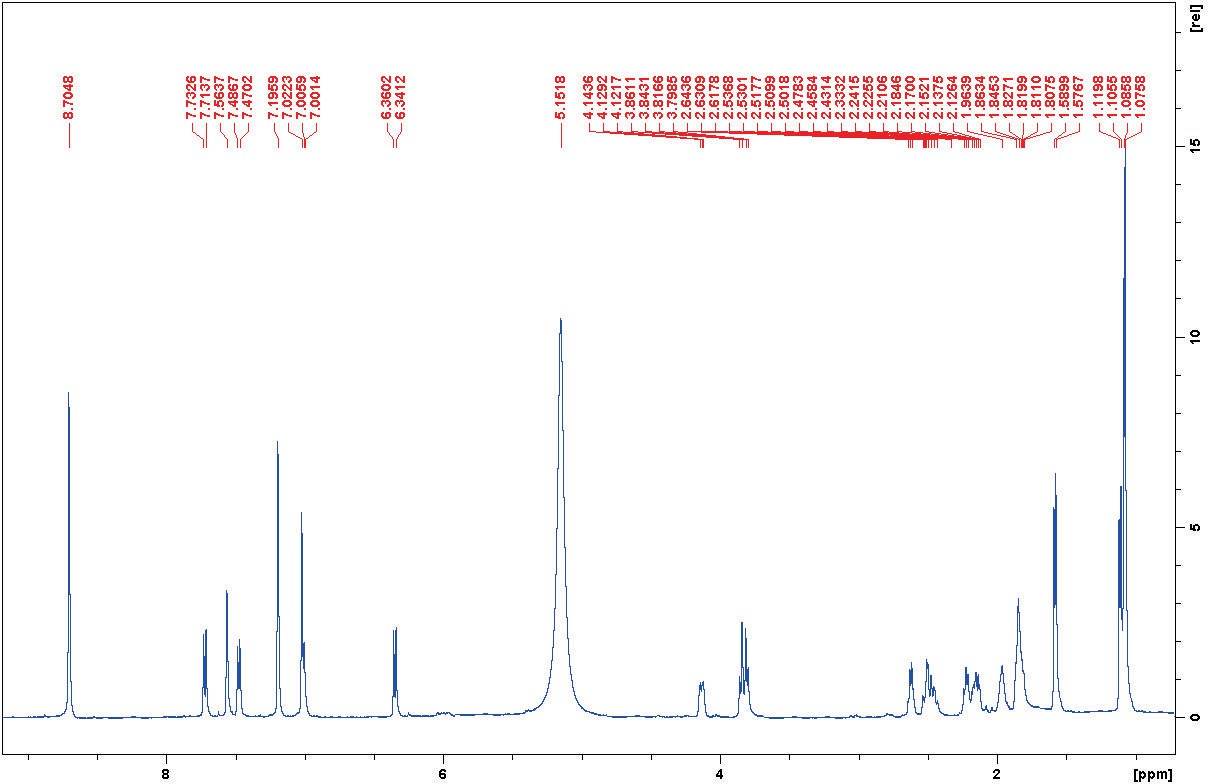


**Figure** **S55**: ^1^HNMR spectrum of **4** in Pyridine d5.


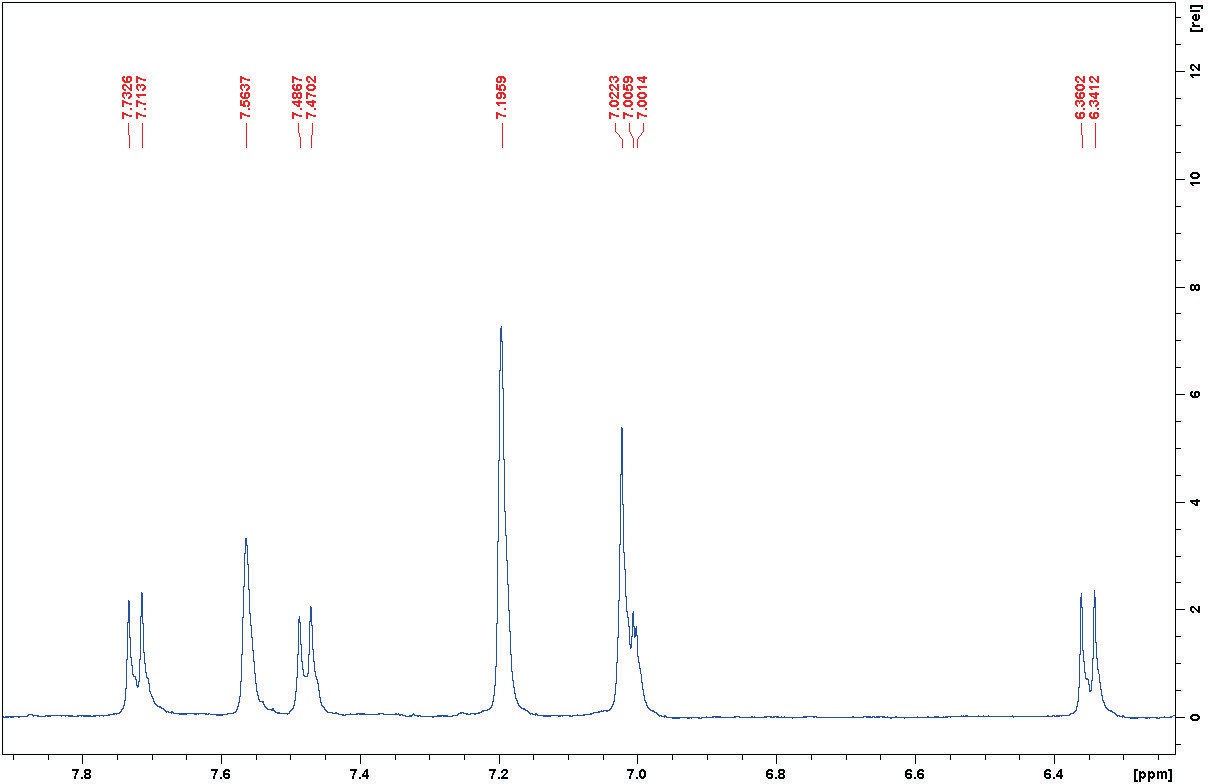


**Figure** **S56**: ^1^HNMR spectrum of **4** in Pyridine d5 (Exp.).


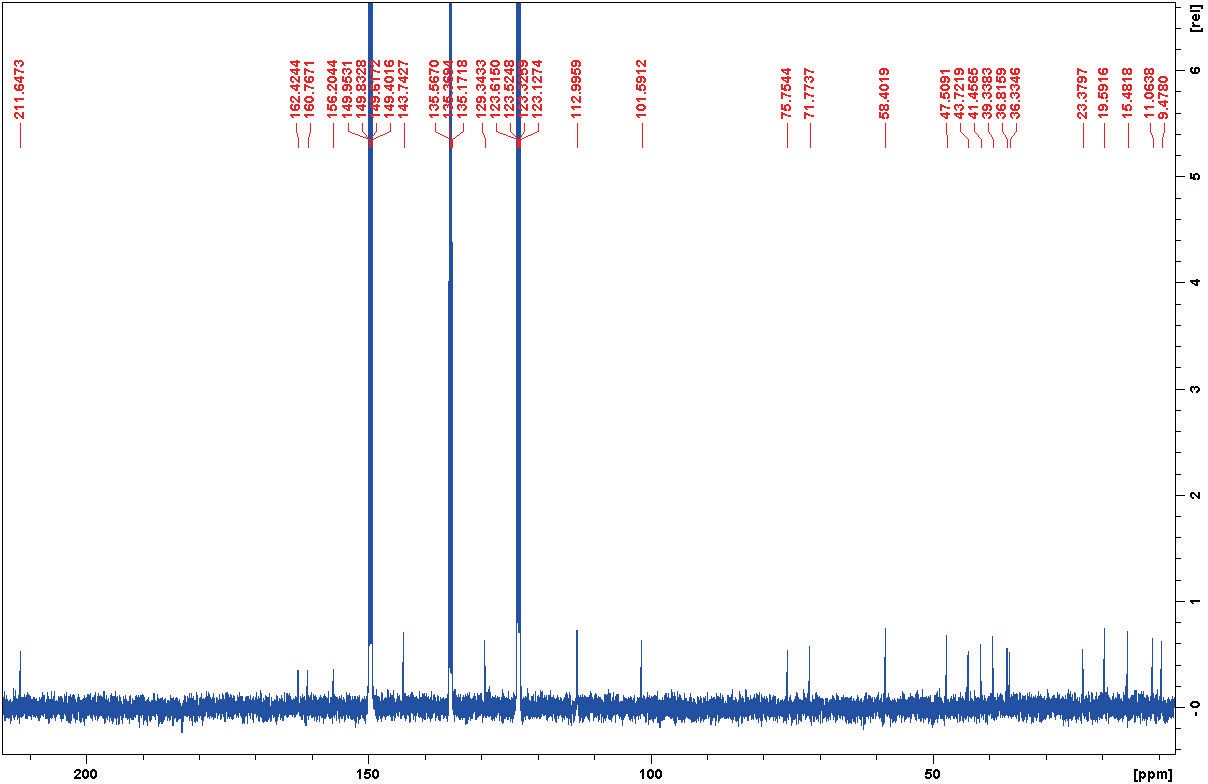


**Figure** **S57**: ^13^CNMR spectrum of **4** in Pyridine d5.


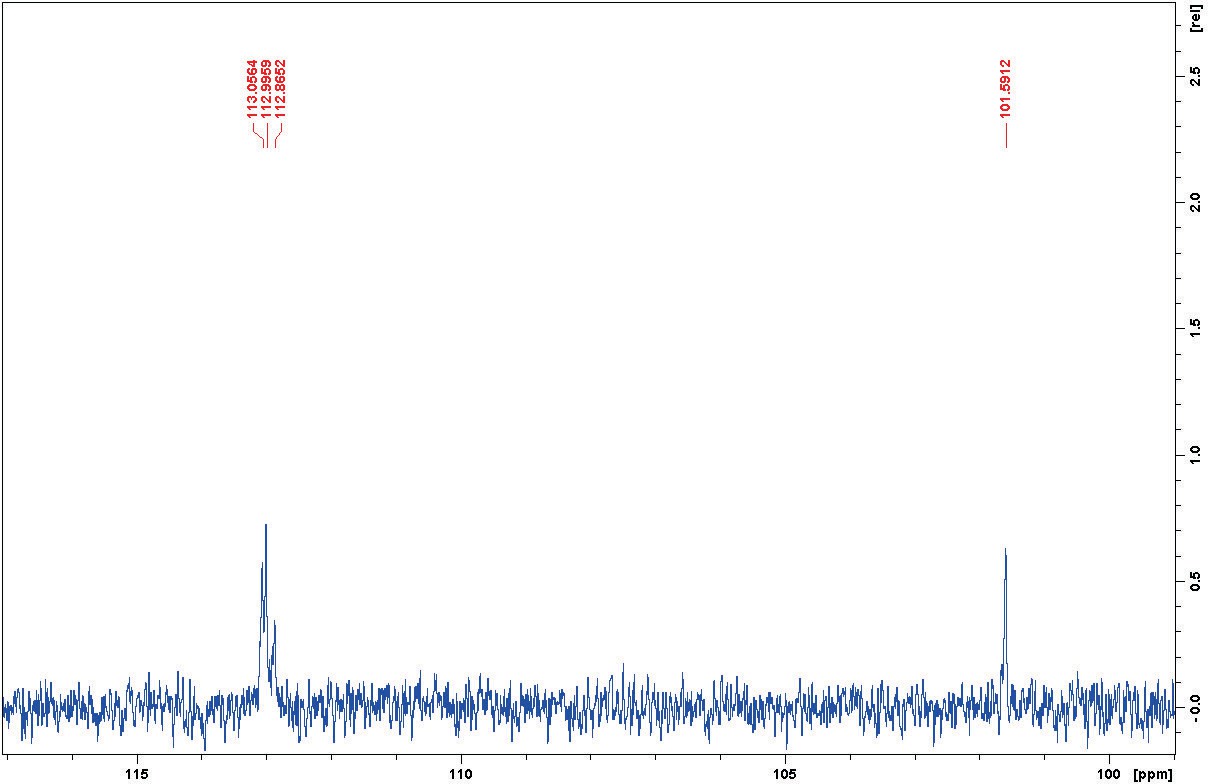


**Figure** **S58**: ^13^CNMR spectrum of **4** in Pyridine d5 (Exp.)


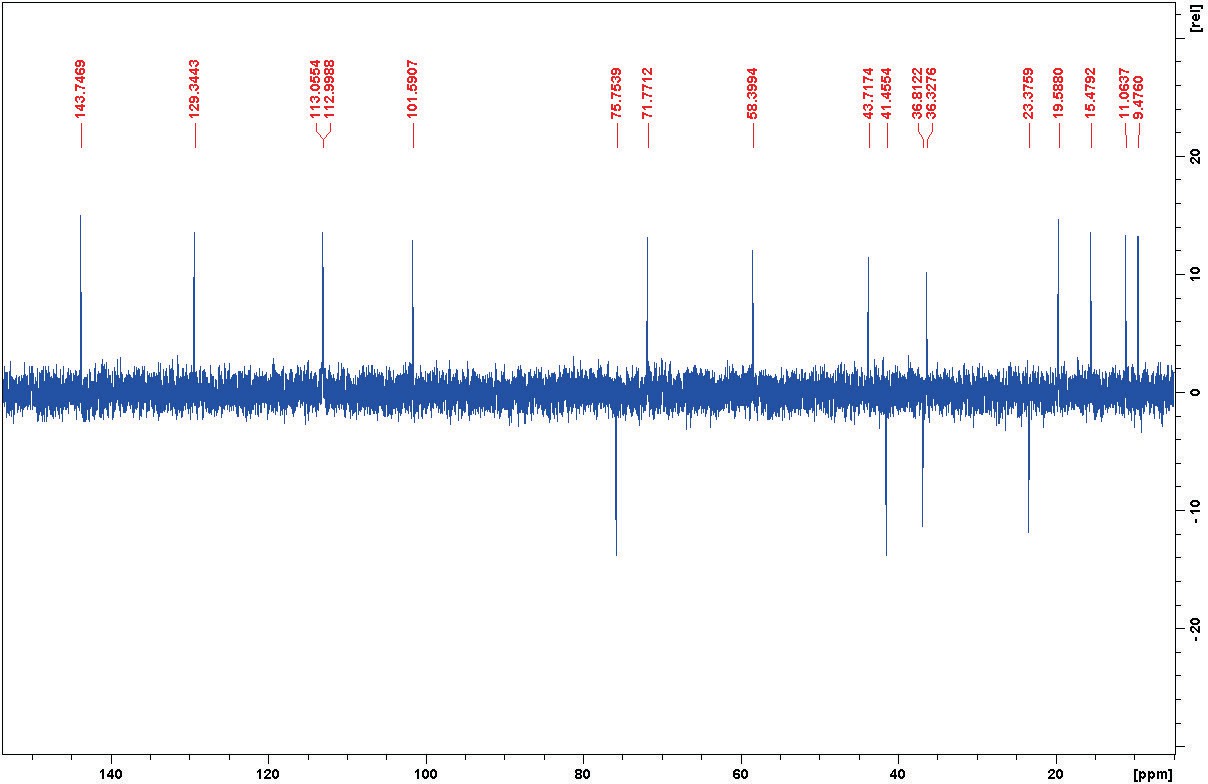


**Figure** **S59**: DEPT135 spectrum of **4** in Pyridine d5.


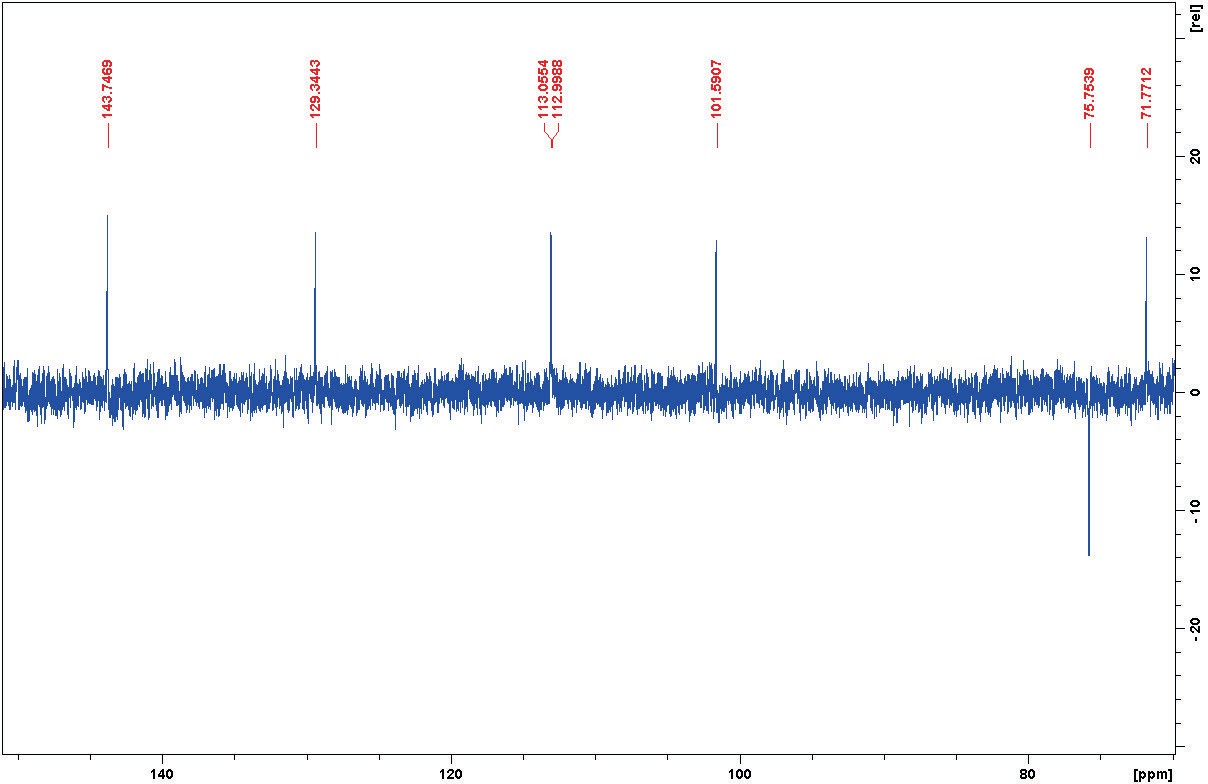


**Figure** **S60**: DEPT135 spectrum of **4** in Pyridine d5 (Exp.).


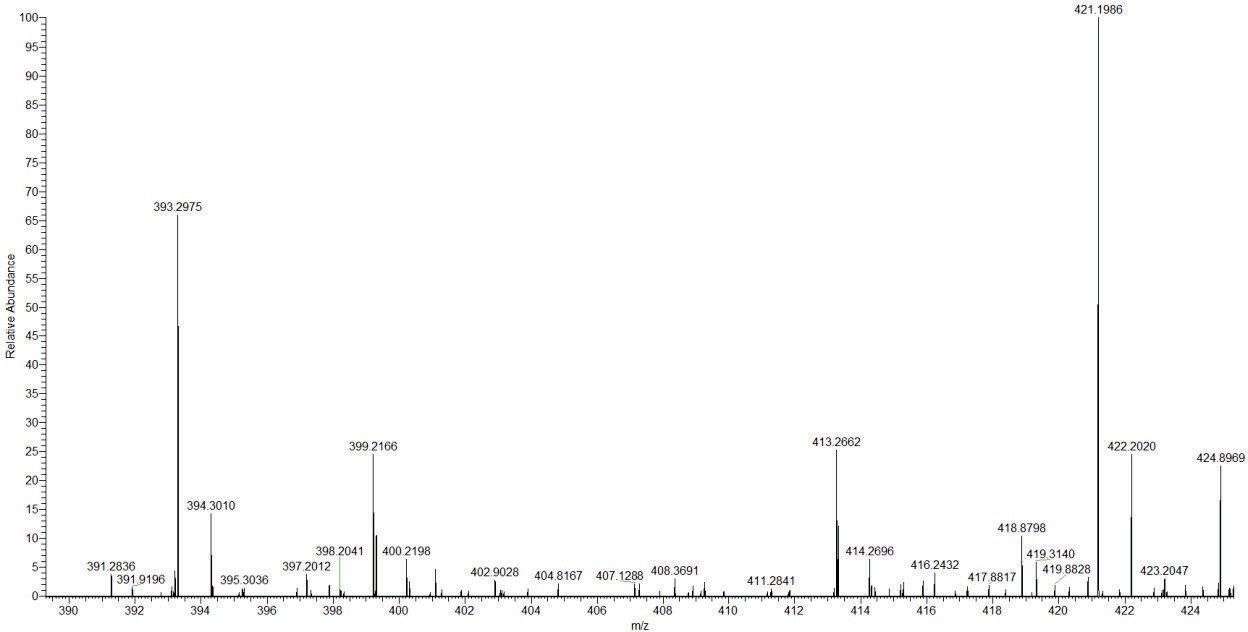


**Figure** **S61**: HRESIMS spectrum of **4**.
